# Global trends of early-onset Parkinson’s disease from 1990 to 2021, and projections until to 2030: a systematic analysis of the global burden of disease study 2021

**DOI:** 10.3389/fneur.2025.1589760

**Published:** 2025-08-01

**Authors:** Bingtao Hu, Qi Mao, Bin Liang, Yunhui Li

**Affiliations:** ^1^Department of Laboratory Medical Center, General Hospital of Northern Theater Command, Shenyang, China; ^2^Department of Bioinformatics, Key Laboratory of Cell Biology, Ministry of Public Health, Key Laboratory of Medical Cell Biology, Ministry of Education, School of Life Sciences, China Medical University, Shenyang, China

**Keywords:** Parkinson’s disease, neurodegenerative disease, global disease burden, trends, health inequality analysis

## Abstract

**Background:**

This study aims to evaluate the global, regional, and national trends in prevalence, incidence, mortality, and DALYs for early-onset Parkinson’s disease (EOPD) from 1990 to 2021.

**Methods:**

All data from the Global Burden of Diseases (GBD) 2021 were stratified by age, sex, location, and socio-demographic index (SDI). We performed comprehensive analysis to reveal trends for EOPD, utilizing average annual percentage change (AAPC), joinpoint regression analysis, health inequality analysis, frontier analysis, and Bayesian age-period-cohort (BAPC) models.

**Results:**

In 2021, the global case numbers of prevalence, incidence, mortality, and DALYs for EOPD were 483872.47 (95% UI: 328861.91–682509.05), 81046.67 (95% UI: 48161.87–122328.00), 2245.68 (95% UI: 1995.24–2495.67), and 180325.32 (95% UI: 145990.30–225031.04). The age-standardized prevalence rate (ASPR), age-standardized incidence rate (ASIR), and age-standardized DALYs rate (ASDR) keep increasing from 1990 to 2021, and the age-standardized death rate (ASMR) occurred the decline trend. Male showed higher ASPR, ASIR, ASMR, and ASDR across all age groups. The geographical disparities at regional and national level were evident. Significant disparities in EOPD burden by SDI were observed, exhibiting a worsening inequality over time. The frontier analysis lists some countries and territories requiring urgent action to mitigate EOPD burden. Furthermore, it was anticipated that the ASPR and ASIR would consistently increase during 2021 to 2030, while the ASMR and ASDR would decrease.

**Conclusion:**

EOPD demonstrated notable heterogeneity across age, gender, and geography. The increasing burden of EOPD underscored the urgent need for targeted public health strategies and policies, especially in undeveloped regions.

## Introduction

In 2022, the World Health Organization (WHO) issued a technical brief, “Parkinson’s disease: a public health approach,” outlining the global burden, treatment gaps, and crucial areas for action for PD. The issue emphasized that death and disability due to Parkinson’s disease (PD) are increasing at the fastest rate in all neurological disorder, and the corresponding global burden has more than doubled over the past generation as a result of the aging of the population and increased life expectancy (1, 2). Early-onset PD (EOPD), refers to PD cases with onset age between the age of 21 and 50 years old, recommended by International Parkinson and Movement Disorder Society (MDS) (3). Because the earlier EOPD symptoms and longer time horizon of disease in life, it often has an extraordinary impact on their personal, family, social, and professional life. Therefore, the people with EOPD face unique and significant challenges compared with those with late-onset PD (LOPD).

EOPD presents significant dissimilarities compared to the LOPD starting at 50–70 years old. In EOPD patients, genetic variation and sex difference very likely contribute to age-at-onset variation, and up to 10% EOPD patients harbor a major genetic determinant (4, 5). The clinicians often take long time to conclusively diagnose EOPD patients due to younger age, with an average of an additional 15 months in comparison with regular-onset PD (6). EOPD patients have a different disease progression, clinical characteristics, response to medication, and pathology features, reflecting a different pathophysiological background with LOPD (7). Moreover, EOPD affects the patients in the prime of their life, and thus impacts social, occupational, and familial dynamics.

Based on the limited literature, the epidemiological estimates of EOPD varied widely across the studies. The diagnostic criteria and consensus on the age of EOPD were different in study populations; however, the differences of genetic and environmental factors affected the prevalence, incidence, and mortality. Most studies provided more detailed information on EOPD for a single country or region or specific age group. In this study, we analyzed the data from the Global Burden of Diseases, Injuries, and Risk Factors Study (GBD) 2021, and evaluated the trends of prevalence, incidence, mortality, and disability-adjusted life years (DALYs) for EOPD, as well as the socio-demographic, spatial, and temporal variations in EOPD burden. Through this comprehensive evaluation of global EOPD measures, we hope to provide insights for tailored policies and targeted strategies concerning prevention, diagnosis, control, and health resource allocation.

## Methods

### GBD 2021 overview

GBD 2021 provides extensive epidemiological data estimates for 371 diseases and injuries, leverages 328,938 data sources, reveals health disparities across age, sex, location, and socioeconomic groups, spanning the period from 1990 to 2021. Data from the GBD 2021 study are publicly available online through the Global Health Data Exchange platform.^1^ All detailed information on the data, methodologies, and statistical modeling have been extensively described previously (8). This analysis complies with the Guidelines for Accurate and Transparent Health Estimates Reporting (GATHER).

### Data sources

According to the International Classification of Diseases (ICD), 9th and 10th editions, PD is represented by codes 332-332.0 and F02.3, G20-G20.9, respectively. In GBD 2021, PD is defined by the presence of at least two of the following four primary symptoms: (1) tremors or trembling, (2) bradykinesia, (3) stiffness of limbs and torso, (4) postural instability, and the case reference definition also accept alternative definition, the UK Parkinson’s Disease Society Brain Bank criteria (2, 9). More detailed information on data processing can be found on the GBD data website^2^ and related publications from the GBD Parkinson’s Disease and Neurological Disorders collaboration (2, 10). In this study, the number and rate for prevalence, incidence, mortality and DALYs relating to PD by age group (20–24 years, 25–29 years, 30–34 years, 35–39 years, 40–44 years, and 45–49 years), sex (both, male, and female), location (global, SDI regions, 21 GBD regions, and 204 countries and territories) from 1990 to 2021 were downloaded from the GBD 2021 database.

### Measures

GBD 2021 data were extracted from vital registration systems, verbal autopsies, censuses, household surveys, disease-specific registries, health service contact data, and other sources (8). The main measures in our study were prevalence, incidence, mortality, and DALYs of EOPD. The consistency of prevalence and incidence data was ensured through the DisMod-MR 2.1, a Bayesian meta-regression modeling tool. DisMod-MR 2.1 evaluated and pooled all available data from various sources, adjusted data for systematic bias associated with case ascertainment methods that varied from the reference and produced estimates by world regions with uncertainty interval (UIs) by using Bayesian statistical methods. This systematic approach to data correction helps to minimize the impact of data heterogeneity on the study outcomes, enhancing the reliability of the results. The mortality attributable to each cause is the product of the attributable fraction and the mortality due to the underlying disease, and is analyzed using a highly systematized Cause of Death Ensemble model (CODEm), a modeling tool developed for GBD to assess the out-of-sample predictive validity of different statistical models and covariate permutations and combine those results to produce cause-specific mortality estimates (11, 12). Moreover, DALYs was used to compare deaths and non-fatal outcomes within and between diseases, which is the sum of years lost due to premature death and the sum of years lived with disability. All estimates were reported as numbers and rates (per 100,000 populations) along with 95% UI, and the 95% UI is determined by the 2.5th and 97.5th percentiles of these 500 ordered draws (13). Age-standardized rates were calculated using the GBD world population age standard.

### Socio-demographic index

Socio-demographic index (SDI) is a comprehensive index of lag-distributed income per capita, average years of education for those aged 15 years or older, and fertility rates among females younger than 25 years. SDI is mainly used to evaluate the development level of regions and countries (14). SDI values ranged from 0 to 1.0. In the GBD 2021, the 204 countries and territories were classified into SDI quintiles: low SDI (0–0.4658), low-middle SDI (0.4658–0.6188), middle SDI (0.6188–0.7119), high-middle SDI (0.7119–0.8102), and high SDI (0.8102–1.0).

### Data analysis

We adopted the joinpoint regression analysis model, a statistical methodology developed by the SEER Program of the National Cancer Institute of the United States, to expertly identified and quantitatively calculated the points in trends for significant changes in prevalence, incidence, mortality, and DALYs for EOPD. The optimal number of join points was determined using the Monte Carlo permutation test, allowing for a maximum of 5 join points and a minimum of 0. The results of the joinpoint regression model were expressed as annual percentage change (APC) and average annual percentage change (AAPC), providing the assessment of internal trends over each independent time period. An APC or AAPC, with its 95% CI, greater than 0 indicates that the burden of disease has tended to increase over time during the period; conversely, that the burden of disease has tended to decrease. If the 95% CI for the APC or AAPC includes zero, it suggests that the trend has remained stable.

We employed the slope index of inequality (SII) and concentration index (CIX), as recommended by the World Health Organization (WHO), to measure the absolute and relative inequality across 204 countries and territories from 1990 to 2021. The SII was calculated by performing a weighted regression analysis of ASR due to EOPD on a relative position scale, which is defined by the midpoint of the cumulative range of the population ranked by SDI. The CIX was calculated by fitting a Lorenz concentration curve to the observed cumulative relative distribution of the populations ranked by the SDI and age-standardized rate (ASR) and numerically integrating the area under the curve (15). To better control for bias and heterogeneity, we adopted a robust regression model (rlm) instead of the ordinary linear regression model (lm) in this health inequality analysis. The robust regression model reduces sensitivity to outliers, minimizing bias caused by data heterogeneity or extreme values and leading to a more accurate representation of health inequality. The positive SII means that the disease burden is concentrated in higher SDI regions, while the negative SII reflects the disease burden mainly is concentrated in low SDI regions. A positive CIX indicates that the health variable is more concentrated among affluent groups, while a negative CIX indicates higher concentration among impoverished groups. The closer the CIX is to zero, the more equitable the distribution of the health variable across socioeconomic groups. When the CIX values is increasing or decreasing, health inequality is said to have widened or narrowed over time, respectively.

We adopted the frontier analysis to assess the relationship between EOPD burden and socio-demographic development. Frontier analysis used non-parametric Data Envelop Analysis (DEA) to generate non-linear frontier, representing the lowest ASR each country and territory could potentially achieve based on the development status measured by the SDI. We integrated DALYs rate with the SDI, and performed frontier analysis to assess the potential development space in each country and territory. To ensure the robustness of the analysis, we conducted 1,000 bootstrap samples and calculated the average ASR for each SDI value. If the observed ASR is well below the frontier line of its SDI, it implies that there may be substantial unrecognized opportunities for improvement in ASR. When the observed ASR is closer to the frontier line at its SDI, it implies that this country or territory is top-performing, and could provide the reference to other countries.

Bayesian age-period-cohort (BAPC) model is flexible and robust in handling time series data, making it particularly suitable for long-term disease burden predictions. In view of its comprehensive coverage and ability to capture temporal trends, BAPC model has been widely applied in epidemiological research, especially in studies involving age-structured population data and complex cohort effects. In this analysis, we leveraged GBD 2021 data and demographic projections from the IHME, and adopted BAPC model integrated nested Laplace approximations to forecast the prevalence, incidence, mortality, and DALYs rates of EOPD in the next decade. This method enables nuanced predictions of EOPD burdens while considering the intricate interactions of age, period, and cohort effects.

In this study, all analyses were performed using R software (Version 4.3.1), Joinpoint software (Version 5.0), and Jingding software (Version 2.34.2). A *p*-value <0.05 was considered significant.

## Results

### Global trends

Globally, the case numbers of prevalence for EOPD were 483872.47 (95% UI: 328861.91–682509.05) in 2021. The age-standardized prevalence rate (ASPR) was 14.00 (95% UI: 9.49–19.79) per 100,000 populations, with an AAPC of 1.07 (95% CI: 1.01–1.13) from 1990 to 2021. The case number of incidence for EOPD was 81046.67 (95% UI: 48161.87–122328.00) in 2021. The age-standardized incidence rate (ASIR) was 2.35 (95% UI: 1.39–3.55) per 100,000 populations, with an AAPC of 1.51 (95% CI: 1.43–1.60). In mortality, the number and age-standardized death rate (ASMR) of EOPD were 2245.68 (95% UI: 1995.24–2495.67) and 0.06 (95% UI: 0.06–0.07) per 100,000 populations in 2021, respectively. The ASMR presented the significant decline trend, with the AAPC of −0.41 (95% CI: −0.52 to −0.29). Global age-standardized DALY rate (ASDR), reflecting the burden of disease, increased by an AAPC of 0.15 (95% CI: 0.08–0.22) (Tables 1–4).

**Table 1:** The case number and age-standardized rate of prevalence for both sexes, SDI regions, and 21 GBD regions, with the AAPCs between 1990 and 2021.

	1990		2021		
Location	Number (95% UI)	ASPR per 100,000 populations (95% UI)	Number (95% UI)	ASPR per 100,000 populations (95% UI)	AAPCs (95% CI)
Global	190486.50 (121780.55–280338.47)	10.03 (6.50–14.63)	483872.47 (328861.91–682509.05)	14.00 (9.49–19.79)	1.07^*^ (1.01–1.13)
Male	113692.45 (73379.31–166429.16)	11.73 (7.67–17.04)	291072.89 (199491.69–405673.45)	16.74 (11.44–23.36)	1.14^*^ (1.08–1.2)
Female	76794.06 (48047.54–114619.34)	8.26 (5.26–12.20)	192799.59 (129140.43–277911.64)	11.23 (7.49–16.23)	0.99^*^ (0.92–1.05)
SDI regions					
Low SDI	13146.19 (8141.90–19681.10)	9.51 (6.01–14.08)	36135.64 (22909.84–53200.24)	10.50 (6.77–15.31)	0.31^*^ (0.27–0.35)
Low-middle SDI	38537.38 (24423.77–56656.68)	10.68 (6.89–15.56)	99358.01 (65198.11–143977.93)	12.95 (8.56–18.69)	0.60^*^ (0.54–0.66)
Middle SDI	61560.69 (39385.89–89906.93)	10.47 (6.83–15.13)	188369.30 (128754.69–263874.49)	16.15 (10.99–22.70)	1.39^*^ (1.32–1.47)
High-middle SDI	38432.78 (24405.56–56978.10)	9.35 (6.03–13.73)	106896.06 (72767.31–152703.50)	15.78 (10.65–22.69)	1.68^*^ (1.61–1.76)
High SDI	38639.82 (25066.07–56955.47)	9.70 (6.31–14.27)	52827.07 (36616.17–73198.05)	10.26 (7.04–14.31)	0.18^*^ (0.14–0.23)
GBD regions					
Andean Latin America	2724.30 (1746.61–3939.43)	23.53 (15.36–33.56)	8441.62 (5631.50–12220.02)	30.22 (20.24–43.61)	0.79^*^ (0.75–0.83)
Australasia	390.27 (199.09–662.80)	4.26 (2.18–7.21)	765.80 (431.56–1245.35)	5.51 (3.07–9.03)	0.87^*^ (0.77–0.97)
Caribbean	1612.29 (1009.80–2425.77)	13.04 (8.29–19.44)	3050.89 (1943.99–4476.97)	14.90 (9.46–21.90)	0.41^*^ (0.33–0.5)
Central Asia	1460.12 (820.01–2398.57)	7.34 (4.32–11.75)	3015.44 (1775.57–4712.43)	7.31 (4.34–11.37)	−0.02^*^ (−0.03 to −0.01)
Central Europe	3739.39 (2250.12–5670.17)	6.96 (4.20–10.53)	3588.24 (2336.49–5215.31)	6.03 (3.86–8.87)	−0.46^*^ (−0.51 to −0.41)
Central Latin America	7152.58 (4513.64–10497.20)	14.32 (9.25–20.72)	19536.05 (12944.66–28144.18)	17.41 (11.52–25.10)	0.62^*^ (0.6–0.65)
Central Sub-Saharan Africa	1176.42 (665.60–1846.35)	8.24 (4.81–12.69)	3781.30 (2236.42–5869.86)	9.17 (5.54–14.05)	0.35^*^ (0.34–0.36)
East Asia	44375.91 (28233.66–65472.51)	9.73 (6.32–14.18)	166105.93 (115257.24–233537.65)	21.34 (14.68–30.19)	2.55^*^ (2.45–2.66)
Eastern Europe	7708.92 (4752.35–11669.01)	8.63 (5.40–12.94)	9013.73 (5784.77–13304.36)	8.61 (5.46–12.80)	−0.01 (−0.03 to 0.02)
Eastern Sub-Saharan Africa	4377.58 (2660.98–6625.79)	9.05 (5.66–13.48)	12018.50 (7427.32–18033.97)	9.40 (5.94–13.92)	0.12^*^ (0.11–0.14)
High-income Asia Pacific	6323.53 (3935.99–9556.27)	7.44 (4.57–11.31)	7586.32 (4855.93–11311.89)	8.25 (5.11–12.54)	0.33^*^ (0.27–0.38)
High-income North America	12554.48 (8125.48–18472.79)	9.89 (6.45–14.48)	11156.25 (8624.17–14068.61)	7.03 (5.40–8.89)	−1.09^*^ (−1.15 to −1.04)
North Africa and Middle East	8501.51 (5155.22–12977.10)	8.67 (5.39–13.02)	32135.51 (20412.83–47582.72)	11.37 (7.24–16.78)	0.87^*^ (0.84–0.9)
Oceania	156.71 (91.32–247.74)	7.83 (4.69–12.17)	448.40 (267.66–689.90)	8.65 (5.22–13.21)	0.32^*^ (0.29–0.34)
South Asia	40090.92 (25364.36–58664.77)	11.20 (7.20–16.26)	107011.67 (69536.23–153542.11)	13.81 (9.03–19.76)	0.65^*^ (0.57–0.73)
Southeast Asia	13874.20 (8668.92–20649.58)	9.16 (5.87–13.45)	33111.56 (21639.66–48543.10)	10.16 (6.62–14.92)	0.33^*^ (0.32–0.35)
Southern Latin America	1065.78 (575.68–1721.61)	5.54 (3.01–8.92)	2150.11 (1210.86–3397.46)	6.92 (3.88–10.98)	0.72^*^ (0.62–0.81)
Southern Sub-Saharan Africa	1436.00 (903.10–2141.69)	9.08 (5.86–13.37)	3268.05 (2119.51–4797.82)	9.70 (6.36–14.15)	0.20^*^ (0.16–0.25)
Tropical Latin America	6942.31 (4460.09–10050.72)	13.27 (8.67–19.02)	18359.84 (12248.50–26034.02)	16.75 (11.14–23.80)	0.71^*^ (0.52–0.91)
Western Europe	20594.68 (13371.61–30130.95)	12.13 (7.86–17.75)	26957.73 (17782.11–39316.99)	13.68 (8.89–20.17)	0.39^*^ (0.35–0.43)
Western Sub-Saharan Africa	4228.60 (2577.60–6362.12)	7.94 (4.94–11.82)	12369.53 (7654.42–18540.48)	8.67 (5.47–12.84)	0.29^*^ (0.27–0.32)

UI, uncertainty interval; CI, confidence interval; AAPC, average annual percent of change; ASPR, age-standardized prevalence rate; SDI, socio-demographic index. *indicates *p* < 0.05.

**Table 2:** The case number and age-standardized rate of incidence for both sexes, SDI regions, and 21 GBD regions, with the AAPCs between 1990 and 2021.

	1990		2021		
Location	Number (95% UI)	ASIR per 100,000 populations (95% UI)	Number (95% UI)	ASIR per 100,000 populations (95% UI)	AAPC (95% CI)
Global	28266.73 (15154.79–45134.23)	1.46 (0.79–2.32)	81046.67 (48161.87–122328.00)	2.35 (1.39–3.55)	1.51^*^ (1.43–1.6)
Male	16438.49 (8886.70–26034.64)	1.67 (0.91–2.63)	48417.29 (28943.52–72816.64)	2.79 (1.67–4.20)	1.65^*^ (1.56–1.74)
Female	11828.24 (6133.38–19047.20)	1.25 (0.66–2.00)	32629.38 (19138.04–50081.59)	1.90 (1.12–2.93)	1.34^*^ (1.23–1.44)
SDI regions					
Low SDI	1830.39 (927.12–3001.09)	1.28 (0.66–2.08)	5073.36 (2647.32–8137.05)	1.43 (0.76–2.29)	0.35^*^ (0.29–0.4)
Low-middle SDI	5526.83 (2929.40–8847.20)	1.49 (0.81–2.38)	14599.23 (8292.46–22866.02)	1.88 (1.08–2.95)	0.73^*^ (0.65–0.8)
Middle SDI	9373.40 (5080.24–14808.66)	1.56 (0.86–2.44)	32573.99 (19657.32–48825.40)	2.81 (1.69–4.21)	1.87^*^ (1.75–1.99)
High-middle SDI	6057.92 (3216.22–9702.56)	1.46 (0.78–2.32)	20352.09 (12304.12–30496.68)	3.02 (1.82–4.55)	2.37^*^ (2.26–2.48)
High SDI	5453.33 (2891.92–8734.16)	1.37 (0.72–2.20)	8405.65 (5068.04–12457.87)	1.65 (0.98–2.45)	0.60^*^ (0.56–0.64)
GBD regions					
Andean Latin America	412.20 (239.94–621.48)	3.45 (2.05–5.14)	1429.29 (864.28–2110.61)	5.10 (3.09–7.51)	1.26^*^ (1.24–1.29)
Australasia	53.75 (20.17–98.07)	0.59 (0.22–1.07)	105.86 (45.11–191.46)	0.77 (0.33–1.40)	0.92^*^ (0.83–1.02)
Caribbean	228.30 (121.55–368.41)	1.81 (0.98–2.89)	433.78 (236.09–691.03)	2.13 (1.16–3.39)	0.51^*^ (0.42–0.61)
Central Asia	226.11 (100.56–399.07)	1.10 (0.51–1.90)	442.22 (206.73–766.90)	1.07 (0.50–1.85)	−0.10^*^ (−0.13 to −0.07)
Central Europe	564.12 (273.74–936.15)	1.05 (0.51–1.75)	575.83 (316.10–907.42)	0.98 (0.53–1.55)	−0.25^*^ (−0.29 to −0.21)
Central Latin America	1022.89 (550.20–1636.30)	1.97 (1.09–3.12)	2819.48 (1611.25–4328.03)	2.52 (1.44–3.87)	0.77^*^ (0.66–0.87)
Central Sub-Saharan Africa	171.86 (80.42–295.96)	1.16 (0.56–1.96)	543.46 (261.78–932.56)	1.28 (0.63–2.19)	0.33^*^ (0.31–0.36)
East Asia	7402.71 (4047.92–11683.10)	1.60 (0.90–2.50)	34920.25 (21652.84–51175.57)	4.54 (2.81–6.68)	3.39^*^ (3.32–3.47)
Eastern Europe	1236.68 (623.11–1996.53)	1.38 (0.70–2.21)	1420.56 (733.20–2256.54)	1.37 (0.70–2.20)	0.01 (−0.04 to 0.06)
Eastern Sub-Saharan Africa	605.25 (296.71–1014.62)	1.20 (0.60–1.99)	1646.95 (811.49–2690.53)	1.24 (0.62–2.01)	0.12^*^ (0.1–0.14)
High-income Asia Pacific	897.98 (444.03–1463.80)	1.07 (0.52–1.75)	1036.26 (513.92–1677.36)	1.17 (0.57–1.91)	0.28^*^ (0.2–0.35)
High-income North America	1760.24 (922.56–2781.63)	1.38 (0.72–2.18)	1920.55 (1321.43–2604.55)	1.21 (0.83–1.65)	−0.38^*^ (−0.43 to −0.34)
North Africa and Middle East	1354.01 (725.63–2206.64)	1.35 (0.74–2.17)	5121.91 (2964.67–8055.55)	1.81 (1.05–2.84)	0.94^*^ (0.9–0.98)
Oceania	25.85 (13.01–43.30)	1.27 (0.66–2.10)	73.07 (37.76–119.52)	1.40 (0.73–2.28)	0.32^*^ (0.27–0.37)
South Asia	5582.70 (2839.79–8942.84)	1.52 (0.78–2.43)	15224.84 (8373.30–23964.95)	1.95 (1.08–3.06)	0.76^*^ (0.66–0.87)
Southeast Asia	2026.20 (1032.85–3274.96)	1.30 (0.67–2.08)	4706.15 (2500.59–7524.72)	1.45 (0.77–2.32)	0.37^*^ (0.34–0.4)
Southern Latin America	163.27 (68.47–287.25)	0.85 (0.36–1.49)	327.35 (145.34–557.80)	1.06 (0.47–1.81)	0.72^*^ (0.59–0.85)
Southern Sub-Saharan Africa	196.93 (99.84–318.57)	1.20 (0.62–1.92)	433.79 (221.53–699.30)	1.27 (0.65–2.04)	0.19^*^ (0.17–0.22)
Tropical Latin America	1002.05 (549.03–1559.68)	1.86 (1.03–2.88)	2648.10 (1507.55–4039.82)	2.43 (1.38–3.71)	0.85^*^ (0.62–1.07)
Western Europe	2786.90 (1477.01–4511.07)	1.64 (0.87–2.66)	3597.42 (1935.84–5795.28)	1.86 (0.99–3.01)	0.40^*^ (0.37–0.42)
Western Sub-Saharan Africa	546.72 (254.58–920.49)	0.98 (0.46–1.65)	1619.57 (776.54–2698.07)	1.09 (0.53–1.81)	0.35^*^ (0.31–0.39)

UI, uncertainty interval; CI, confidence interval; AAPC, average annual percent of change; ASIR, age-standardized incidence rate; SDI, socio-demographic index. *indicates *p* < 0.05.

**Table 3:** The case number and age-standardized rate of death for both sexes, SDI regions, and 21 GBD regions, with the AAPCs between 1990 and 2021.

	1990		2021		
Location	Number (95% UI)	ASMR per 100,000 populations (95% UI)	Number (95% UI)	ASMR per 100,000 populations (95% UI)	AAPC (95% CI)
Global	1335.36 (1160.20–1470.43)	0.07 (0.06–0.08)	2245.68 (1995.24–2495.67)	0.06 (0.06–0.07)	−0.41^*^ (−0.52 to −0.29)
Male	832.07 (730.36–948.29)	0.09 (0.08–0.10)	1475.44 (1277.38–1685.51)	0.08 (0.07–0.10)	−0.16^*^ (−0.23 to −0.09)
Female	503.29 (385.30–585.25)	0.06 (0.04–0.07)	770.24 (625.27–934.27)	0.04 (0.04–0.05)	−0.74^*^ (−0.96 to −0.51)
SDI regions					
Low SDI	93.77 (74.02–116.70)	0.07 (0.06–0.09)	218.69 (169.81–270.07)	0.07 (0.05–0.08)	−0.24^*^ (−0.35 to −0.13)
Low-middle SDI	235.30 (194.24–279.63)	0.07 (0.06–0.08)	520.46 (442.67–601.54)	0.07 (0.06–0.08)	0.06 (−0.04 to 0.16)
Middle SDI	504.33 (421.95–563.23)	0.09 (0.08–0.10)	829.82 (724.68–947.27)	0.07 (0.06–0.08)	−0.78^*^ (−0.95 to −0.61)
High-middle SDI	306.82 (265.97–346.76)	0.08 (0.07–0.09)	399.76 (344.69–471.76)	0.06 (0.05–0.07)	−0.92^*^ (−1.19 to −0.65)
High SDI	194.10 (188.18–200.17)	0.05 (0.05–0.05)	275.29 (258.78–292.71)	0.05 (0.05–0.06)	0.15 (−0.03 to 0.33)
GBD regions					
Andean Latin America	7.71 (6.46–9.01)	0.07 (0.06–0.08)	15.61 (12.03–19.66)	0.06 (0.04–0.07)	−0.63^*^ (−1.18 to −0.08)
Australasia	3.00 (2.79–3.23)	0.03 (0.03–0.04)	5.46 (5.04–5.92)	0.04 (0.04–0.04)	0.42^*^ (0.12–0.71)
Caribbean	7.33 (6.30–8.30)	0.06 (0.05–0.07)	13.99 (11.48–16.98)	0.07 (0.06–0.08)	0.42^*^ (0.12–0.72)
Central Asia	9.71 (8.92–10.61)	0.05 (0.05–0.05)	17.08 (14.90–19.37)	0.04 (0.04–0.05)	−0.49^*^ (−0.85 to −0.13)
Central Europe	26.15 (25.08–27.32)	0.05 (0.05–0.05)	23.56 (21.60–25.65)	0.04 (0.04–0.04)	−0.91^*^ (−1.23 to −0.6)
Central Latin America	34.85 (33.73–36.02)	0.07 (0.07–0.07)	77.15 (68.35–86.29)	0.07 (0.06–0.08)	−0.2 (−0.51 to 0.12)
Central Sub-Saharan Africa	8.64 (6.04–11.22)	0.07 (0.05–0.09)	23.43 (16.55–31.32)	0.06 (0.04–0.08)	−0.30^*^ (−0.41 to −0.19)
East Asia	513.35 (409.85–595.13)	0.12 (0.09–0.14)	593.18 (474.62–737.27)	0.08 (0.06–0.10)	−1.36^*^ (−1.46 to −1.25)
Eastern Europe	33.28 (31.55–35.74)	0.04 (0.04–0.04)	44.46 (39.98–48.99)	0.04 (0.04–0.05)	0.3 (−0.22 to 0.83)
Eastern Sub-Saharan Africa	27.95 (21.52–34.41)	0.06 (0.05–0.08)	62.86 (46.81–80.47)	0.05 (0.04–0.07)	−0.57^*^ (−0.65 to −0.5)
High-income Asia Pacific	39.12 (36.77–41.72)	0.04 (0.04–0.05)	40.58 (38.13–43.33)	0.04 (0.04–0.04)	−0.4 (−0.84 to 0.05)
High-income North America	62.62 (61.19–64.16)	0.05 (0.05–0.05)	97.39 (94.10–100.75)	0.06 (0.06–0.06)	0.47^*^ (0.24–0.7)
North Africa and Middle East	84.63 (68.29–101.07)	0.09 (0.07–0.11)	209.43 (169.72–251.18)	0.08 (0.06–0.09)	−0.62^*^ (−0.69 to −0.55)
Oceania	1.26 (0.83–1.70)	0.07 (0.04–0.09)	2.85 (2.10–4.06)	0.06 (0.04–0.08)	−0.47^*^ (−0.61 to −0.33)
South Asia	202.36 (161.11–260.25)	0.06 (0.05–0.08)	460.29 (376.44–550.43)	0.06 (0.05–0.07)	0.10^*^ (0.05–0.14)
Southeast Asia	123.69 (104.49–141.68)	0.09 (0.07–0.10)	285.47 (241.39–337.59)	0.09 (0.07–0.10)	−0.01 (−0.12 to 0.1)
Southern Latin America	8.40 (7.83–9.00)	0.04 (0.04–0.05)	11.30 (10.43–12.22)	0.04 (0.03–0.04)	−0.63^*^ (−0.84 to −0.42)
Southern Sub-Saharan Africa	9.96 (8.60–11.53)	0.07 (0.06–0.07)	25.56 (21.12–29.92)	0.08 (0.06–0.09)	0.49^*^ (0.28–0.71)
Tropical Latin America	25.42 (24.56–26.33)	0.05 (0.05–0.05)	56.81 (54.72–58.98)	0.05 (0.05–0.05)	0.06 (−0.18 to 0.3)
Western Europe	65.59 (63.62–67.72)	0.04 (0.04–0.04)	66.15 (63.97–68.44)	0.03 (0.03–0.03)	−0.68^*^ (−1.02 to −0.35)
Western Sub-Saharan Africa	40.35 (31.32–49.75)	0.08 (0.06–0.10)	113.10 (74.36–172.65)	0.08 (0.05–0.13)	0.08 (−0.03 to 0.19)

UI, uncertainty interval; CI, confidence interval; AAPC, average annual percent of change; ASMR, age-standardized death rate; SDI, socio-demographic index. *indicates *p* < 0.05.

**Table 4:** The case number and age-standardized rate of DALYs for both sexes, SDI regions, and 21 GBD regions, with the AAPCs between 1990 and 2021.

	1990		2021		
Location	Number (95% UI)	ASDR per 100,000 populations (95% UI)	Number (95% UI)	ASDR per 100,000 populations (95% UI)	AAPC (95% CI)
Global	92575.05 (76441.04–112394.15)	4.97 (4.13–6.00)	180325.32 (145990.30–225031.04)	5.22 (4.22–6.52)	0.15^*^ (0.08–0.22)
Male	56869.23 (46797.10–69205.71)	5.99 (4.96–7.25)	114178.43 (91723.03–141330.67)	6.57 (5.27–8.13)	0.30^*^ (0.23–0.36)
Female	35705.82 (27844.22–44898.85)	3.92 (3.08–4.89)	66146.90 (51896.51–85190.92)	3.85 (3.01–4.96)	−0.07 (−0.14 to 0.01)
SDI regions					
Low SDI	6471.72 (5046.80–8191.63)	4.83 (3.80–6.07)	16030.26 (12206.05–20541.65)	4.75 (3.64–6.05)	−0.03 (−0.11 to 0.05)
Low-middle SDI	17112.19 (13622.85–21395.66)	4.83 (3.88–6.00)	40011.35 (31991.00–50316.11)	5.25 (4.21–6.59)	0.25^*^ (0.2–0.31)
Middle SDI	33336.76 (27383.58–39861.40)	5.82 (4.81–6.91)	67961.50 (54799.33–85364.52)	5.83 (4.69–7.34)	0.01 (−0.05 to 0.07)
High-middle SDI	20401.98 (16704.01–24716.09)	5.09 (4.19–6.13)	35210.86 (27709.48–46132.08)	5.21 (4.09–6.84)	0.08 (−0.03 to 0.18)
High SDI	15175.79 (12359.00–18996.96)	3.84 (3.14–4.80)	20988.81 (17483.62–25486.68)	4.02 (3.33–4.91)	0.13^*^ (0.03–0.23)
GBD regions					
Andean Latin America	797.10 (578.28–1089.96)	6.90 (5.05–9.36)	2051.69 (1423.85–2970.70)	7.34 (5.10–10.61)	0.2 (−0.01 to 0.41)
Australasia	201.46 (163.08–253.71)	2.23 (1.81–2.79)	373.54 (303.09–470.67)	2.65 (2.14–3.35)	0.60^*^ (0.3–0.91)
Caribbean	607.67 (470.16–778.56)	4.90 (3.81–6.26)	1140.60 (875.29–1469.44)	5.55 (4.25–7.16)	0.40^*^ (0.25–0.55)
Central Asia	709.06 (568.33–897.73)	3.56 (2.88–4.46)	1305.76 (1036.63–1688.80)	3.18 (2.53–4.11)	−0.32^*^ (−0.56 to −0.08)
Central Europe	1830.05 (1531.71–2238.67)	3.45 (2.90–4.21)	1665.54 (1400.82–2022.99)	2.76 (2.31–3.38)	−0.76^*^ (−0.96 to −0.55)
Central Latin America	2806.45 (2264.21–3502.76)	5.66 (4.61–7.01)	6729.96 (5355.04–8631.73)	5.99 (4.76–7.68)	0.18^*^ (0.12–0.24)
Central Sub-Saharan Africa	587.82 (425.61–783.85)	4.32 (3.16–5.70)	1695.35 (1207.21–2247.35)	4.23 (3.03–5.56)	−0.06^*^ (−0.1 to −0.01)
East Asia	30863.96 (24827.40–36966.16)	6.98 (5.65–8.32)	53088.71 (40969.64–69440.99)	6.94 (5.35–9.09)	−0.03 (−0.12 to 0.06)
Eastern Europe	2780.27 (2174.23–3630.14)	3.21 (2.54–4.15)	3485.79 (2762.41–4449.31)	3.33 (2.63–4.27)	0.17 (−0.1 to 0.44)
Eastern Sub-Saharan Africa	2004.00 (1528.84–2572.01)	4.32 (3.33–5.49)	4884.78 (3607.27–6360.94)	3.93 (2.93–5.08)	−0.30^*^ (−0.33 to −0.28)
High-income Asia Pacific	2815.84 (2305.85–3494.68)	3.27 (2.66–4.07)	3057.85 (2475.20–3834.93)	3.17 (2.52–4.05)	−0.09 (−0.22 to 0.05)
High-income North America	4876.80 (3954.68–6055.67)	3.94 (3.22–4.86)	6194.88 (5499.46–7018.00)	3.86 (3.42–4.38)	−0.06 (−0.18 to 0.06)
North Africa and Middle East	5236.52 (4146.23–6549.10)	5.53 (4.41–6.86)	14679.28 (11756.62–18457.62)	5.24 (4.21–6.58)	−0.17^*^ (−0.23 to −0.11)
Oceania	85.18 (61.00–113.95)	4.31 (3.11–5.74)	207.77 (152.70–282.99)	4.03 (2.97–5.47)	−0.21^*^ (−0.31 to −0.11)
South Asia	15852.03 (12190.14–20459.55)	4.50 (3.49–5.78)	38478.66 (29497.95–49444.15)	5.00 (3.85–6.41)	0.33^*^ (0.22–0.43)
Southeast Asia	7996.39 (6559.30–9744.78)	5.49 (4.55–6.64)	18458.46 (15204.24–22509.45)	5.62 (4.63–6.87)	0.10^*^ (0.04–0.16)
Southern Latin America	566.62 (467.43–696.68)	2.95 (2.44–3.62)	878.49 (683.01–1140.17)	2.82 (2.19–3.67)	−0.13 (−0.28 to 0.01)
Southern Sub-Saharan Africa	713.90 (578.11–879.45)	4.55 (3.71–5.58)	1736.58 (1412.04–2113.61)	5.25 (4.29–6.36)	0.39^*^ (0.21–0.58)
Tropical Latin America	2281.62 (1768.11–2932.37)	4.47 (3.51–5.68)	5525.19 (4288.49–7151.62)	5.03 (3.90–6.52)	0.41^*^ (0.35–0.47)
Western Europe	6379.26 (4898.33–8507.79)	3.74 (2.87–5.00)	7347.13 (5463.60–10020.76)	3.65 (2.68–5.04)	−0.1 (−0.26 to 0.07)
Western Sub-Saharan Africa	2583.06 (1971.96–3291.56)	5.06 (3.90–6.40)	7339.33 (5128.15–10761.99)	5.27 (3.71–7.69)	0.14^*^ (0.06–0.22)

UI, uncertainty interval; CI, confidence interval; AAPC, average annual percent of change; DALY, disability-adjusted life years; ASDR, age-standardized DALYs rate; SDI, socio-demographic index. *indicates *p* < 0.05.

**Figure 1:**
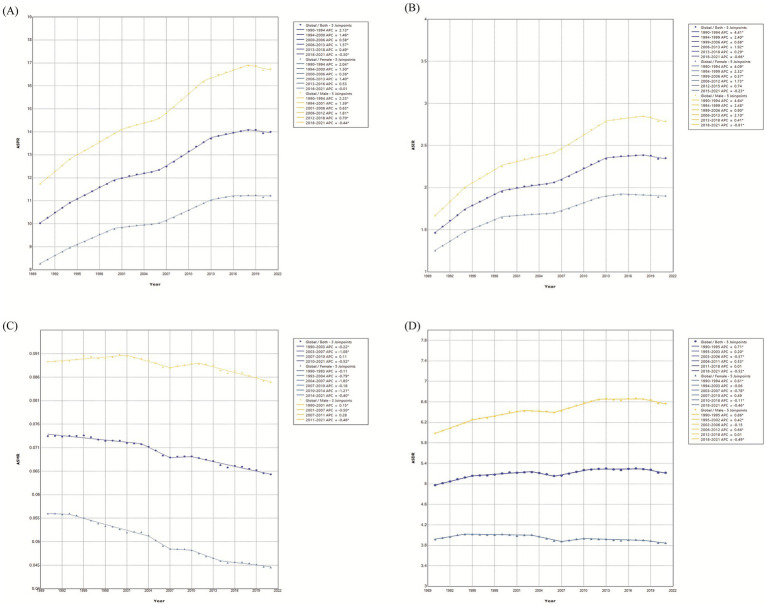
Temporal trends of prevalence, incidence, mortality, and DALYs from 1990 to 2021. **(A)** Prevalence. **(B)** Incidence. **(C)** Mortality. **(D)** DALYs. APC, annual percent of change; AAPC, average annual percent of change. ^*^Indicates *p* < 0.05.

An in-depth joinpoint analysis presented varied trends in prevalence, incidence, mortality, and DALYs during the periods of 1990 to 2021. ASPR presented the continuous increasing in 1990–1994, 1994–2000, 2000–2006, 2006–2013, and 2013–2018, with the APCs of 2.13, 1.46, 0.58, 1.58, 1.57, and 0.49, respectively, but showed a downward trend from 2018 to 2021 with an APC of −0.30 (Figure 1A). Similar with the ASPR, ASIR presented the significant decrease trend during 2018–2021, with the APC of −0.66 (Figure 1B). ASMR showed a marked decline in 1990–2003, 2003–2007, and 2010–2021, with the APCs of −0.22, −1.08, and −0.52, and keep stable from 2007 to 2010 (Figure 1C). ASDR exhibited a fluctuating trend, with an upward trend in three periods (1990–1995, 1995–2003, and 2006–2011) and a downward trend in two periods (2003–2006 and 2018–2021) (Figure 1D).

### Global trends by sex

The discernible rises in ASPR, ASIR, and ASDR, and a decline in ASMR were observed over this three-decades period for both sexes. While both males and females exhibited similar change patterns, males consistently keep higher ASRs in all measures. When focusing the absolute numbers of all measures, males were always higher than females in 1990 and 2021 (Tables 1–4). In prevalence, the results indicated a significant downward trends for males occurred in 2018–2021, and occurred in 2016–2021 for females, with the APCs of −0.44 and −0.01, respectively (Figure 1A). ASIR also presented a decrease in males during 2018–2021 (APC = −0.81) and in females during 2015–2021 (APC = −0.23) (Figure 1B). In mortality, male showed a significant decline trend in 2011–2021 (APC = −0.46). The females keep a continuous downward trend from 1990 to 2021 (Figure 1C). Moreover, DALYs trend showed the difference between males (AAPC = 0.30) and females (AAPC = −0.07) (Figure 1D).

### Global trends by age group

**Figure 2:**
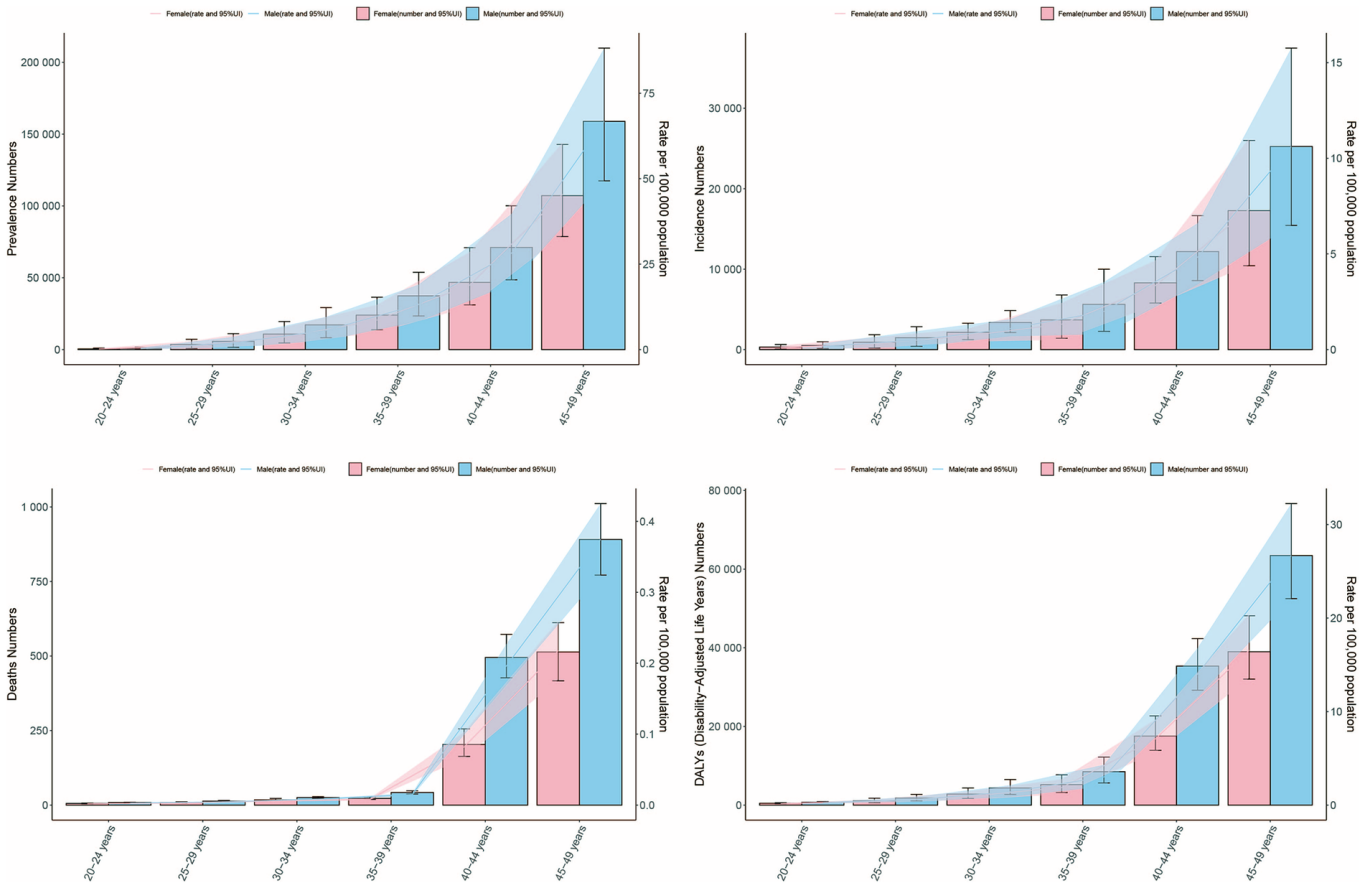
Absolute number and age-standardized rates of prevalence, incidence, mortality, and DALYs for EOPD stratified by sex and age group in 2021. **(A)** Prevalence. **(B)** Incidence. **(C)** Mortality. **(D)** DALYs. Bar charts represent numbers; lines represent age-standardized rates. Red represents female, and blue represents male.

The absolute number and ASR in prevalence and incidence increased with the age, reaching the highest in 45–49 age groups, and males consistently outpaced the females across all age groups (Figures 2A,B). In mortality, ASMR surged most significantly for both sexes at 40–44 age group and 45–49 group (Figure 2C). As for DALYs, the trend mirrored the age progression, reaching the zenith in the 45–49 age group (Figure 2D). The highest proportion of age groups in prevalence, incidence, mortality, and DALYs were shown in 45–49 age group, and the low proportion occurred in 20–24 age group (Supplementary Figure S1).

### SDI region trends

In 2021, the highest absolute numbers of prevalence, incidence, mortality, and DALYs of EOPD were observed in the middle SDI (Tables 1–4). The cases in middle SDI region accounted for approximately 38.9% of the total cases in 2021. The highest ASPR and ASDR were shown in middle SDI regions, the highest ASIR in high-middle SDI region, and the lowest ASMR in high SDI regions. The ASPR and ASIR of EOPD in all SDI regions presented the significant increase trends in some degree, with the high-middle SDI being the most at the 1.68 (95% CI: 1.61–1.76) and 2.37 (95% CI: 2.26–2.48), respectively. In mortality, low SDI, middle SDI, and high-middle SDI regions experienced the substantial decline of ASMR with the AAPCs of −0.24 (95% CI: −0.35 to −0.13), −0.78 (95% CI: −0.95 to −0.61), and −0.92 (95% CI: −1.19 to −0.65). In DALYs, low-middle and high SDI regions presented the significant upward trends of ASDR with the AAPCs of 0.25 (95% CI: 0.20–0.31) and 0.13 (95% CI: 0.03–0.23). The trends of ASPR, ASIR, ASMR, and ASDR among five SDI regions from 1990 to 2021 were shown in Supplementary Figure S2.

### Regional trends

In 2021, the largest absolute number of EOPD occurred in East Asia, followed by South Asia and Southeast Asia, at 166105.93 (95% UI: 115257.24–233537.65), 107011.67 (95% UI: 69536.23–153542.11), and 33111.56 (95% UI: 21639.66–48543.10), respectively. The highest ASPR in 2021 was observed in Andean Latin America at 30.22 (95% UI: 20.24–43.61) per 100,000 populations. East Asia showed a substantial rise in ASPR with an AAPC of 2.55 (95% CI: 2.45–2.66) (Table 1). As for incidence, Andean Latin America had the highest ASIR [5.10 (95% UI: 3.09–7.51)] per 100,000 populations. From 1990 to 2021, East Asia displayed a largest increase of ASIR at an AAPC of 3.39 (95% CI: 3.32–3.47), while high-income North America showed a significant decline at an AAPC of −0.38 (95% CI: −0.43 to −0.34) (Table 2). In terms of mortality, the majority of regions experienced a decline in ASMR in these three decades, with the largest decline in East Asia at an AAPC of −1.36 (95% CI: −1.46 to −1.25) (Table 3). Furthermore, the impact of EOPD-related DALYs displayed varied trends in 21 GBD regions. Australasia showed the most substantial increase in ASDR with an AAPC of 0.60 (95% CI: 0.30–0.91). In contrast, Central Europe showed the most decline trend with an AAPC of −0.76 (95% CI: −0.96 to −0.55) (Table 4).

### National trends

**Figure 3:**
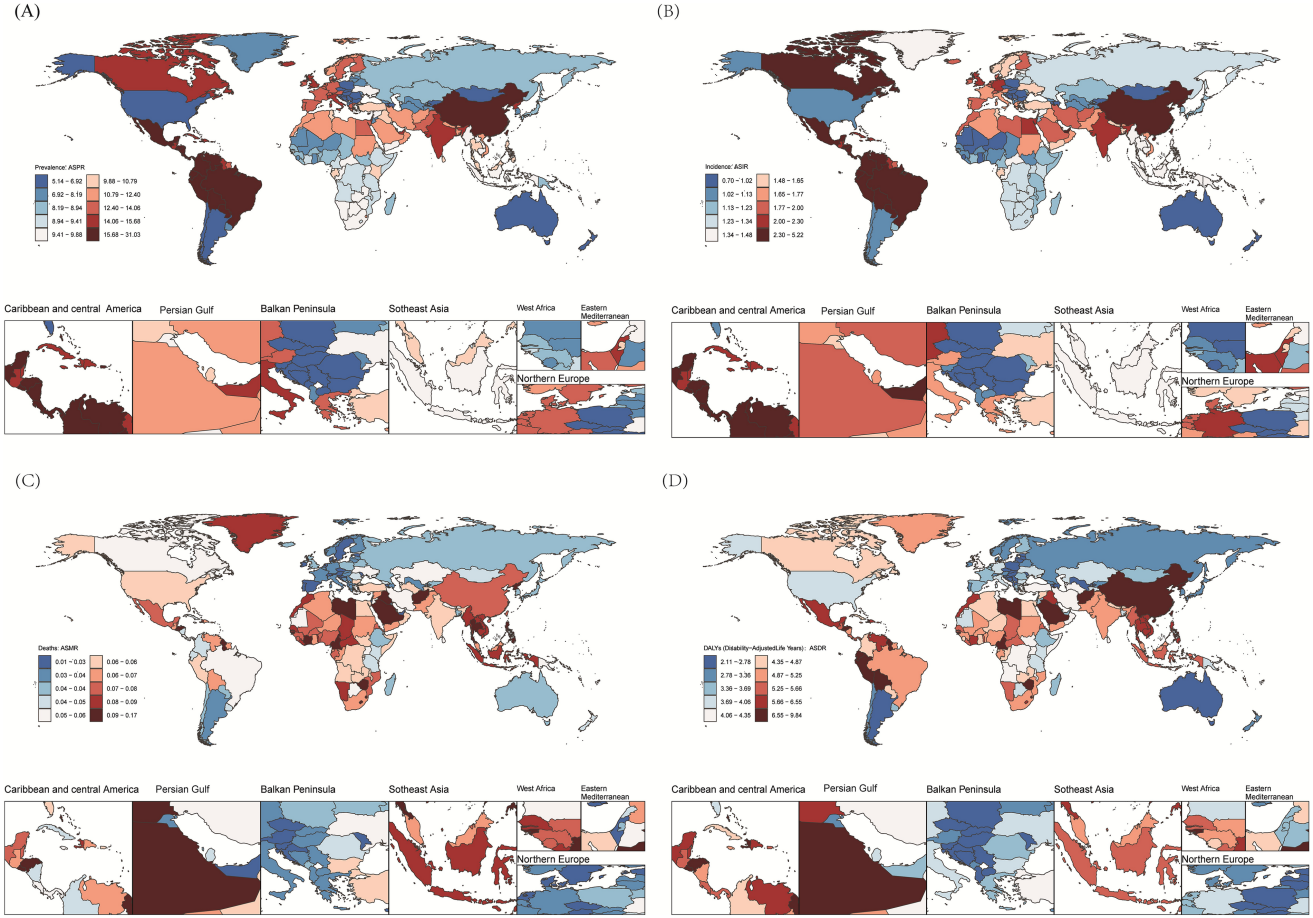
Global age-standardized rates of prevalence, incidence, mortality, and DALYs for EOPD across 204 countries and territories in 2021. **(A)** Prevalence. **(B)** Incidence. **(C)** Mortality. **(D)** DALYs.

At the national level, China had the largest number of prevalent cases in 2021 [162648.66 (95% UI: 112605.30–229007.80)]. The ASPR of EOPD ranged from 5.19 to 30.72 per 100,000 populations across 204 countries and territories. Peru, Bolivia, and Ecuador had the highest ASPR (Figure 3A; Supplementary Figure S3; Supplementary Table S1). A total of 180 countries experienced significant increases, with China being the most notable with an AAPC of 2.59 (95% CI: 2.50–2.68). In contrast, 15 countries occurred the decline trends, including Poland with an AAPC of −1.60, United States of America with an AAPC of −1.52, and Republic of Moldova with an AAPC of −0.29 (Supplementary Table S1). As for incidence, 176 countries underwent the significant increases, with China at the forefront with an AAPC of 3.45 (Supplementary Table S2; Figure 3B; Supplementary Figure S3). Mortality rates were varied across 204 countries and territories, with the Kuwait, United Arab Emirates, and Slovenia recording the most decline with the AAPCs of −3.60, −3.46, −2.46. Notably, 147 countries presented the significant decline trend in ASMR of EOPD (Supplementary Table S3; Figure 3C; Supplementary Figure S3). For DALYs, the countries with the largest decreases in ASDR of EOPD were Kuwait, Maldives, and Slovenia, with the AAPCs of −2.10, −1.51, and −1.41, while the Lesotho, Zimbabwe, and Libya presented the largest upward trend with the AAPCs of 1.67, 1.52, and 1.39, respectively (Supplementary Table S4; Figure 3D; Supplementary Figure S3).

### Correlations of ASR with SDI

**Figure 4:**
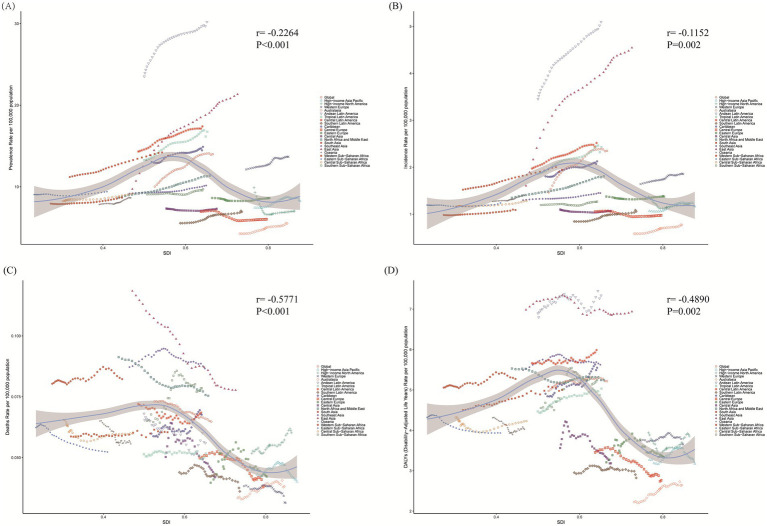
The associations between age-standardized rates and SDI values for EOPD across 21 regions from 1990 to 2021. The black line presented the expected values based on the SDI and age-standardized rates in all locations, and each point represented the observed age-standardized rate for each region from 1990 to 2021. **(A)** Prevalence. **(B)** Incidence. **(C)** Mortality. **(D)** DALYs. SDI, socio-demographic index.

In 21 GBD regions, there were significant correlation between all metrics and SDI. ASR in prevalence, incidence, mortality, and DALYs all displayed negative correlations with the SDI, being weak negative in ASPR (*r* = −0.2264, *p* < 0.001) and ASIR (*r* = −0.1152, *p* = 0.002), and moderate negative in ASMR (*r* = −0.5771, *p* < 0.001) and ASDR (*r* = −0.4814, *p* < 0.001) (Figure 4). In addition, a broader analysis was conducted in 204 countries and territories. In prevalence and incidence, the weak positive correlations were shown in ASPR (*r* = 0.1243, *p* = 0.076) and ASIR (*r* = 0.2075, *p* = 0.003) across 204 countries and territories, while ASPR’s correlation did not reach the statistical significance. A moderate negative correlation was observed between ASMR and SDI in EOPD across 204 countries and territories (*r* = −0.6029, *p* < 0.001). This trend continued with ASDR, which also moderately negatively correlated with SDI in EOPD (*r* = −4,890, *p* < 0.001) (Supplementary Figure S4).

### Health inequality analysis

**Figure 5:**
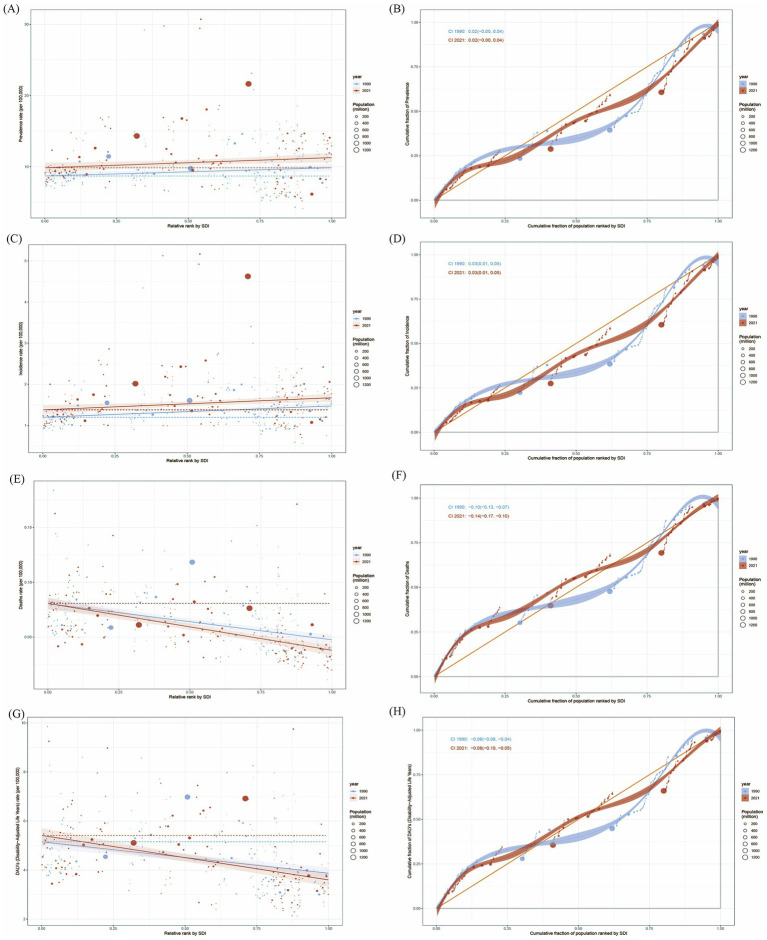
Health inequality of age-standardized rates for EOPD from 1990 to 2021. **(A)** Health inequality regression curves for ASPR of EOPD. **(B)** Concentration curves of ASPR for EOPD. **(C)** Health inequality regression curves for ASIR of EOPD. **(D)** Concentration curves of ASIR for EOPD. **(E)** Health inequality regression curves for ASMR of EOPD. **(F)** Concentration curves of ASMR for EOPD. **(G)** Health inequality regression curves for ASDR of EOPD. **(H)** Concentration curves of ASDR for EOPD. ASPR, age-standardized prevalence rate; ASIR, age-standardized incidence rate; ASMR, age-standardized death rate; ASDR, age-standardized DALYs rate.

The analysis displayed the significant absolute and relative inequalities in all measures due to EOPD. In SII, the gap in ASPR and ASIR between the highest SDI and the lowest SDI countries and territories, increased from 1.17 (95% CI: 0.13–2.22) and 0.27 (95% CI: 0.13–0.42) in 1990 to 1.42 (95% CI: 0.00–2.85) and 0.29 (95% CI: 0.09–0.50) in 2021, respectively, and the CIX of prevalence and incidence did not change significantly between 1990 and 2021 (Figures 5A–D). In mortality, the SII was −0.03 (95% CI: −0.04 to −0.02) in 1990 and −0.04 (95% CI: −0.05 to −0.03) in 2021, and the CIX of mortality changed from −0.10 (95% CI: −0.13 to −0.07) in 1990 to −0.14 (95% CI: −0.17 to −0.10) (Figures 5E,F). As for DALYs, the SII changed from −1.28 (95% CI: −1.71 to −0.85) in 1990 to −1.82 (95% CI: −2.31 to −1.32) in 2021. The CIX also changed from −0.06 (95% CI: −0.08 to −0.04) in 1990 to −0.08 (95% CI: −0.10 to −0.05) in 2021 (Figures 5G,H). All these results demonstrated an increase in absolute health inequalities in all measures of EOPD, meanwhile, the inequality had widened in mortality and DALYs.

### Frontier analysis

**Figure 6:**
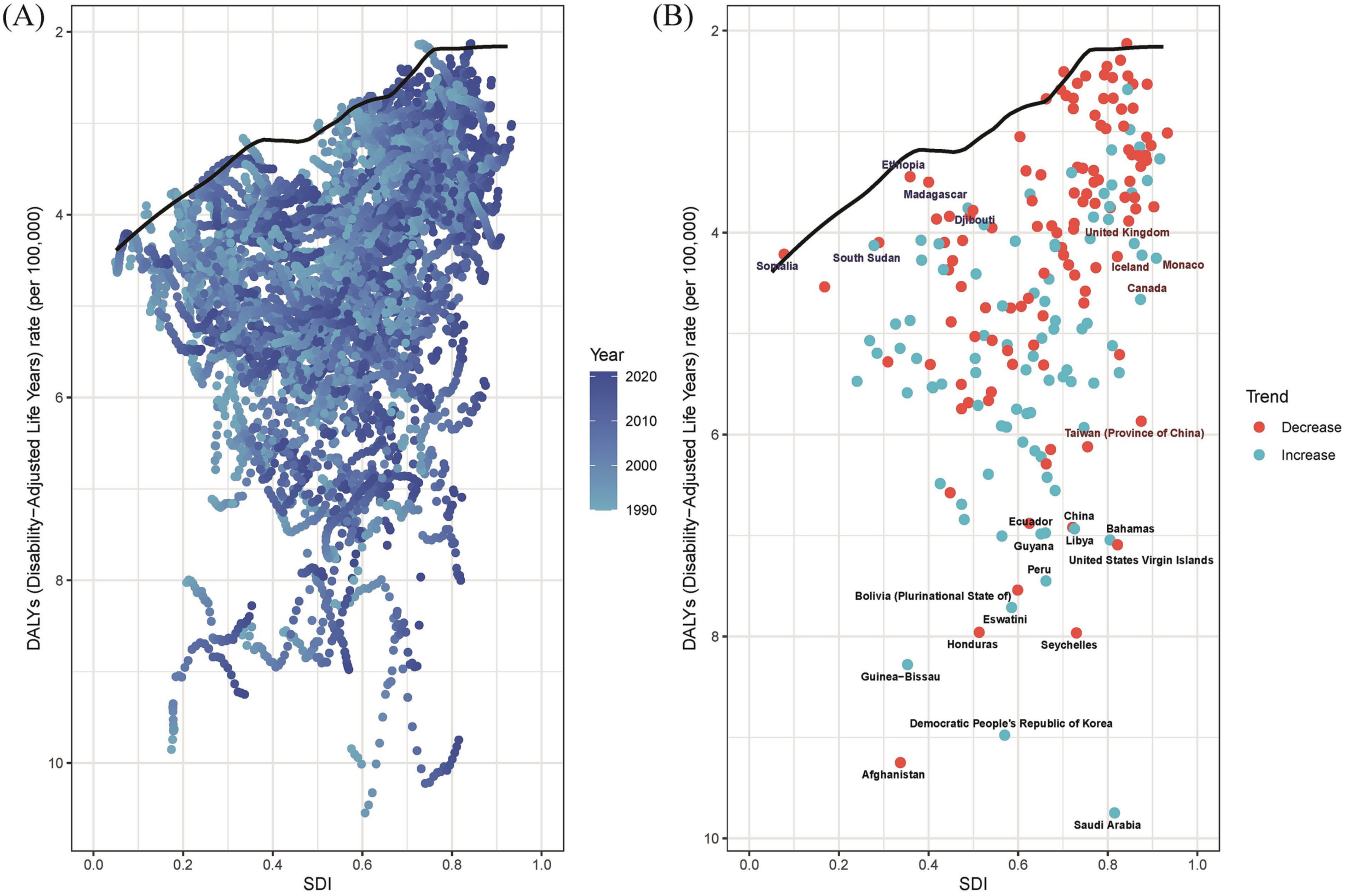
Frontier analysis of ASDR in EOPD based on SDI from 1990 to 2021. The black solid lines represented the lower limits of ASDR achievable at different SDI values, with points representing different countries and territories, respectively. The color scale ranged from light blue (1990) to dark blue (2021). The five countries with the smallest effective differences among low SDI countries and territories were labeled in blue font. The five countries and territories with the largest effective differences among high SDI countries and territories were labeled in red font. The 15 countries and territories with the largest effective differences globally were labeled in black font. **(A)** and **(B)**: Frontier analysis for ASDR.

The frontier analysis was conducted to explore the potential improvement space of the ASDR for EOPD. The black solid line delineated the countries and territories with the lowest ASDR given their SDI. Distance from the frontier was defined as “effective difference,” representing the gap between observed ASR and potentially achievable ASR. As the SDI value rose from 0 to 1.0, there was an overall decline in ASDR for EOPD (Supplementary Figure S4). The top 15 countries and territories with the highest effective differences in EOPD were Saudi Arabia, Afghanistan, Democratic People’s Republic of Korea, Guinea-Bissau, Honduras, Eswatini, Seychelles, Bolivia, Peru, United States Virgin Islands, Guyana, Libya, Bahamas, Ecuador, and China. In addition, the five countries with the lowest ASDR and minimal effective differences were Somalia, South Sudan, Ethiopia, Madagascar, and Djibouti. Conversely, the five countries with high SDI and relatively high effective difference were Taiwan (Province of China), Canada, Iceland, Monaco, and United Kingdom (Figure 6). Notably, as socio-demographic development progressed, the effective difference in some countries and territories tends to increased, suggesting that countries or regions with a higher SDI possess greater potential for improvement in health outcomes.

### Future trends of EOPD from 2021 to 2030

**Figure 7:**
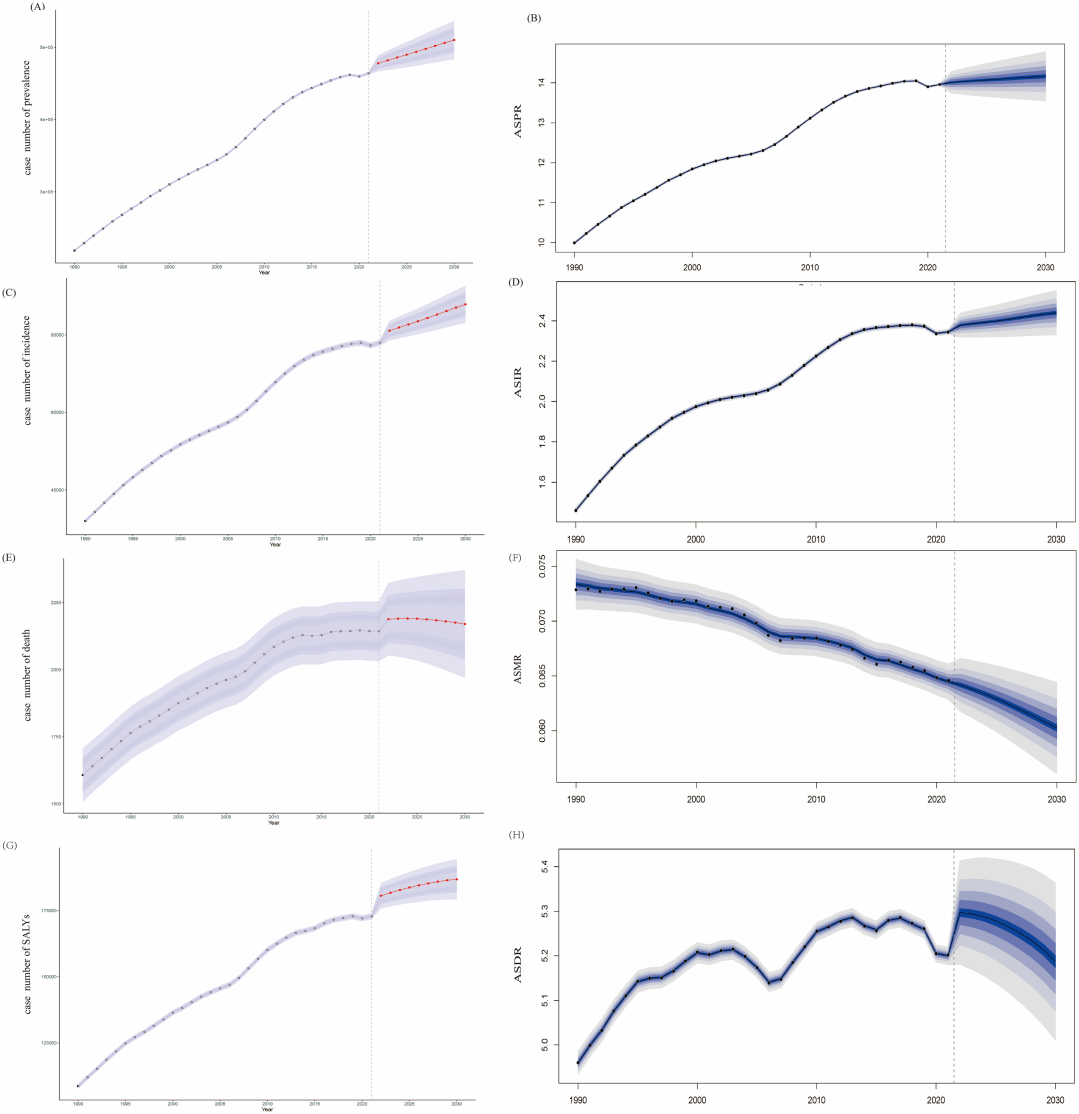
The forecasts of case numbers and age-standardized rates of prevalence, incidence, mortality, and DALYs for EOPD till to 2030. **(A,B)** Prevalence. **(C,D)** Incidence. **(E,F)** Mortality. **(G,H)** DALYs. The blue regions represented the upper and lower limits of 95% uncertainty interval (UI).

Furthermore, we projected the future trends of EOPD in the next decade. The number of prevalence and incidence will continue to increase, reaching 510125.56 (95% CI: 483563.98–536688.14) and 87870.76 (95% CI: 83097.89–92643.62) (Figures 7A,C), and ASPR and ASIR will slightly increase to 14.16 per 100,000 populations (95% CI: 13.43–14.90) and 2.44 per 100,000 populations (95% CI: 2.31–2.57) until 2030, respectively (Figures 7B,D). In mortality, the absolute number of death will slightly increase to 2170.05 (95% CI: 1968.78–2371.33) by 2030 (Figure 7E), but ASMR will drop to 0.060 per 100,000 populations (95% UI: 0.05–0.06) (Figure 7F). Simultaneously, the ASDR continuously increased to 6.67 (95% CI: 6.50–6.85) in 2022, and then keep the decline trend to 2030 by 6.51 (95% CI: 6.23–6.78) across the general population (Figure 7H), but the absolute number keep increasing till to 2030 (Figure 7G).

## Discussion

Based on our analysis, it was clear that the global prevalence cases of EOPD increased from 190486.50 in 1990 to 483872.47 in 2021, a total increase of 2.54 times. The prevalence mainly reflects the increased incidence and duration of disease. Previous studies indicated that the industrialization, urbanization, and socioeconomic development can predispose people to PD (16–18). Moreover, the developments in medical technology have improved diagnostic capabilities of EOPD. Also, the enhancement of medical care has ensured prognosis for EOPD patients. Therefore, the increased ASPR of EOPD may be due to the increase in ASIR and the decrease in ASMR in the last three decades, which suggests that the burden of EOPD had not decreased. Despite the significant burden posed by EOPD, we noticed that EOPD presented slight decline trends in prevalence and incidence from 2018 to 2021. Currently, continuous optimization of clinical guidelines has made the potential impact on reducing this burden, including “Guideline for Treatment of Early Parkinson’s disease” issued by American Academy of Neurology, “Parkinson’s disease in adults: diagnosis and management” issued by National Institute for Health and Care Excellence, “Guidelines for Therapeutic Management of Parkinson’s disease in China,” and so on (19–21). The innovative use of artificial intelligence and machine learning models have been implicated in diagnosis, prevention and treatment of EOPD (22). Moreover, the widespread adoption of innovative medications in EOPD management appeared to have influence on the decline trend between 2018 and 2021. All measures are anticipated to contribute positively to alleviate the EOPD burden. However, there are some special reasons, such as the outbreak of COVID-19 in 2019 and quarantine policy in some countries, which could affect the results of the survey. In order to reduce the COVID-19 impacts and data biases, The GBD platform integrated and optimized data from multiple resources, improved modeling methods, and corrected data biases. Even so, we should need more extensive researches and analysis for the decline reasons.

We revealed an absolute predominance of males in EOPD, but it is worth noting that the AAPCs in males were all significantly higher than those in females. Amounting evidence has demonstrated that sex is an important factor in the development of EOPD. The differences in genetic factors, sex hormones, brain development/function, epidemiological and clinical profiles, and environmental exposures/life style factors are potential explanations for this difference (23–25). First, men are more frequently exposed to environmental heavy metals and pesticides linked to Parkinson’s disease (26). Second, strong evidence supports the hypothesis that estrogens contribute to the sex-related prevalence of PD in adults, and estrogens may play a beneficial effect on neurodegeneration and reduce the disease risk (27). Third, genetically, Nordengen et al. (28) suggested that sex-specific gene differences associated with Parkinson’s disease could partly explain the increased incidence of the disease in the male population by combining large GWAS datasets from Parkinson’s disease and sex-specific traits with in-depth RNA sequencing data from human brains. Our results highlighted the need for sex-specific health interventions to address these disparities. As the second most common age-related neurodegenerative disease, the ASR in all measures of EOPD all increased with age. According to GBD 2016 data, PD incidence was primarily concentrated in the 70–74 age group and 75–79 age group (29). Reeve et al. (30) suggested that advancing age was an important risk factor in PD. As expected, the absolute cases of prevalence, incidence, death, and DALYs for EOPD all reached zenith in the age group of 45–49 years in 2021.

The results indicated significant geographical disparities in EOPD when examining the 21 GBD regions and 204 countries and territories. The epidemiology data indicated that genetic predisposition, geographical characteristics, environmental influences, economic development, and population demographics are associated with the risk of PD (31). Moreover, a growing evidence demonstrated that increased risk of PD has been associated with exposure to pesticides, consumption of dairy products, hypertension, alcohol intake, and traumatic brain injury, whereas smoking, coffee consumption, black tea drinking, higher serum urate concentrations, high sleep quality, low-to-moderate sitting time, higher plasma vitamin D, and physical activity could alleviate the risk of PD (32). Our analysis showed that the highest absolute number of prevalence, and incidence occurred in East Asia due to the largest population base, accounting for approximately one-third of global data. In terms of AAPCs of ASPR and ASIR, East Asia, including mainly Chia, Japan, and Korea, also experienced the most substantial increases over the last three decades. Epidemiology studies demonstrated that PD has a strong genetic component, especially in EOPD. Koros et al. (33) showed that *GBA1* mutations (a hereditary risk factor) appeared to play an important role in patient groups with an East Asian background. In China, one of major countries in East Asia, undergoing the rapid industrialization and urbanization since 1990, the ASPR and ASIR of EOPD more than doubled between 1990 and 2021, the largest increase worldwide. Environmental exposure (including pesticide, herbicide, heavy metals and air pollution) may be the risk factors for PD (34). China, as one of the world’s largest pesticide and herbicides users, therefore, has more potential PD risk. Similar with our analysis, a recent report suggested that China had the highest ASPR and ASIR of PD among the G20 countries (9). However, high-income North America, mainly including USA and Canada, showed the substantial decline trends in prevalence and incidence, but a largest increased trend in mortality. The economic development and healthcare access are key contributors in the regional differences. The individuals in developed countries and territories are more likely to access quality healthcare, safe living environment, and nutritious diets, thereby improving health outcomes. The decreasing incidence and prevalence of EOPD could prove an effectiveness of preventive strategies. There were some interpretations that could promote the better understandings of the increase trend in EOPD mortality in high-income North America. The PD-related complications, such as infections, falls, cardiovascular disease, and treatment-related motor complications, can directly lead to death (35). A large sample cohort study showed that EOPD patients with dyskinesia, severe non-motor burden, non-smoking habit were more likely to develop suicide due to more frequent depression under occupational and lifestyle challenges (36). In addition, changes in calculation methods and availability of registration practices might have led to accurate estimation of deaths since 1990 (37). Therefore, despite a global decline in the mortality of EOPD, certain countries or regions continued to face significant challenges. All results suggested that EOPD imposed a more substantial burden on healthcare systems and the economy without effective global countermeasures.

Our analysis found that the correlations of ASPR/ASIR and SDI displayed different trends at region and nation levels. These results suggested that EOPD was not caused by single factor, but a multi-factor, multi-system, and multi-stage dynamic process. The relationship between ASMR and ASDR and SDI were strikingly negative, underscoring the beneficial impact of high SDI on reducing the disease burden of EOPD. These negative correlations were presumably associated with the access of better medical services in the high SDI regions, which allow better health awareness, earlier diagnosis, and timely treatment of EOPD. Nevertheless, our analysis indicated that socio-demographic progress is essential for improving EOPD outcomes, highlighting the importance of enhancing SDI as a core goal in health policy and management. On the whole, we should improve healthcare accessibility and early intervention in low-income countries, and optimize long-term management and investment in research in high-income countries. For some aging countries, we should strengthen care systems and policy support. In regions with high exposure to environmental risks, we should strengthen source prevention, control and health education.

The analysis of cross-country health inequalities in EOPD burden with SDI could clarify the pattern of burden distribution and identify the countries where EOPD prevention and control should be improved. Our analysis suggested that the gaps in the prevalence and incidence for EOPD between high SDI- and low SDI countries increased between 1990 and 2021, but the CIX were stable, reflecting the EOPD burden was higher among high SDI countries, and this inequality had not changed over time. As indicted by SII and CIX in mortality and DALYs, the absolute inequality and relative inequality both increased, implying that the EOPD burden was consistently higher in low SDI countries, and the inequality had worsened over time. The inequality of EOPD burden concentrating in low SDI countries may be mainly attributed to the limited health awareness, inefficiency of health resource and poor medical treatment. Through standardized data integration, stratified prevention and control, transnational collaboration, policy promotion and other multidimensional measures, the gap can be systematically narrowed to achieve fairness and effectiveness of global PD prevention and control. At the same time, the data analysis tools of GBD should be continuously used to dynamically assess the effectiveness of interventions and optimize the direction of strategies.

In addition, frontier analysis was performed to analyze the potential improvement space for EOPD in 204 countries and territories, aiming to identify the theoretical EOPD burden that a country and territory could achieve based on its SDI value. The frontier analysis could provide several benefits to health providers and policy makers, and help them understand public health policy efficiency and effectiveness. The analysis identified 15 countries with the most significant potential for actual improvement in EOPD burden. Despite their varied socio-demographic backgrounds, all grapple with increased DALYs rates, distancing them from the optimal frontier line. However, Somalia, South Sudan, Ethiopia, Madagascar, and Djibouti appeared as frontier countries with low SDI, showing outstanding performance in improving EOPD burden. The possible explanation was that more international programs and fellowships have become available for researchers in African Over the past decades (38). Conversely, five countries and territories with high SDI did not perform as expected in controlling EOPD burden. Although these countries have better health resources and medical care, they still need to improve long-term follow-up and multidisciplinary collaboration to ensure ongoing medical support and improved life quality in EOPD patients. All these disparities reflected the complex nature of healthcare outcomes, suggesting that not only SDI was a significant factor, but also other factors, including genetic, environmental, and lifestyle, play crucial roles in shaping EOPD trends.

Future projection based on the BAPC models showed that the total number of EOPD cases was expected to keep rising to nearly 510,125 by 2030. As for future mortality trends, BAPC model provide optimistic projections, with the ASMR will keep decreasing trends in the next decade, likely due to ongoing advancement in diagnosis and treatment, and improvements in healthcare system. Regarding DALYs trend, BAPC model suggested that ASDR of EOPD will peak in 2022, followed by a significant decline till to 2030. These projections underscored the urgency for feasible and sound health policies focusing on reducing the prevalence and incidence and improving long-term outcomes for EOPD.

However, our study undoubtedly had some limitations. First, incomplete and inconsistent data across different countries and territories could affect the accuracy of analysis. Inadequate EOPD registries in some underdeveloped countries could lead to underestimation of EOPD cases, as well as misdiagnosis or missed diagnosis due to poor medical conditions. Second, methodological differences (case ascertainment, diagnostic criteria, or age distributions of the study populations) might result in varied estimates and underestimate the true burden of EOPD (39). Third, due to the lack of strong predictors for the occurrence of EOPD, some variations could cause all sources of measurement bias, which require more complex survey procedures to establish a diagnosis. Forth, the ICD-9/10 code in the GBD database does not by itself directly distinguish between EOPD and LOPD, although it can be analyzed indirectly through age stratification, genetic data supplementation, and multiple-source data verification. Finally, although GBD 2021 have taken into account the more commonly known comorbidities, it is intractable to compute accurately it for multiple diseases and their sequelae estimated in GBD 2021.

## Conclusion

Although the mortality rate had consistently declined, EOPD continued to be a major public health burden due to increasing trends of prevalence, incidence, and DALYs in the past three decades, especially for men. Countries and territories with low SDI shouldered disproportionately high EOPD burden, and the SDI-related health inequalities across countries and territories exacerbated over time. Meanwhile, the analysis results call for more health resource to be allocated in low SDI countries and territories, and to prevent EOPD in high SDI countries and territories. Targeted interventions and nuanced measures should be further considered to improve individualized healthcare system and tackle with different health needs in each country and territory.

## Data Availability

The raw data supporting the conclusions of this article will be made available by the authors, without undue reservation.

## Footnotes

^1^ https://vizhub.healthdata.org/gbd-results/

^2^ https://ghdx.healthdata.org/gbd-2021/sources

## Supplementary material

SUPPLEMENTARY FIGURE S1 Proportions of case numbers of different age groups for EOPD across 21 GBD regions. **(A)** Prevalence. **(B)** Incidence. **(C)** Mortality. **(D)** DALYs.

SUPPLEMENTARY FIGURE S2 Temporal trends of ASPR, ASIR, ASMR, and ASDR in five SDI regions from 1990 to 2021. **(A)** Prevalence. **(B)** Incidence. **(C)** Mortality. **(D)** DALYs. SDI, socio-demographic index; ASPR, age-standardized prevalence rate; ASIR, age-standardized incidence rate; ASMR, age-standardized death rate; ASDR, age-standardized DALYs rate.

SUPPLEMENTARY FIGURE S3 Global case number of prevalence, incidence, mortality, and DALYs for EOPD across 204 countries and territories in 2021. **(A)** Prevalence. **(B)** Incidence. **(C)** Mortality. **(D)** DALYs.

SUPPLEMENTARY FIGURE S4 The associations between age-standardized rates and SDI values for EOPD across 204 countries and territories from 1990 to 2021. The black line presented the expected values based on the SDI and age-standardized rates in all countries and territories, and each point represented the observed age-standardized rate for each countries and territories from 1990 to 2021. **(A)** Prevalence. **(B)** Incidence. **(C)** Mortality. **(D)** DALYs. SDI, socio-demographic index.
