# Supplementary figures and images for: Global trends of early-onset Parkinson’s disease from 1990 to 2021, and projections until to 2030: a systematic analysis of the global burden of disease study 2021
Source: Front Neurol. 2025 Aug 1;16:1589760. doi: 10.3389/fneur.2025.1589760 (PMC12354633; doi:10.3389/fneur.2025.1589760)

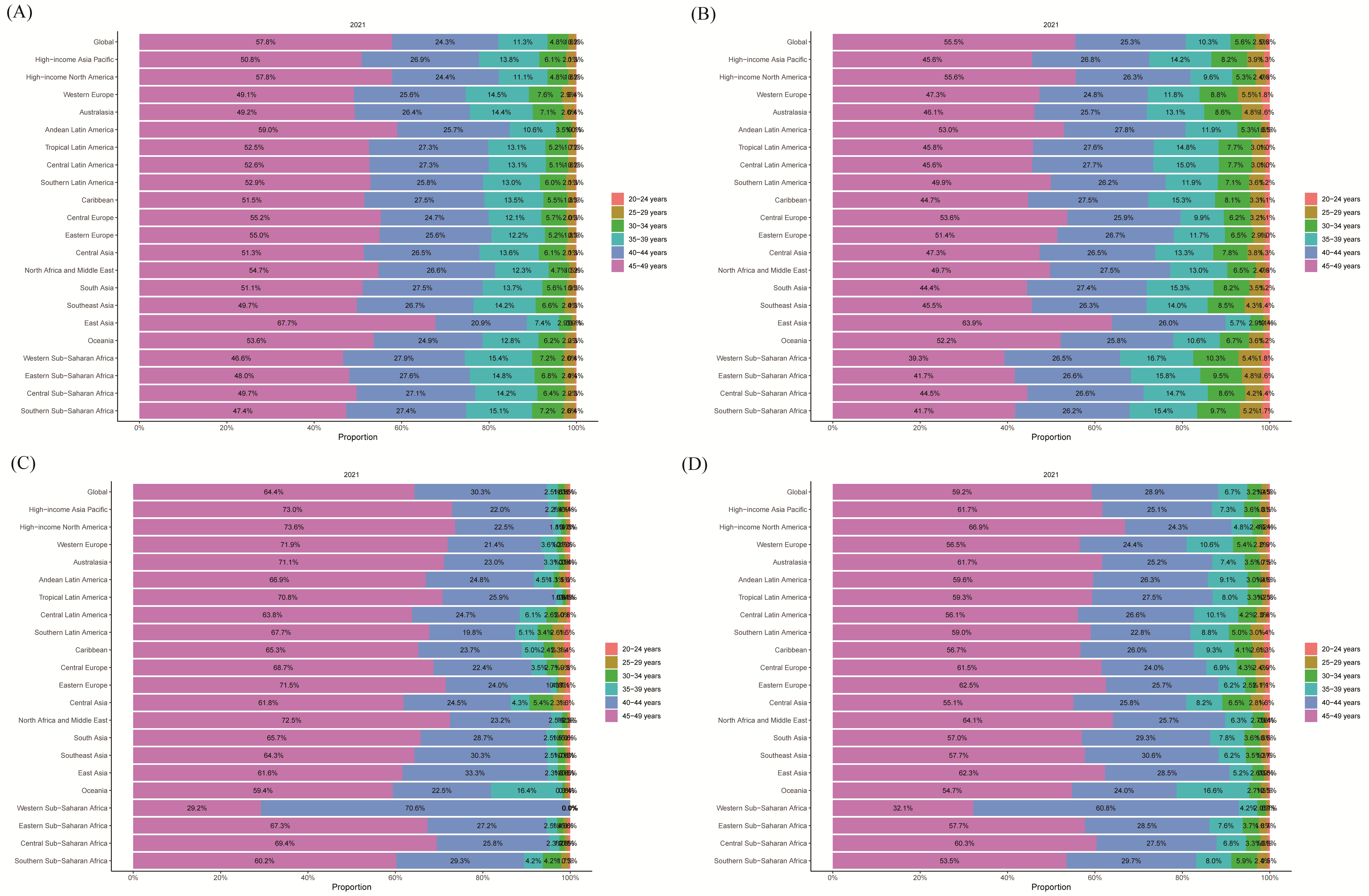

Supplement: SUPPLEMENTARY FIGURE S1 — Proportions of case numbers of different age groups for EOPD across 21 GBD regions. (A) Prevalence. (B) Incidence. (C) Mortality. (D) DALYs. [file Image_1.jpeg]

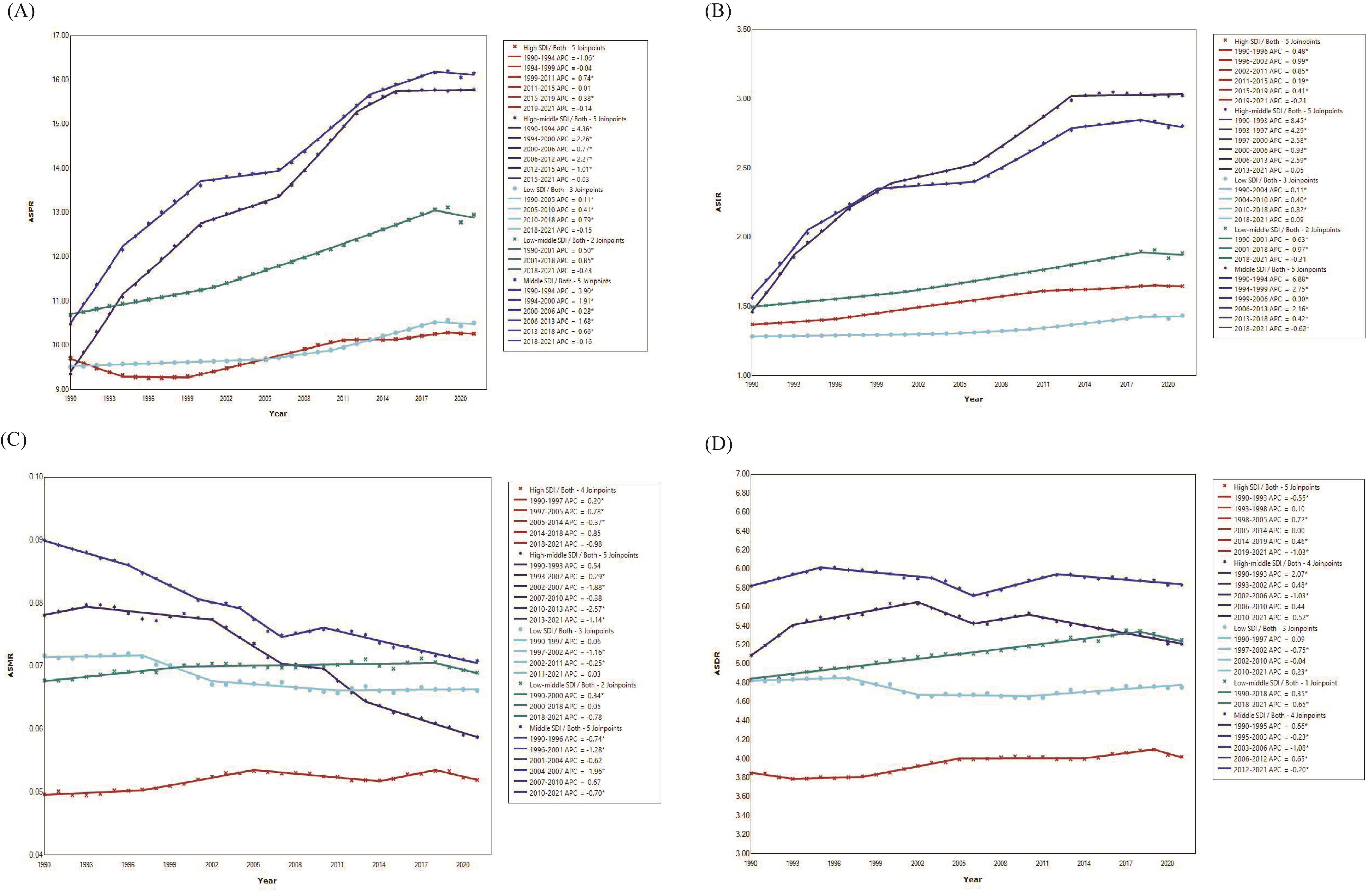

Supplement: SUPPLEMENTARY FIGURE S2 — Temporal trends of ASPR, ASIR, ASMR, and ASDR in five SDI regions from 1990 to 2021. (A) Prevalence. (B) Incidence. (C) Mortality. (D) DALYs. SDI, socio-demographic index; ASPR, age-standardized prevalence rate; ASIR, age-standardized incidence rate; ASMR, age-standardized death rate; ASDR, age-standardized DALYs rate. [file Image_2.jpeg]

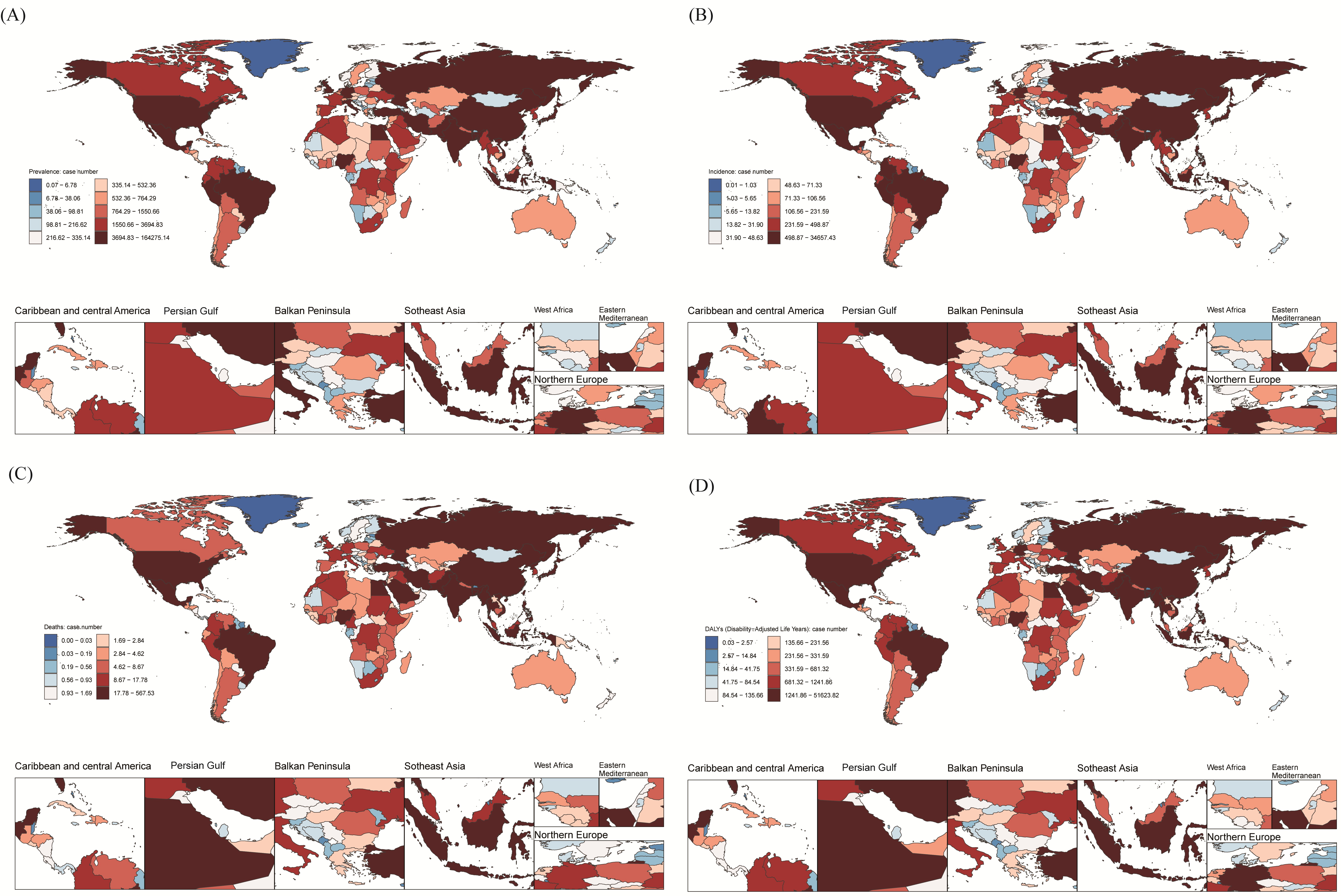

Supplement: SUPPLEMENTARY FIGURE S3 — Global case number of prevalence, incidence, mortality, and DALYs for EOPD across 204 countries and territories in 2021. (A) Prevalence. (B) Incidence. (C) Mortality. (D) DALYs. [file Image_3.jpeg]

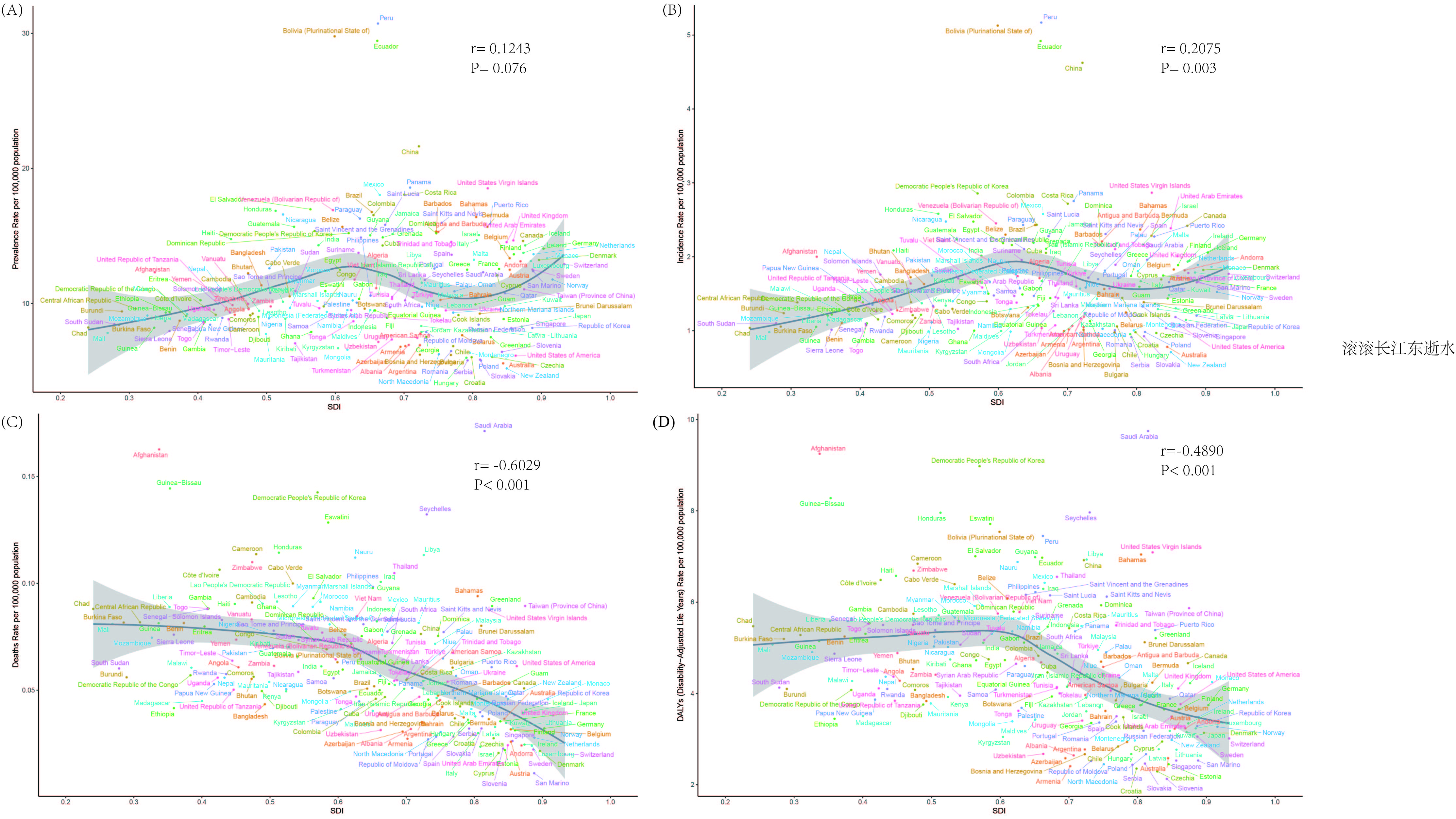

Supplement: SUPPLEMENTARY FIGURE S4 — The associations between age-standardized rates and SDI values for EOPD across 204 countries and territories from 1990 to 2021. The black line presented the expected values based on the SDI and age-standardized rates in all countries and territories, and each point represented the observed age-standardized rate for each countries and territories from 1990 to 2021. (A) Prevalence. (B) Incidence. (C) Mortality. (D) DALYs. SDI, socio-demographic index. [file Image_4.jpeg]
