# Supplementary material for: Global trends of early-onset Parkinson’s disease from 1990 to 2021, and projections until to 2030: a systematic analysis of the global burden of disease study 2021
Source: Front Neurol. 2025 Aug 1;16:1589760. doi: 10.3389/fneur.2025.1589760 (PMC12354633; doi:10.3389/fneur.2025.1589760)
Supplement: Supplementary file 5 [file Table_1.docx]

Table S1. The case number and age-standardized rate of prevalence among 204 countries and territories, with the AAPCs between 1990 and 2021

|  | 1990 | | 2021 | |  |
| --- | --- | --- | --- | --- | --- |
|  | Number (95%UI) | ASPR per 100,000 populations (95%UI) | Number (95%UI) | ASPR per 100,000 populations (95%UI) | AAPCs (95%CI) |
| Afghanistan | 293.74(190.94-426.10) | 10.20(6.42-15.20) | 1155.56(748.37-1694.70) | 12.31(7.95-18.07) | 0.61*(0.59-0.63) |
| Albania | 76.29(41.22-126.36) | 6.89(3.85-11.17) | 77.00(44.59-123.21) | 6.93(3.99-11.14) | 0.02(0-0.04) |
| Algeria | 566.06(327.43-895.40) | 8.84(5.31-13.62) | 2225.01(1345.94-3367.73) | 11.14(6.77-16.81) | 0.74*(0.71-0.78) |
| American Samoa | 1.11(0.60-1.81) | 7.16(4.01-11.52) | 1.70(0.99-2.64) | 7.78(4.42-12.21) | 0.25*(0.2-0.3) |
| Andorra | 3.21(1.87-4.96) | 11.83(6.97-18.15) | 6.67(4.25-10.08) | 13.12(8.08-20.19) | 0.32*(0.23-0.42) |
| Angola | 241.66(136.57-380.78) | 8.43(4.91-13.08) | 845.70(482.69-1357.66) | 9.17(5.37-14.53) | 0.28*(0.25-0.3) |
| Antigua and Barbuda | 3.05(1.87-4.62) | 14.51(9.11-21.67) | 6.89(4.27-10.49) | 15.30(9.37-23.44) | 0.17*(0.06-0.27) |
| Argentina | 727.38(384.01-1202.67) | 5.63(2.97-9.30) | 1444.28(796.52-2333.91) | 6.92(3.79-11.22) | 0.71*(0.63-0.79) |
| Armenia | 72.63(37.86-124.66) | 6.68(3.70-11.13) | 93.33(50.99-150.30) | 6.85(3.77-10.98) | 0.08*(0.06-0.09) |
| Australia | 329.46(162.47-574.94) | 4.28(2.12-7.46) | 650.23(348.34-1073.76) | 5.57(2.95-9.28) | 0.90*(0.76-1.03) |
| Austria | 407.39(246.04-622.92) | 11.41(6.84-17.53) | 518.06(319.60-786.63) | 12.50(7.60-19.16) | 0.30*(0.28-0.31) |
| Azerbaijan | 141.95(73.06-238.83) | 6.92(3.82-11.23) | 331.23(178.21-537.67) | 6.80(3.70-10.95) | -0.07*(-0.08--0.06) |
| Bahamas | 14.25(8.64-21.46) | 15.53(9.67-23.03) | 31.24(19.27-46.32) | 16.40(10.01-24.47) | 0.18*(0.06-0.29) |
| Bahrain | 15.87(8.39-26.19) | 8.44(4.81-13.25) | 99.08(59.37-156.84) | 10.27(6.11-16.32) | 0.63*(0.61-0.66) |
| Bangladesh | 2898.78(1646.63-4644.35) | 9.88(5.81-15.54) | 7774.89(4587.58-12099.94) | 11.35(6.74-17.58) | 0.42*(0.39-0.46) |
| Barbados | 13.33(7.92-19.89) | 13.83(8.41-20.35) | 21.62(13.74-32.67) | 15.33(9.55-23.45) | 0.32*(0.23-0.4) |
| Belarus | 313.62(172.16-510.18) | 7.82(4.37-12.57) | 353.16(199.63-578.13) | 7.65(4.27-12.64) | -0.03(-0.08-0.01) |
| Belgium | 491.39(294.22-746.19) | 11.41(6.86-17.26) | 675.49(419.21-1032.46) | 13.35(8.15-20.59) | 0.50*(0.44-0.56) |
| Belize | 6.37(3.76-9.91) | 13.84(8.45-21.07) | 27.21(17.04-41.05) | 15.67(9.88-23.53) | 0.37*(0.29-0.45) |
| Benin | 92.07(51.85-147.32) | 7.71(4.49-12.12) | 332.06(193.03-523.62) | 8.73(5.23-13.58) | 0.41*(0.39-0.43) |
| Bermuda | 4.32(2.59-6.65) | 14.80(8.96-22.67) | 4.94(3.16-7.41) | 15.68(9.78-23.94) | 0.17*(0.05-0.28) |
| Bhutan | 17.11(9.66-27.40) | 10.30(6.06-16.13) | 40.64(23.39-64.29) | 12.24(7.17-19.17) | 0.55*(0.53-0.56) |
| Bolivia | 555.82(370.59-783.62) | 29.19(19.80-40.67) | 1416.88(923.29-2068.33) | 29.77(19.54-43.23) | 0.04(-0.08-0.15) |
| Bosnia and Herzegovina | 117.57(62.77-200.15) | 6.48(3.53-10.90) | 100.54(56.29-160.42) | 6.21(3.39-10.07) | -0.13*(-0.16--0.1) |
| Botswana | 29.66(17.06-46.69) | 8.68(5.16-13.41) | 103.62(62.00-160.02) | 9.62(5.85-14.74) | 0.33*(0.3-0.36) |
| Brazil | 6782.17(4349.93-9807.89) | 13.27(8.66-19.00) | 17889.86(11947.80-25338.25) | 16.77(11.16-23.80) | 0.72*(0.52-0.91) |
| Brunei Darussalam | 6.99(3.79-11.51) | 7.56(4.36-11.98) | 22.14(12.85-34.25) | 9.25(5.39-14.26) | 0.66*(0.61-0.7) |
| Bulgaria | 207.49(112.61-333.21) | 5.23(2.81-8.47) | 216.62(125.85-337.06) | 6.19(3.48-9.88) | 0.54*(0.49-0.59) |
| Burkina Faso | 177.64(97.81-280.80) | 7.39(4.16-11.56) | 532.36(303.63-833.50) | 8.41(4.93-12.97) | 0.43*(0.4-0.46) |
| Burundi | 125.18(71.78-202.17) | 8.91(5.35-13.99) | 356.48(206.43-562.70) | 9.41(5.64-14.58) | 0.16*(0.12-0.19) |
| Cabo Verde | 5.74(3.17-9.22) | 8.04(4.73-12.50) | 25.24(15.20-39.04) | 10.53(6.45-16.12) | 0.88*(0.84-0.92) |
| Cambodia | 251.79(147.24-401.51) | 9.02(5.44-14.12) | 641.27(376.72-1022.67) | 9.63(5.78-15.15) | 0.21*(0.18-0.24) |
| Cameroon | 221.40(126.94-350.07) | 8.03(4.74-12.48) | 893.93(522.28-1394.41) | 9.25(5.57-14.21) | 0.46*(0.44-0.47) |
| Canada | 1237.15(794.61-1740.09) | 9.72(6.30-13.58) | 2433.03(1690.07-3264.52) | 14.63(10.07-19.78) | 1.35*(1.23-1.47) |
| Central African Republic | 67.72(38.73-107.38) | 8.98(5.28-14.00) | 168.58(99.20-261.32) | 9.61(5.75-14.75) | 0.22*(0.21-0.24) |
| Chad | 109.47(59.55-177.00) | 7.23(4.05-11.51) | 349.19(201.85-543.69) | 8.22(4.90-12.61) | 0.42*(0.37-0.46) |
| Chile | 267.83(139.29-442.90) | 5.28(2.80-8.61) | 591.75(331.21-938.95) | 6.84(3.81-10.91) | 0.84*(0.73-0.94) |
| China | 42860.57(27272.86-63384.01) | 9.74(6.33-14.23) | 162648.66(112605.30-229007.80) | 21.63(14.84-30.64) | 2.59*(2.5-2.68) |
| Colombia | 1477.51(878.47-2308.32) | 13.81(8.44-21.24) | 3530.15(2187.14-5308.34) | 16.54(10.28-24.82) | 0.57*(0.51-0.63) |
| Comoros | 10.65(6.13-16.56) | 8.84(5.21-13.56) | 26.85(15.74-42.89) | 9.13(5.39-14.54) | 0.12*(0.1-0.13) |
| Congo | 58.58(33.61-91.41) | 9.50(5.64-14.54) | 209.03(121.43-325.68) | 9.78(5.72-15.17) | 0.09*(0.08-0.11) |
| Cook Islands | 0.56(0.32-0.88) | 8.45(4.91-13.21) | 0.68(0.40-1.05) | 9.14(5.34-14.37) | 0.25*(0.22-0.28) |
| Costa Rica | 144.55(85.21-232.53) | 15.02(9.12-23.68) | 384.49(234.88-584.75) | 18.05(11.08-27.38) | 0.56*(0.53-0.59) |
| Côte d'Ivoire | 271.63(151.04-422.18) | 8.34(4.82-12.73) | 849.28(510.17-1324.71) | 9.17(5.62-14.09) | 0.31*(0.29-0.32) |
| Croatia | 130.14(69.28-214.11) | 5.90(3.14-9.71) | 119.42(65.97-192.76) | 6.02(3.26-9.87) | 0.07*(0.03-0.12) |
| Cuba | 543.36(329.36-849.81) | 12.14(7.41-18.91) | 764.29(470.95-1147.46) | 14.29(8.60-21.82) | 0.54*(0.48-0.6) |
| Cyprus | 40.08(24.11-61.56) | 11.84(7.14-18.15) | 86.32(51.03-132.55) | 12.09(7.12-18.59) | 0.06*(0.04-0.09) |
| Czechia | 286.22(149.14-475.20) | 5.72(2.94-9.58) | 356.19(197.84-573.70) | 6.04(3.23-9.99) | 0.17*(0.16-0.18) |
| Democratic People's Republic of Korea | 906.97(569.49-1370.18) | 10.56(6.61-16.03) | 2063.41(1327.63-3023.82) | 15.36(9.78-22.70) | 1.22*(1.2-1.24) |
| Democratic Republic of the Congo | 773.84(426.30-1230.23) | 8.00(4.56-12.44) | 2448.22(1420.46-3861.02) | 9.06(5.38-14.07) | 0.40*(0.37-0.43) |
| Denmark | 267.40(163.40-403.38) | 10.58(6.38-16.08) | 335.14(209.81-503.48) | 13.22(8.14-20.08) | 0.72*(0.68-0.75) |
| Djibouti | 10.19(5.71-16.37) | 8.40(4.87-13.26) | 48.60(28.54-75.32) | 9.01(5.36-13.86) | 0.23*(0.19-0.27) |
| Dominica | 3.18(1.91-4.93) | 14.07(8.60-21.65) | 4.31(2.68-6.54) | 15.19(9.41-23.09) | 0.21*(0.12-0.31) |
| Dominican Republic | 275.25(163.26-421.77) | 12.89(7.88-19.37) | 712.21(438.61-1059.66) | 15.20(9.41-22.55) | 0.51*(0.41-0.61) |
| Ecuador | 633.81(380.21-957.12) | 20.81(12.76-31.00) | 2177.66(1420.55-3265.25) | 29.44(19.27-44.02) | 1.10*(1.02-1.18) |
| Egypt | 1618.96(963.52-2501.30) | 9.08(5.50-13.86) | 4989.36(3042.81-7499.48) | 12.49(7.71-18.58) | 1.03*(0.96-1.09) |
| El Salvador | 226.68(136.22-348.89) | 14.87(9.12-22.59) | 429.31(264.67-677.63) | 16.97(10.51-26.71) | 0.38*(0.29-0.47) |
| Equatorial Guinea | 9.55(5.47-15.30) | 8.34(4.85-13.27) | 42.94(24.03-68.91) | 9.48(5.57-14.82) | 0.42*(0.4-0.45) |
| Eritrea | 90.05(53.39-141.83) | 9.60(5.82-14.94) | 236.06(138.87-369.50) | 10.24(6.16-15.80) | 0.20*(0.17-0.24) |
| Estonia | 63.69(37.84-101.24) | 9.41(5.59-14.96) | 55.09(32.69-85.78) | 8.85(5.16-13.94) | -0.21*(-0.25--0.18) |
| Eswatini | 19.60(11.50-30.58) | 9.56(5.75-14.69) | 44.28(26.63-67.96) | 10.91(6.75-16.46) | 0.42*(0.39-0.45) |
| Ethiopia | 1316.44(841.66-1902.94) | 9.99(6.52-14.31) | 3063.70(1919.94-4527.70) | 9.36(6.02-13.65) | -0.21*(-0.23--0.18) |
| Fiji | 21.91(12.53-34.74) | 8.19(4.80-12.80) | 34.89(20.84-53.13) | 8.95(5.36-13.61) | 0.29*(0.26-0.31) |
| Finland | 294.76(178.40-450.40) | 11.66(7.00-17.88) | 313.81(191.50-481.65) | 13.64(8.24-21.05) | 0.50*(0.48-0.53) |
| France | 2744.90(1641.92-4227.55) | 11.10(6.66-17.03) | 3582.93(2173.20-5525.83) | 12.56(7.48-19.60) | 0.39*(0.37-0.42) |
| Gabon | 25.08(14.34-40.21) | 9.73(5.76-15.28) | 66.83(39.83-103.49) | 10.34(6.25-15.85) | 0.19*(0.16-0.22) |
| Gambia | 18.75(10.24-29.85) | 7.35(4.22-11.43) | 61.30(35.96-96.11) | 8.64(5.22-13.30) | 0.53*(0.48-0.58) |
| Georgia | 153.80(85.62-251.17) | 7.49(4.24-12.11) | 121.78(68.79-192.79) | 7.53(4.20-12.00) | 0(-0.04-0.04) |
| Germany | 4313.03(2676.83-6530.57) | 11.93(7.37-18.11) | 4984.98(3213.83-7200.09) | 14.06(8.98-20.45) | 0.54*(0.52-0.56) |
| Ghana | 311.09(172.58-499.02) | 7.35(4.22-11.60) | 950.21(538.71-1503.08) | 7.90(4.58-12.33) | 0.24*(0.2-0.28) |
| Greece | 491.07(295.91-755.52) | 11.00(6.58-16.98) | 625.19(396.43-942.22) | 12.50(7.69-19.18) | 0.42*(0.39-0.45) |
| Greenland | 1.83(1.07-2.89) | 7.12(4.21-11.14) | 1.65(1.00-2.55) | 7.50(4.59-11.50) | 0.18*(0.17-0.19) |
| Grenada | 3.06(1.80-4.68) | 13.32(8.13-19.99) | 6.79(4.29-10.15) | 14.89(9.31-22.42) | 0.32*(0.21-0.43) |
| Guam | 4.44(2.47-7.10) | 8.64(4.98-13.54) | 6.71(4.12-10.54) | 9.93(6.01-15.73) | 0.45*(0.41-0.49) |
| Guatemala | 297.18(178.01-457.06) | 13.66(8.38-20.73) | 899.35(541.87-1352.23) | 15.68(9.58-23.36) | 0.44*(0.41-0.47) |
| Guinea | 123.79(69.91-196.96) | 7.23(4.17-11.37) | 304.96(172.16-483.77) | 8.21(4.79-12.80) | 0.41*(0.39-0.43) |
| Guinea-Bissau | 22.35(13.01-35.17) | 8.70(5.22-13.44) | 56.67(32.83-88.21) | 9.33(5.58-14.20) | 0.25*(0.21-0.29) |
| Guyana | 32.69(19.36-50.85) | 13.70(8.37-20.91) | 46.76(29.33-70.63) | 15.04(9.44-22.70) | 0.29*(0.22-0.37) |
| Haiti | 263.44(160.51-397.88) | 13.93(8.68-20.72) | 737.23(453.47-1108.79) | 14.76(9.25-21.96) | 0.15*(0.02-0.29) |
| Honduras | 178.16(108.34-273.14) | 14.68(9.17-22.21) | 617.74(376.30-935.30) | 16.86(10.44-25.27) | 0.43*(0.35-0.51) |
| Hungary | 276.25(143.63-451.64) | 5.64(2.89-9.31) | 312.83(172.23-499.47) | 5.99(3.18-9.81) | 0.20*(0.16-0.24) |
| Iceland | 12.59(7.56-19.65) | 12.15(7.39-18.83) | 22.24(13.79-33.68) | 14.30(8.79-21.75) | 0.53*(0.51-0.56) |
| India | 33284.47(21171.65-48499.16) | 11.44(7.38-16.55) | 87266.00(57032.97-124739.24) | 14.30(9.39-20.40) | 0.70*(0.61-0.79) |
| Indonesia | 5594.84(3545.10-8267.65) | 9.01(5.83-13.18) | 12978.23(8411.79-18910.51) | 9.49(6.12-13.86) | 0.17*(0.14-0.2) |
| Iran (Islamic Republic of) | 1311.52(804.04-1973.92) | 9.12(5.78-13.44) | 5223.96(3392.60-7571.34) | 11.56(7.54-16.73) | 0.76*(0.7-0.82) |
| Iraq | 388.79(220.19-623.43) | 8.13(4.77-12.73) | 1795.75(1101.57-2786.78) | 10.71(6.59-16.58) | 0.90*(0.87-0.93) |
| Ireland | 168.55(101.46-264.65) | 11.82(7.13-18.51) | 335.49(209.53-503.31) | 13.75(8.41-20.85) | 0.48*(0.45-0.51) |
| Israel | 243.01(144.95-368.10) | 13.46(8.11-20.16) | 607.20(368.04-921.48) | 15.06(9.05-23.00) | 0.35*(0.26-0.44) |
| Italy | 3420.21(2277.37-4917.69) | 13.59(9.01-19.59) | 4074.21(2810.75-5721.60) | 14.67(9.86-20.89) | 0.24*(0.07-0.4) |
| Jamaica | 92.21(53.60-145.49) | 13.41(8.03-20.77) | 185.19(112.65-283.23) | 15.14(9.25-23.09) | 0.38*(0.28-0.49) |
| Japan | 5051.01(3207.75-7649.23) | 7.73(4.80-11.81) | 5015.41(3284.79-7411.46) | 8.29(5.24-12.50) | 0.21*(0.15-0.27) |
| Jordan | 69.38(38.92-110.78) | 7.55(4.39-11.78) | 411.48(243.62-645.40) | 7.89(4.70-12.33) | 0.12*(0.02-0.22) |
| Kazakhstan | 429.30(235.86-704.40) | 7.78(4.44-12.47) | 710.53(406.56-1116.54) | 8.34(4.77-13.10) | 0.21*(0.18-0.24) |
| Kenya | 482.13(301.55-713.96) | 8.87(5.73-12.93) | 1696.33(1094.63-2464.49) | 10.13(6.65-14.59) | 0.44*(0.43-0.46) |
| Kiribati | 2.01(1.20-3.09) | 8.74(5.35-13.17) | 3.95(2.38-6.06) | 8.87(5.44-13.42) | 0.05*(0.04-0.06) |
| Kuwait | 57.88(31.86-94.86) | 8.37(4.83-13.27) | 314.47(187.75-490.97) | 9.79(5.85-15.30) | 0.53*(0.46-0.59) |
| Kyrgyzstan | 83.25(43.02-141.03) | 7.06(3.90-11.53) | 176.86(95.11-290.82) | 6.69(3.66-10.89) | -0.19*(-0.22--0.16) |
| Lao People's Democratic Republic | 113.65(68.32-179.80) | 9.63(5.94-14.93) | 277.83(163.03-429.23) | 9.46(5.65-14.43) | -0.07*(-0.1--0.04) |
| Latvia | 99.06(57.42-156.63) | 8.38(4.81-13.31) | 69.52(40.69-108.73) | 7.95(4.56-12.62) | -0.15*(-0.19--0.11) |
| Lebanon | 82.63(46.89-129.45) | 8.00(4.57-12.49) | 243.78(142.11-381.52) | 9.63(5.71-14.92) | 0.60*(0.59-0.61) |
| Lesotho | 36.38(20.55-56.57) | 8.08(4.63-12.47) | 59.05(34.37-94.38) | 9.13(5.48-14.34) | 0.39*(0.37-0.42) |
| Liberia | 50.59(27.79-79.88) | 7.74(4.44-11.97) | 173.95(100.01-268.33) | 8.69(5.04-13.34) | 0.39*(0.35-0.43) |
| Libya | 90.37(52.23-142.45) | 8.04(4.78-12.40) | 448.45(275.90-670.58) | 11.53(7.05-17.32) | 1.16*(1.02-1.3) |
| Lithuania | 129.74(73.59-209.64) | 8.28(4.69-13.41) | 98.81(57.80-158.78) | 8.07(4.61-13.17) | -0.08*(-0.11--0.06) |
| Luxembourg | 20.99(12.62-32.06) | 11.62(6.99-17.72) | 43.29(26.02-66.27) | 13.14(7.81-20.27) | 0.39*(0.35-0.43) |
| Madagascar | 239.96(132.23-386.33) | 7.89(4.54-12.39) | 784.37(454.49-1221.25) | 8.78(5.21-13.50) | 0.35*(0.3-0.39) |
| Malawi | 220.41(122.17-350.53) | 8.75(5.03-13.62) | 516.92(298.85-800.28) | 9.21(5.46-14.02) | 0.17*(0.14-0.2) |
| Malaysia | 523.20(289.27-839.67) | 8.76(5.02-13.79) | 1437.30(836.58-2219.69) | 10.20(6.02-15.62) | 0.50*(0.48-0.51) |
| Maldives | 5.17(2.95-8.16) | 9.65(5.68-15.02) | 28.55(15.82-45.34) | 9.51(5.42-14.89) | -0.05*(-0.08--0.01) |
| Mali | 176.87(98.32-278.24) | 7.52(4.27-11.69) | 471.97(263.48-746.98) | 7.82(4.49-12.18) | 0.14*(0.1-0.17) |
| Malta | 19.90(11.92-30.88) | 11.14(6.65-17.26) | 27.26(16.85-41.89) | 13.24(8.06-20.46) | 0.55*(0.51-0.58) |
| Marshall Islands | 1.01(0.59-1.59) | 9.09(5.53-13.90) | 2.43(1.53-3.66) | 10.20(6.45-15.29) | 0.37*(0.35-0.4) |
| Mauritania | 40.91(22.49-64.85) | 7.39(4.20-11.51) | 100.79(57.09-160.91) | 7.74(4.49-12.20) | 0.16*(0.12-0.2) |
| Mauritius | 41.23(24.09-63.84) | 9.92(5.99-15.08) | 66.34(40.35-103.25) | 10.83(6.49-16.97) | 0.28*(0.27-0.3) |
| Mexico | 3739.90(2402.88-5365.70) | 14.59(9.57-20.70) | 10710.75(7245.75-14972.46) | 18.03(12.15-25.26) | 0.68*(0.63-0.73) |
| Micronesia (Federated States of) | 2.31(1.33-3.62) | 8.34(5.00-12.76) | 3.80(2.37-5.73) | 9.38(5.86-14.14) | 0.37*(0.32-0.41) |
| Monaco | 1.77(1.08-2.67) | 11.75(7.04-17.99) | 2.08(1.29-3.13) | 13.34(8.11-20.44) | 0.41*(0.35-0.47) |
| Mongolia | 39.47(21.48-65.94) | 6.95(3.95-11.31) | 99.67(54.57-159.72) | 6.57(3.61-10.52) | -0.18*(-0.22--0.15) |
| Montenegro | 15.64(8.38-25.80) | 6.36(3.46-10.38) | 18.75(10.32-29.70) | 6.36(3.43-10.23) | 0.02(-0.01-0.04) |
| Morocco | 644.84(378.00-1011.25) | 8.37(5.05-12.86) | 2011.34(1250.71-2982.40) | 12.07(7.50-17.92) | 1.19*(1.17-1.2) |
| Mozambique | 311.48(173.04-492.61) | 8.13(4.60-12.74) | 763.50(450.84-1179.25) | 9.11(5.52-13.84) | 0.37*(0.32-0.42) |
| Myanmar | 1097.85(624.34-1736.77) | 8.72(5.14-13.52) | 2336.57(1387.50-3638.90) | 9.66(5.72-15.07) | 0.33*(0.31-0.36) |
| Namibia | 33.44(19.05-52.97) | 8.81(5.19-13.72) | 86.15(50.04-133.73) | 9.51(5.64-14.62) | 0.25*(0.22-0.29) |
| Nauru | 0.32(0.19-0.49) | 9.36(5.81-14.17) | 0.41(0.25-0.61) | 10.23(6.44-15.28) | 0.28*(0.22-0.33) |
| Nepal | 515.01(295.38-815.61) | 8.76(5.14-13.71) | 1298.68(746.69-2050.51) | 11.11(6.46-17.41) | 0.77*(0.76-0.79) |
| Netherlands | 829.40(499.41-1308.63) | 11.73(7.06-18.46) | 994.68(611.04-1513.63) | 13.65(8.25-20.99) | 0.49*(0.46-0.51) |
| New Zealand | 60.81(33.22-97.66) | 4.10(2.25-6.56) | 115.57(68.91-180.21) | 5.19(3.07-8.13) | 0.76*(0.68-0.85) |
| Nicaragua | 137.26(81.41-212.39) | 14.34(8.80-21.73) | 444.84(271.06-681.20) | 16.59(10.23-25.20) | 0.46*(0.38-0.54) |
| Niger | 159.31(88.78-254.41) | 7.61(4.36-12.00) | 442.58(246.83-719.57) | 7.92(4.57-12.67) | 0.15*(0.06-0.25) |
| Nigeria | 2154.04(1357.22-3164.95) | 8.32(5.34-12.12) | 5933.44(3772.32-8653.60) | 8.90(5.75-12.87) | 0.23*(0.2-0.27) |
| Niue | 0.07(0.04-0.11) | 9.18(5.44-14.12) | 0.07(0.04-0.11) | 9.76(5.93-15.00) | 0.20*(0.17-0.23) |
| North Macedonia | 51.47(28.13-83.13) | 6.14(3.38-9.87) | 72.05(41.45-115.07) | 6.24(3.53-10.09) | 0.06*(0.05-0.07) |
| Northern Mariana Islands | 1.88(1.07-2.94) | 8.96(5.27-13.72) | 2.32(1.41-3.53) | 9.33(5.52-14.48) | 0.15*(0.02-0.28) |
| Norway | 136.19(87.70-193.55) | 7.09(4.55-10.10) | 291.34(196.24-418.83) | 11.77(7.85-17.04) | 1.65*(1.6-1.71) |
| Oman | 54.41(30.21-87.99) | 8.39(4.91-13.15) | 275.37(161.70-445.28) | 10.90(6.64-17.24) | 0.84*(0.79-0.89) |
| Pakistan | 3375.55(2114.03-4979.11) | 10.72(6.81-15.71) | 10631.47(6767.39-15529.26) | 12.60(8.14-18.27) | 0.50*(0.46-0.54) |
| Palau | 0.60(0.36-0.92) | 10.11(6.21-15.34) | 1.18(0.75-1.74) | 11.20(6.90-16.87) | 0.32*(0.27-0.38) |
| Palestine | 36.81(20.88-60.42) | 8.06(4.75-12.88) | 173.15(101.89-274.39) | 10.06(6.05-15.71) | 0.71*(0.68-0.75) |
| Panama | 122.79(73.98-192.63) | 15.21(9.35-23.60) | 336.16(205.42-517.54) | 18.58(11.32-28.65) | 0.65*(0.62-0.67) |
| Papua New Guinea | 92.82(51.95-149.14) | 7.52(4.33-11.85) | 323.59(191.27-504.19) | 8.43(5.06-13.01) | 0.37*(0.34-0.4) |
| Paraguay | 160.14(95.14-246.70) | 13.16(8.00-19.99) | 469.99(289.11-708.44) | 16.29(10.14-24.35) | 0.68*(0.59-0.76) |
| Peru | 1534.66(952.50-2283.08) | 23.13(14.63-33.93) | 4847.09(3131.36-7029.25) | 30.72(19.90-44.45) | 0.89*(0.86-0.93) |
| Philippines | 1854.84(1188.34-2707.89) | 9.49(6.21-13.68) | 4825.80(3179.92-7005.96) | 10.58(7.02-15.30) | 0.35*(0.33-0.37) |
| Poland | 1523.37(975.04-2267.32) | 9.47(6.12-14.00) | 1145.15(860.92-1483.51) | 5.78(4.31-7.52) | -1.60*(-1.65--1.54) |
| Portugal | 447.02(271.07-688.66) | 10.63(6.42-16.40) | 658.76(408.09-992.32) | 12.58(7.58-19.26) | 0.55*(0.5-0.59) |
| Puerto Rico | 211.27(127.99-323.51) | 14.09(8.54-21.56) | 232.04(144.48-349.77) | 15.88(9.73-24.20) | 0.37*(0.32-0.42) |
| Qatar | 19.38(10.61-31.99) | 9.00(5.24-14.23) | 216.95(123.66-354.01) | 10.76(6.32-17.22) | 0.58*(0.55-0.6) |
| Republic of Korea | 1160.19(603.60-1948.08) | 6.38(3.42-10.54) | 2267.33(1314.07-3515.25) | 8.19(4.61-12.92) | 0.83*(0.78-0.88) |
| Republic of Moldova | 149.49(82.98-238.38) | 8.63(4.87-13.63) | 151.74(85.59-240.92) | 7.89(4.40-12.63) | -0.29*(-0.31--0.26) |
| Romania | 565.37(306.52-910.75) | 5.97(3.24-9.60) | 627.59(368.54-998.42) | 6.47(3.67-10.54) | 0.23*(0.18-0.29) |
| Russian Federation | 4968.92(3058.35-7525.50) | 8.48(5.32-12.67) | 6151.93(3945.02-9103.19) | 8.43(5.34-12.56) | -0.02(-0.06-0.02) |
| Rwanda | 162.13(92.54-259.46) | 9.33(5.62-14.48) | 398.61(229.15-630.25) | 8.94(5.28-13.91) | -0.14*(-0.16--0.12) |
| Saint Kitts and Nevis | 1.57(0.91-2.45) | 14.51(8.93-21.98) | 4.92(3.04-7.52) | 15.97(9.75-24.63) | 0.29*(0.21-0.37) |
| Saint Lucia | 5.51(3.28-8.36) | 14.25(8.73-21.24) | 14.36(9.07-21.13) | 15.82(9.84-23.50) | 0.31*(0.23-0.39) |
| Saint Vincent and the Grenadines | 3.92(2.26-6.10) | 13.46(8.09-20.53) | 7.74(4.77-11.64) | 14.88(9.05-22.51) | 0.31*(0.2-0.41) |
| Samoa | 3.61(2.05-5.85) | 8.29(4.86-13.24) | 6.64(3.94-10.34) | 9.19(5.50-14.27) | 0.34*(0.32-0.36) |
| San Marino | 1.20(0.73-1.88) | 11.42(6.94-17.87) | 1.91(1.17-2.92) | 12.40(7.35-19.28) | 0.27*(0.24-0.29) |
| Sao Tome and Principe | 2.42(1.36-3.78) | 8.83(5.12-13.48) | 8.62(5.19-13.15) | 10.79(6.57-16.35) | 0.67*(0.59-0.75) |
| Saudi Arabia | 423.26(240.22-670.87) | 8.41(4.97-13.02) | 2927.83(1808.01-4475.31) | 12.31(7.66-18.74) | 1.23*(1.16-1.29) |
| Senegal | 141.15(76.45-224.17) | 7.35(4.12-11.48) | 418.88(238.74-652.62) | 8.64(5.04-13.25) | 0.55*(0.5-0.59) |
| Serbia | 244.29(130.80-405.30) | 5.81(3.10-9.66) | 252.04(136.53-405.76) | 5.79(3.08-9.44) | -0.01(-0.06-0.04) |
| Seychelles | 2.42(1.44-3.79) | 10.69(6.57-16.33) | 6.33(3.87-9.52) | 11.74(7.09-17.82) | 0.30*(0.28-0.33) |
| Sierra Leone | 81.71(44.57-132.40) | 6.95(3.93-11.06) | 216.20(123.15-339.30) | 7.96(4.67-12.27) | 0.43*(0.39-0.48) |
| Singapore | 105.34(56.84-172.37) | 7.26(4.01-11.72) | 281.43(160.54-446.06) | 8.17(4.58-13.07) | 0.39*(0.32-0.46) |
| Slovakia | 128.67(67.44-214.23) | 5.76(3.05-9.51) | 171.76(94.92-270.05) | 5.84(3.15-9.36) | 0.05*(0.03-0.07) |
| Slovenia | 56.81(31.11-92.79) | 6.34(3.48-10.37) | 66.05(36.26-106.23) | 6.42(3.42-10.53) | 0.04(0-0.09) |
| Solomon Islands | 7.75(4.59-12.07) | 8.97(5.45-13.76) | 25.05(15.68-37.41) | 9.98(6.28-14.85) | 0.35*(0.34-0.36) |
| Somalia | 200.11(113.46-316.95) | 8.88(5.19-13.86) | 549.98(312.17-861.08) | 9.13(5.32-14.09) | 0.09*(0.06-0.11) |
| South Africa | 1089.17(685.63-1604.55) | 9.15(5.91-13.33) | 2479.95(1613.21-3620.98) | 9.73(6.38-14.13) | 0.19*(0.16-0.22) |
| South Sudan | 125.66(68.68-201.94) | 8.35(4.75-13.16) | 253.83(149.36-392.60) | 8.47(4.99-13.09) | 0.04*(0.01-0.07) |
| Spain | 1722.45(1044.82-2659.01) | 10.79(6.53-16.66) | 3286.53(2022.76-5041.42) | 13.61(8.14-21.22) | 0.74*(0.69-0.79) |
| Sri Lanka | 592.30(341.05-951.83) | 9.02(5.31-14.30) | 1026.81(621.56-1589.94) | 10.23(6.12-15.92) | 0.40*(0.38-0.42) |
| Sudan | 483.81(285.79-751.16) | 8.81(5.34-13.45) | 1550.66(914.86-2377.50) | 10.92(6.60-16.47) | 0.69*(0.63-0.74) |
| Suriname | 15.15(9.04-23.25) | 11.89(7.22-18.02) | 34.99(21.61-53.55) | 13.75(8.42-21.15) | 0.45*(0.4-0.5) |
| Sweden | 487.43(327.59-696.90) | 11.71(7.76-16.89) | 557.53(366.01-807.49) | 12.51(8.16-18.21) | 0.21*(0.18-0.24) |
| Switzerland | 398.40(238.94-614.90) | 11.69(6.96-18.10) | 540.45(334.15-833.78) | 12.91(7.89-20.06) | 0.32*(0.3-0.34) |
| Syrian Arab Republic | 221.75(123.34-358.69) | 7.27(4.18-11.50) | 635.14(391.08-982.11) | 9.50(5.67-15.04) | 0.87*(0.86-0.88) |
| Taiwan (Province of China) | 608.38(373.86-911.52) | 7.77(4.96-11.34) | 1393.86(1012.21-1852.44) | 11.05(7.89-14.84) | 1.13*(0.96-1.31) |
| Tajikistan | 85.51(43.78-148.16) | 6.94(3.81-11.55) | 259.04(143.66-416.14) | 7.05(4.02-11.16) | 0.01(-0.02-0.04) |
| Thailand | 2023.93(1231.83-3055.31) | 9.39(5.85-14.00) | 3694.83(2354.98-5370.28) | 10.48(6.50-15.44) | 0.35*(0.33-0.37) |
| Timor-Leste | 20.21(11.40-32.02) | 8.23(4.81-12.82) | 38.06(21.61-59.74) | 8.84(5.09-13.76) | 0.23*(0.2-0.26) |
| Togo | 67.53(36.87-106.93) | 7.32(4.14-11.36) | 247.74(144.60-381.38) | 8.54(5.06-13.02) | 0.52*(0.46-0.58) |
| Tokelau | 0.04(0.02-0.06) | 8.17(4.76-12.93) | 0.05(0.03-0.08) | 8.92(5.38-13.72) | 0.28*(0.26-0.3) |
| Tonga | 2.06(1.17-3.39) | 7.67(4.40-12.51) | 3.17(1.85-4.94) | 8.68(5.08-13.56) | 0.41*(0.39-0.43) |
| Trinidad and Tobago | 57.38(33.84-88.42) | 13.30(8.01-20.27) | 98.13(61.20-145.11) | 14.56(9.03-21.61) | 0.29*(0.21-0.38) |
| Tunisia | 189.49(107.70-303.00) | 7.68(4.49-12.00) | 603.50(363.03-931.06) | 10.34(6.18-16.00) | 0.97*(0.95-0.98) |
| Türkiye | 1592.36(926.06-2521.44) | 8.54(5.09-13.29) | 4331.46(2676.91-6641.05) | 10.46(6.40-16.13) | 0.65*(0.63-0.67) |
| Turkmenistan | 64.41(33.04-109.13) | 6.78(3.69-11.09) | 157.84(90.49-249.96) | 7.34(4.24-11.57) | 0.23*(0.21-0.26) |
| Tuvalu | 0.28(0.17-0.44) | 8.46(5.08-13.04) | 0.45(0.27-0.70) | 9.65(5.86-14.98) | 0.42*(0.38-0.46) |
| Uganda | 351.49(195.53-560.75) | 8.79(5.08-13.72) | 1061.26(615.41-1696.19) | 9.53(5.73-14.94) | 0.25*(0.22-0.28) |
| Ukraine | 1984.40(1224.71-2951.82) | 9.18(5.68-13.63) | 2133.50(1354.32-3122.58) | 9.49(5.95-14.02) | 0.11*(0.09-0.13) |
| United Arab Emirates | 87.38(50.13-137.62) | 10.32(6.26-15.62) | 1399.74(894.07-2089.80) | 14.54(9.19-21.87) | 1.11*(1.08-1.13) |
| United Kingdom | 3615.37(2423.16-5098.35) | 14.26(9.53-20.12) | 4362.42(2935.98-6238.97) | 14.67(9.79-21.07) | 0.12*(0.07-0.16) |
| United Republic of Tanzania | 556.18(316.22-895.73) | 8.74(5.14-13.78) | 1676.44(990.62-2633.77) | 9.33(5.64-14.46) | 0.23*(0.2-0.25) |
| United States of America | 11315.22(7262.96-16781.76) | 9.91(6.40-14.62) | 8721.39(6747.52-10952.78) | 6.14(4.72-7.74) | -1.52*(-1.57--1.48) |
| United States Virgin Islands | 8.40(5.41-12.49) | 16.33(10.37-24.51) | 6.78(4.52-9.69) | 18.52(12.05-26.96) | 0.38*(0.31-0.45) |
| Uruguay | 70.51(39.04-115.23) | 5.71(3.16-9.35) | 113.96(63.29-183.11) | 7.46(4.09-12.07) | 0.87*(0.82-0.93) |
| Uzbekistan | 389.79(208.39-649.33) | 7.39(4.21-11.96) | 1065.17(589.75-1711.89) | 7.16(4.01-11.43) | -0.10*(-0.12--0.08) |
| Vanuatu | 3.93(2.29-6.01) | 8.96(5.37-13.48) | 11.19(6.70-16.78) | 9.97(6.03-14.86) | 0.34*(0.33-0.36) |
| Venezuela (Bolivarian Republic of) | 828.55(490.30-1291.12) | 13.90(8.50-21.22) | 2183.26(1375.94-3256.74) | 16.91(10.54-25.39) | 0.62*(0.53-0.72) |
| Viet Nam | 1732.68(969.62-2738.15) | 9.56(5.66-14.55) | 5707.45(3511.18-8821.96) | 11.76(7.22-18.21) | 0.68*(0.66-0.69) |
| Yemen | 248.18(144.29-394.72) | 7.69(4.62-11.95) | 1073.49(650.89-1638.12) | 9.88(6.17-14.77) | 0.81*(0.78-0.85) |
| Zambia | 172.40(99.56-273.70) | 9.08(5.42-14.13) | 575.10(322.81-891.37) | 9.78(5.67-14.87) | 0.23*(0.2-0.26) |
| Zimbabwe | 227.74(131.47-357.56) | 8.99(5.39-13.81) | 494.99(291.44-772.66) | 9.58(5.75-14.75) | 0.18*(0.11-0.25) |

Note: UI: uncertainty interval; CI, confidence interval; AAPC, average annual percent of change; ASPR, age-standardized prevalence rate

Table S2. The case number and age-standardized rate of incidence among 204 countries and territories, with the AAPCs between 1990 and 2021

|  | 1990 | | 2021 | |  |
| --- | --- | --- | --- | --- | --- |
|  | Number (95%UI) | ASIR per 100,000 populations (95%UI) | Number (95%UI) | ASIR per 100,000 populations (95%UI) | AAPCs (95%CI) |
| Afghanistan | 48.14(28.73-73.07) | 1.70(1.00-2.59) | 188.36(108.55-295.32) | 2.00(1.17-3.09) | 0.52*(0.47-0.57) |
| Albania | 11.98(4.95-21.21) | 1.06(0.45-1.85) | 11.83(5.20-20.81) | 1.07(0.47-1.87) | 0.02(-0.05-0.09) |
| Algeria | 89.84(45.28-152.11) | 1.35(0.71-2.24) | 342.88(178.51-559.92) | 1.71(0.89-2.80) | 0.76*(0.74-0.78) |
| American Samoa | 0.17(0.08-0.30) | 1.08(0.49-1.85) | 0.26(0.13-0.43) | 1.19(0.57-2.02) | 0.33*(0.24-0.42) |
| Andorra | 0.43(0.20-0.74) | 1.59(0.73-2.70) | 0.90(0.45-1.55) | 1.82(0.88-3.14) | 0.42*(0.36-0.48) |
| Angola | 35.03(15.33-60.18) | 1.18(0.53-2.01) | 120.97(54.82-210.02) | 1.28(0.59-2.20) | 0.25*(0.21-0.29) |
| Antigua and Barbuda | 0.44(0.23-0.73) | 2.04(1.08-3.31) | 0.98(0.52-1.64) | 2.21(1.16-3.70) | 0.25*(0.11-0.4) |
| Argentina | 111.81(45.76-200.61) | 0.86(0.35-1.55) | 218.37(96.57-380.27) | 1.05(0.46-1.83) | 0.67*(0.56-0.79) |
| Armenia | 11.22(4.67-20.59) | 1.00(0.43-1.81) | 13.82(6.06-23.99) | 1.02(0.44-1.77) | 0.05*(0.02-0.08) |
| Australia | 45.39(15.94-84.61) | 0.59(0.21-1.11) | 90.17(36.59-165.53) | 0.78(0.32-1.44) | 0.91*(0.8-1.01) |
| Austria | 54.94(27.23-93.13) | 1.53(0.76-2.60) | 70.66(34.34-119.01) | 1.72(0.83-2.91) | 0.40*(0.36-0.43) |
| Azerbaijan | 22.13(9.00-39.49) | 1.03(0.44-1.80) | 48.63(21.41-86.13) | 1.00(0.44-1.76) | -0.13*(-0.15--0.11) |
| Bahamas | 2.10(1.11-3.43) | 2.21(1.21-3.57) | 4.46(2.36-7.27) | 2.37(1.25-3.87) | 0.23*(0.12-0.34) |
| Bahrain | 2.54(1.15-4.34) | 1.29(0.61-2.15) | 14.98(7.42-25.34) | 1.56(0.77-2.66) | 0.63*(0.6-0.66) |
| Bangladesh | 415.29(188.71-739.48) | 1.36(0.63-2.40) | 1126.12(536.97-1919.93) | 1.63(0.78-2.77) | 0.58*(0.57-0.6) |
| Barbados | 1.90(0.96-3.09) | 1.91(0.98-3.10) | 2.98(1.59-4.76) | 2.16(1.14-3.50) | 0.40*(0.32-0.48) |
| Belarus | 51.20(23.13-90.81) | 1.27(0.58-2.24) | 56.90(25.87-99.37) | 1.25(0.56-2.20) | -0.01(-0.09-0.07) |
| Belgium | 65.59(31.10-111.58) | 1.53(0.72-2.60) | 91.33(45.79-156.72) | 1.83(0.91-3.15) | 0.58*(0.51-0.66) |
| Belize | 0.94(0.46-1.57) | 1.93(0.99-3.20) | 4.03(2.14-6.56) | 2.30(1.23-3.72) | 0.52*(0.46-0.58) |
| Benin | 12.29(5.30-21.93) | 0.97(0.43-1.72) | 44.06(20.13-76.44) | 1.11(0.51-1.91) | 0.45*(0.41-0.49) |
| Bermuda | 0.62(0.31-1.03) | 2.11(1.06-3.50) | 0.70(0.37-1.12) | 2.28(1.18-3.72) | 0.25*(0.13-0.38) |
| Bhutan | 2.44(1.06-4.26) | 1.39(0.62-2.39) | 5.83(2.73-10.08) | 1.72(0.81-2.97) | 0.66*(0.6-0.73) |
| Bolivia (Plurinational State of) | 85.18(54.00-122.28) | 4.34(2.81-6.16) | 245.80(147.09-370.46) | 5.13(3.08-7.70) | 0.54*(0.46-0.62) |
| Bosnia and Herzegovina | 19.18(8.27-33.59) | 1.05(0.46-1.83) | 16.26(7.45-28.10) | 1.01(0.45-1.77) | -0.11*(-0.18--0.04) |
| Botswana | 4.16(1.85-7.32) | 1.16(0.53-2.03) | 14.33(6.87-24.72) | 1.31(0.63-2.26) | 0.40*(0.38-0.42) |
| Brazil | 978.66(534.71-1523.80) | 1.86(1.03-2.88) | 2576.90(1465.16-3927.88) | 2.43(1.37-3.71) | 0.85*(0.62-1.07) |
| Brunei Darussalam | 1.09(0.47-1.92) | 1.15(0.51-1.97) | 3.32(1.56-5.60) | 1.39(0.65-2.34) | 0.64*(0.61-0.68) |
| Bulgaria | 36.02(17.11-61.39) | 0.91(0.43-1.56) | 33.66(15.36-56.16) | 0.98(0.43-1.66) | 0.22*(0.14-0.3) |
| Burkina Faso | 23.22(9.46-41.66) | 0.93(0.39-1.67) | 69.97(31.00-120.88) | 1.06(0.47-1.81) | 0.45*(0.41-0.48) |
| Burundi | 18.04(8.50-31.81) | 1.22(0.60-2.12) | 49.13(22.12-84.30) | 1.24(0.57-2.11) | 0.02(-0.01-0.06) |
| Cabo Verde | 0.79(0.33-1.38) | 0.99(0.43-1.70) | 3.21(1.46-5.51) | 1.30(0.58-2.22) | 0.88*(0.82-0.94) |
| Cambodia | 37.74(17.70-64.47) | 1.31(0.63-2.21) | 93.75(42.37-161.30) | 1.39(0.63-2.36) | 0.18*(0.16-0.2) |
| Cameroon | 29.37(12.61-51.93) | 1.02(0.45-1.79) | 118.73(51.98-206.80) | 1.17(0.52-2.04) | 0.45*(0.37-0.53) |
| Canada | 200.74(112.16-300.81) | 1.57(0.88-2.36) | 399.26(240.49-582.09) | 2.42(1.45-3.55) | 1.40*(1.26-1.54) |
| Central African Republic | 10.24(4.87-17.29) | 1.32(0.64-2.20) | 24.70(12.14-41.15) | 1.38(0.69-2.29) | 0.16*(0.14-0.18) |
| Chad | 14.49(6.01-26.13) | 0.91(0.39-1.64) | 46.31(19.62-80.08) | 1.04(0.45-1.78) | 0.44*(0.35-0.53) |
| Chile | 40.59(16.22-72.93) | 0.79(0.32-1.41) | 91.22(40.41-155.55) | 1.06(0.47-1.81) | 0.95*(0.81-1.09) |
| China | 7144.15(3896.38-11301.28) | 1.61(0.89-2.51) | 34314.28(21228.18-50279.80) | 4.62(2.85-6.80) | 3.45*(3.29-3.6) |
| Colombia | 212.54(105.18-361.00) | 1.91(0.97-3.21) | 521.42(273.66-842.82) | 2.43(1.28-3.92) | 0.79*(0.67-0.9) |
| Comoros | 1.47(0.65-2.53) | 1.17(0.53-2.01) | 3.60(1.65-6.37) | 1.21(0.56-2.14) | 0.10*(0.09-0.12) |
| Congo | 8.80(4.29-14.78) | 1.38(0.70-2.27) | 30.07(14.30-51.09) | 1.40(0.67-2.37) | 0.04*(0.01-0.07) |
| Cook Islands | 0.09(0.04-0.15) | 1.36(0.66-2.25) | 0.11(0.05-0.18) | 1.44(0.68-2.47) | 0.20*(0.16-0.24) |
| Costa Rica | 21.67(10.90-37.03) | 2.17(1.11-3.65) | 58.16(30.62-93.81) | 2.72(1.43-4.38) | 0.73*(0.69-0.76) |
| Côte d'Ivoire | 36.52(15.59-62.92) | 1.06(0.46-1.81) | 110.69(50.17-189.75) | 1.15(0.52-1.97) | 0.29*(0.25-0.33) |
| Croatia | 21.16(9.33-37.03) | 0.96(0.42-1.69) | 19.24(8.59-33.76) | 0.98(0.43-1.74) | 0.05*(0.04-0.07) |
| Cuba | 74.95(38.20-124.36) | 1.66(0.86-2.73) | 106.56(54.35-176.69) | 2.05(1.04-3.41) | 0.68*(0.59-0.78) |
| Cyprus | 5.71(2.76-9.71) | 1.68(0.81-2.87) | 11.90(5.67-20.52) | 1.69(0.80-2.93) | 0.02*(0.01-0.04) |
| Czechia | 45.28(19.30-80.83) | 0.91(0.38-1.64) | 56.29(24.69-101.71) | 0.97(0.42-1.77) | 0.19*(0.16-0.22) |
| Democratic People's Republic of Korea | 160.50(92.78-252.73) | 1.86(1.08-2.93) | 382.12(220.92-582.05) | 2.86(1.64-4.37) | 1.39*(1.37-1.41) |
| Democratic Republic of the Congo | 112.61(50.00-196.54) | 1.12(0.51-1.92) | 351.58(163.24-613.79) | 1.26(0.60-2.19) | 0.40*(0.37-0.43) |
| Denmark | 35.82(17.18-59.92) | 1.43(0.68-2.40) | 46.01(23.03-79.33) | 1.82(0.90-3.15) | 0.77*(0.74-0.8) |
| Djibouti | 1.40(0.60-2.48) | 1.10(0.48-1.94) | 6.65(3.06-11.49) | 1.22(0.56-2.11) | 0.32*(0.29-0.35) |
| Dominica | 0.46(0.23-0.75) | 1.97(1.02-3.21) | 0.61(0.32-1.02) | 2.16(1.12-3.60) | 0.27*(0.16-0.38) |
| Dominican Republic | 39.03(18.76-64.30) | 1.74(0.86-2.83) | 101.82(52.69-163.94) | 2.16(1.12-3.47) | 0.68*(0.58-0.78) |
| Ecuador | 94.61(50.70-151.77) | 3.00(1.65-4.76) | 365.30(218.38-563.88) | 4.92(2.95-7.57) | 1.61*(1.57-1.66) |
| Egypt | 268.83(140.46-437.50) | 1.49(0.79-2.40) | 878.50(499.91-1387.46) | 2.19(1.26-3.43) | 1.24*(1.18-1.29) |
| El Salvador | 31.76(15.68-52.92) | 2.02(1.02-3.32) | 63.01(32.83-106.31) | 2.48(1.30-4.17) | 0.65*(0.55-0.75) |
| Equatorial Guinea | 1.40(0.64-2.44) | 1.20(0.57-2.06) | 6.37(2.85-11.13) | 1.34(0.62-2.31) | 0.38*(0.33-0.43) |
| Eritrea | 12.57(5.80-21.86) | 1.30(0.62-2.23) | 32.86(15.66-55.99) | 1.38(0.67-2.35) | 0.21*(0.19-0.23) |
| Estonia | 10.62(5.09-17.77) | 1.57(0.75-2.63) | 8.85(4.16-14.96) | 1.44(0.67-2.45) | -0.30*(-0.35--0.26) |
| Eswatini | 2.76(1.30-4.65) | 1.30(0.62-2.17) | 6.29(3.25-10.34) | 1.50(0.80-2.44) | 0.48*(0.44-0.51) |
| Ethiopia | 183.54(95.97-292.43) | 1.34(0.72-2.12) | 417.59(208.64-677.23) | 1.22(0.62-1.97) | -0.32*(-0.36--0.28) |
| Fiji | 3.61(1.77-6.08) | 1.33(0.67-2.23) | 5.61(2.91-9.12) | 1.44(0.75-2.34) | 0.24*(0.21-0.27) |
| Finland | 38.81(18.19-67.09) | 1.56(0.72-2.71) | 42.91(21.77-73.99) | 1.89(0.95-3.26) | 0.62*(0.59-0.64) |
| France | 367.01(176.76-632.71) | 1.49(0.71-2.57) | 491.77(233.65-845.85) | 1.75(0.83-3.02) | 0.51*(0.47-0.55) |
| Gabon | 3.78(1.75-6.57) | 1.42(0.69-2.41) | 9.78(4.75-16.59) | 1.49(0.73-2.51) | 0.15*(0.07-0.22) |
| Gambia | 2.48(1.02-4.37) | 0.91(0.38-1.60) | 8.20(3.64-14.34) | 1.10(0.49-1.91) | 0.61*(0.53-0.68) |
| Georgia | 23.64(10.17-41.42) | 1.14(0.50-1.98) | 18.06(8.15-31.45) | 1.13(0.50-1.98) | -0.04*(-0.08--0.01) |
| Germany | 594.29(298.95-984.55) | 1.64(0.83-2.72) | 723.38(407.65-1117.70) | 2.06(1.15-3.20) | 0.74*(0.7-0.78) |
| Ghana | 40.25(16.46-71.49) | 0.91(0.38-1.60) | 124.33(52.61-218.11) | 1.00(0.43-1.75) | 0.34*(0.3-0.38) |
| Greece | 66.16(32.05-114.70) | 1.49(0.72-2.58) | 85.04(43.19-146.96) | 1.74(0.86-3.03) | 0.53*(0.5-0.56) |
| Greenland | 0.33(0.18-0.54) | 1.29(0.71-2.08) | 0.30(0.16-0.48) | 1.36(0.74-2.15) | 0.19*(0.13-0.24) |
| Grenada | 0.44(0.21-0.73) | 1.82(0.91-3.01) | 0.95(0.50-1.57) | 2.11(1.12-3.48) | 0.46*(0.34-0.58) |
| Guam | 0.72(0.33-1.24) | 1.37(0.65-2.37) | 1.08(0.56-1.86) | 1.60(0.82-2.76) | 0.48*(0.46-0.5) |
| Guatemala | 41.93(21.01-69.94) | 1.86(0.96-3.08) | 130.83(68.39-210.72) | 2.24(1.18-3.58) | 0.59*(0.53-0.65) |
| Guinea | 16.44(7.05-29.18) | 0.93(0.40-1.65) | 41.05(17.36-72.48) | 1.05(0.45-1.86) | 0.40*(0.32-0.48) |
| Guinea-Bissau | 3.06(1.41-5.36) | 1.13(0.53-1.97) | 7.74(3.59-13.18) | 1.20(0.56-2.04) | 0.23*(0.19-0.27) |
| Guyana | 4.79(2.44-7.94) | 1.92(1.01-3.15) | 6.83(3.70-10.78) | 2.19(1.19-3.45) | 0.42*(0.35-0.5) |
| Haiti | 38.57(19.66-62.42) | 1.98(1.04-3.17) | 106.47(55.98-174.86) | 2.09(1.11-3.42) | 0.14*(0.02-0.26) |
| Honduras | 26.07(13.28-43.75) | 2.08(1.09-3.45) | 96.22(51.37-159.06) | 2.58(1.40-4.24) | 0.69*(0.64-0.74) |
| Hungary | 42.68(17.89-74.77) | 0.88(0.37-1.55) | 49.19(22.10-87.05) | 0.95(0.42-1.71) | 0.24*(0.23-0.26) |
| Iceland | 1.72(0.81-2.97) | 1.65(0.77-2.85) | 3.01(1.50-5.16) | 1.95(0.97-3.34) | 0.56*(0.52-0.6) |
| India | 4614.05(2359.77-7347.81) | 1.55(0.80-2.46) | 12395.28(6854.77-19369.41) | 2.02(1.12-3.15) | 0.80*(0.74-0.86) |
| Indonesia | 806.64(416.05-1296.72) | 1.26(0.66-2.02) | 1842.82(966.65-2953.70) | 1.35(0.71-2.18) | 0.23*(0.17-0.29) |
| Iran (Islamic Republic of) | 203.34(106.80-327.63) | 1.36(0.73-2.17) | 799.11(446.98-1245.07) | 1.77(0.99-2.76) | 0.83*(0.77-0.88) |
| Iraq | 61.64(30.16-104.22) | 1.25(0.63-2.08) | 281.49(152.86-459.77) | 1.67(0.91-2.72) | 0.94*(0.91-0.97) |
| Ireland | 23.10(11.34-39.86) | 1.62(0.79-2.80) | 46.39(22.67-78.45) | 1.94(0.93-3.29) | 0.57*(0.55-0.6) |
| Israel | 35.04(16.62-59.73) | 1.94(0.91-3.32) | 86.87(43.32-147.29) | 2.16(1.07-3.68) | 0.34*(0.24-0.44) |
| Italy | 451.27(240.07-722.46) | 1.80(0.95-2.89) | 441.08(218.31-723.58) | 1.67(0.84-2.73) | -0.28*(-0.43--0.13) |
| Jamaica | 13.34(6.17-22.70) | 1.86(0.89-3.12) | 26.43(13.72-43.91) | 2.15(1.12-3.55) | 0.44*(0.35-0.53) |
| Japan | 711.18(356.28-1148.16) | 1.11(0.54-1.81) | 667.07(345.18-1069.22) | 1.15(0.59-1.86) | 0.08(-0.06-0.22) |
| Jordan | 11.05(5.25-18.90) | 1.16(0.58-1.94) | 64.46(32.47-107.98) | 1.23(0.62-2.05) | 0.16*(0.05-0.27) |
| Kazakhstan | 66.52(29.16-117.48) | 1.18(0.53-2.06) | 103.69(47.00-184.09) | 1.22(0.55-2.17) | 0.12*(0.1-0.14) |
| Kenya | 66.48(33.06-106.83) | 1.15(0.58-1.84) | 231.59(122.00-370.51) | 1.34(0.71-2.14) | 0.49*(0.47-0.5) |
| Kiribati | 0.36(0.19-0.57) | 1.54(0.85-2.42) | 0.68(0.38-1.09) | 1.52(0.86-2.41) | -0.01(-0.05-0.02) |
| Kuwait | 9.15(4.20-15.89) | 1.28(0.60-2.19) | 47.33(22.41-79.26) | 1.48(0.69-2.49) | 0.48*(0.45-0.5) |
| Kyrgyzstan | 13.20(5.50-23.62) | 1.08(0.47-1.88) | 26.64(11.18-47.15) | 1.00(0.42-1.75) | -0.27*(-0.29--0.24) |
| Lao People's Democratic Republic | 17.17(8.63-28.89) | 1.42(0.73-2.35) | 40.89(19.43-69.70) | 1.37(0.66-2.32) | -0.11*(-0.14--0.08) |
| Latvia | 16.14(7.76-27.56) | 1.37(0.66-2.34) | 11.14(5.42-19.01) | 1.29(0.62-2.22) | -0.18*(-0.23--0.13) |
| Lebanon | 12.72(6.12-21.88) | 1.22(0.60-2.08) | 38.72(19.27-63.86) | 1.52(0.76-2.49) | 0.69*(0.64-0.74) |
| Lesotho | 4.86(2.14-8.48) | 1.06(0.47-1.84) | 8.44(4.05-14.34) | 1.27(0.62-2.13) | 0.59*(0.57-0.61) |
| Liberia | 6.70(2.74-11.90) | 0.97(0.41-1.70) | 22.03(9.45-38.44) | 1.08(0.47-1.89) | 0.39*(0.27-0.5) |
| Libya | 14.43(7.19-24.05) | 1.25(0.64-2.04) | 69.93(38.13-110.92) | 1.81(0.98-2.89) | 1.19*(1.14-1.24) |
| Lithuania | 20.77(9.43-36.27) | 1.33(0.61-2.31) | 15.77(7.26-27.20) | 1.30(0.60-2.26) | -0.06*(-0.08--0.04) |
| Luxembourg | 2.81(1.35-4.87) | 1.56(0.75-2.71) | 5.80(2.77-9.92) | 1.79(0.85-3.07) | 0.43*(0.38-0.48) |
| Madagascar | 33.11(14.83-57.95) | 1.03(0.48-1.78) | 106.65(49.27-185.31) | 1.15(0.54-1.99) | 0.37*(0.33-0.4) |
| Malawi | 30.48(13.41-53.04) | 1.15(0.52-1.98) | 71.33(33.08-123.31) | 1.22(0.57-2.09) | 0.19*(0.16-0.21) |
| Malaysia | 75.11(32.54-133.81) | 1.22(0.54-2.17) | 203.91(91.80-346.79) | 1.43(0.65-2.43) | 0.51*(0.48-0.55) |
| Maldives | 0.78(0.36-1.35) | 1.41(0.68-2.41) | 4.04(1.83-7.18) | 1.34(0.60-2.38) | -0.16*(-0.2--0.12) |
| Mali | 22.85(9.59-40.09) | 0.94(0.40-1.64) | 62.25(26.31-110.77) | 0.98(0.42-1.73) | 0.16*(0.11-0.2) |
| Malta | 2.62(1.26-4.47) | 1.48(0.71-2.55) | 3.66(1.83-6.41) | 1.80(0.89-3.17) | 0.63*(0.57-0.68) |
| Marshall Islands | 0.17(0.09-0.29) | 1.53(0.82-2.48) | 0.41(0.23-0.65) | 1.73(0.97-2.74) | 0.39*(0.3-0.48) |
| Mauritania | 5.53(2.38-9.56) | 0.95(0.42-1.64) | 13.35(5.84-23.63) | 0.99(0.44-1.74) | 0.12*(0.08-0.16) |
| Mauritius | 6.07(2.87-10.34) | 1.43(0.69-2.41) | 9.53(4.89-15.72) | 1.57(0.79-2.61) | 0.30*(0.28-0.32) |
| Mexico | 530.73(288.49-827.51) | 2.00(1.10-3.09) | 1520.74(876.38-2296.85) | 2.58(1.48-3.89) | 0.80*(0.66-0.95) |
| Micronesia (Federated States of) | 0.40(0.20-0.65) | 1.42(0.74-2.29) | 0.68(0.40-1.08) | 1.66(0.98-2.64) | 0.49*(0.46-0.52) |
| Monaco | 0.24(0.11-0.41) | 1.61(0.75-2.80) | 0.29(0.14-0.49) | 1.87(0.91-3.20) | 0.47*(0.4-0.53) |
| Mongolia | 6.11(2.58-11.05) | 1.04(0.46-1.83) | 14.95(6.70-26.13) | 0.99(0.44-1.73) | -0.16*(-0.22--0.11) |
| Montenegro | 2.51(1.08-4.48) | 1.02(0.44-1.81) | 2.93(1.24-5.02) | 1.00(0.42-1.73) | -0.04(-0.11-0.03) |
| Morocco | 101.92(51.66-170.83) | 1.29(0.68-2.13) | 326.88(189.45-505.40) | 1.96(1.14-3.04) | 1.35*(1.28-1.41) |
| Mozambique | 41.45(17.45-73.47) | 1.05(0.45-1.86) | 105.24(47.71-178.45) | 1.21(0.56-2.02) | 0.42*(0.4-0.44) |
| Myanmar | 168.59(76.76-287.18) | 1.30(0.61-2.19) | 337.84(167.53-575.19) | 1.40(0.69-2.38) | 0.23*(0.2-0.26) |
| Namibia | 4.70(2.15-8.20) | 1.19(0.56-2.05) | 11.99(5.66-20.50) | 1.29(0.62-2.20) | 0.29*(0.26-0.31) |
| Nauru | 0.06(0.03-0.09) | 1.63(0.92-2.59) | 0.07(0.04-0.11) | 1.77(1.00-2.78) | 0.27*(0.25-0.29) |
| Nepal | 72.76(32.41-123.74) | 1.21(0.55-2.04) | 188.64(89.68-318.32) | 1.59(0.76-2.66) | 0.88*(0.83-0.92) |
| Netherlands | 115.94(55.20-199.49) | 1.64(0.78-2.83) | 136.75(69.08-233.51) | 1.89(0.95-3.24) | 0.45*(0.42-0.49) |
| New Zealand | 8.36(3.35-14.62) | 0.56(0.22-0.99) | 15.69(6.88-26.83) | 0.71(0.31-1.21) | 0.74*(0.69-0.79) |
| Nicaragua | 20.21(9.68-33.48) | 2.02(0.99-3.29) | 67.38(36.21-109.82) | 2.48(1.34-4.02) | 0.66*(0.59-0.73) |
| Niger | 20.62(8.65-36.24) | 0.94(0.40-1.66) | 59.24(25.17-105.04) | 1.00(0.43-1.76) | 0.23*(0.15-0.3) |
| Nigeria | 273.20(131.49-448.36) | 1.01(0.49-1.66) | 771.26(372.33-1251.29) | 1.11(0.54-1.80) | 0.33*(0.29-0.37) |
| Niue | 0.01(0.01-0.02) | 1.48(0.73-2.43) | 0.01(0.01-0.02) | 1.58(0.80-2.64) | 0.22*(0.15-0.28) |
| North Macedonia | 8.38(3.88-14.54) | 1.00(0.46-1.73) | 11.75(5.42-20.34) | 1.03(0.47-1.79) | 0.08*(0.06-0.1) |
| Northern Mariana Islands | 0.30(0.15-0.51) | 1.44(0.71-2.40) | 0.37(0.18-0.61) | 1.49(0.73-2.49) | 0.12(-0.04-0.28) |
| Norway | 14.20(7.01-22.99) | 0.75(0.37-1.22) | 37.15(19.22-59.94) | 1.52(0.79-2.46) | 2.32*(2.26-2.37) |
| Oman | 8.75(4.15-14.92) | 1.31(0.65-2.18) | 43.98(22.25-75.65) | 1.71(0.88-2.93) | 0.84*(0.75-0.93) |
| Pakistan | 478.17(246.35-764.21) | 1.48(0.78-2.36) | 1508.97(808.14-2389.88) | 1.75(0.95-2.76) | 0.50*(0.44-0.57) |
| Palau | 0.10(0.05-0.17) | 1.69(0.89-2.76) | 0.19(0.10-0.31) | 1.81(0.94-2.97) | 0.21*(0.18-0.25) |
| Palestine | 5.93(2.88-10.28) | 1.25(0.64-2.12) | 27.59(13.84-46.09) | 1.57(0.81-2.60) | 0.73*(0.68-0.77) |
| Panama | 17.71(9.10-29.84) | 2.14(1.12-3.57) | 49.69(24.69-81.52) | 2.75(1.37-4.52) | 0.76*(0.59-0.93) |
| Papua New Guinea | 15.20(7.28-25.85) | 1.21(0.59-2.03) | 52.37(26.07-87.09) | 1.35(0.68-2.23) | 0.36*(0.31-0.42) |
| Paraguay | 23.39(11.44-39.27) | 1.87(0.94-3.10) | 71.20(37.77-114.88) | 2.43(1.30-3.90) | 0.87*(0.72-1.03) |
| Peru | 232.41(125.95-361.15) | 3.40(1.88-5.22) | 818.19(479.13-1232.07) | 5.17(3.03-7.77) | 1.37*(1.29-1.44) |
| Philippines | 266.69(138.64-424.97) | 1.32(0.70-2.09) | 678.24(363.33-1069.89) | 1.47(0.79-2.32) | 0.35*(0.32-0.38) |
| Poland | 212.77(106.01-347.54) | 1.32(0.65-2.17) | 193.34(123.94-271.69) | 0.98(0.63-1.39) | -0.96*(-1.02--0.9) |
| Portugal | 56.77(28.02-96.58) | 1.35(0.67-2.30) | 86.63(44.99-145.42) | 1.70(0.86-2.87) | 0.72*(0.65-0.79) |
| Puerto Rico | 29.75(15.83-48.15) | 1.98(1.05-3.21) | 32.45(17.70-52.78) | 2.27(1.22-3.73) | 0.44*(0.38-0.5) |
| Qatar | 3.16(1.50-5.40) | 1.43(0.70-2.40) | 33.99(16.13-58.64) | 1.66(0.80-2.83) | 0.48*(0.43-0.53) |
| Republic of Korea | 170.37(71.77-305.32) | 0.92(0.39-1.63) | 325.77(144.98-549.87) | 1.20(0.53-2.05) | 0.90*(0.83-0.96) |
| Republic of Moldova | 23.65(10.57-41.35) | 1.36(0.62-2.36) | 23.35(10.03-39.83) | 1.23(0.52-2.11) | -0.31*(-0.37--0.24) |
| Romania | 86.55(39.00-151.22) | 0.92(0.41-1.61) | 96.16(42.93-166.07) | 1.01(0.44-1.77) | 0.28*(0.23-0.34) |
| Russian Federation | 787.89(393.06-1277.24) | 1.33(0.66-2.15) | 955.67(486.71-1530.46) | 1.33(0.67-2.14) | 0.01(-0.07-0.09) |
| Rwanda | 23.76(10.79-41.23) | 1.29(0.61-2.20) | 54.88(24.67-94.97) | 1.19(0.54-2.05) | -0.27*(-0.3--0.25) |
| Saint Kitts and Nevis | 0.24(0.12-0.40) | 2.05(1.07-3.39) | 0.70(0.38-1.14) | 2.31(1.25-3.77) | 0.34*(0.27-0.41) |
| Saint Lucia | 0.81(0.41-1.33) | 2.02(1.03-3.26) | 2.03(1.12-3.32) | 2.28(1.24-3.73) | 0.36*(0.23-0.49) |
| Saint Vincent and the Grenadines | 0.57(0.28-0.96) | 1.83(0.93-3.04) | 1.08(0.57-1.75) | 2.10(1.11-3.43) | 0.40*(0.31-0.5) |
| Samoa | 0.59(0.28-1.01) | 1.33(0.65-2.24) | 1.07(0.54-1.80) | 1.48(0.75-2.47) | 0.38*(0.35-0.41) |
| San Marino | 0.16(0.08-0.28) | 1.52(0.71-2.69) | 0.25(0.12-0.43) | 1.67(0.77-2.87) | 0.29*(0.25-0.34) |
| Sao Tome and Principe | 0.32(0.13-0.56) | 1.09(0.46-1.88) | 1.09(0.51-1.87) | 1.34(0.63-2.29) | 0.70*(0.65-0.75) |
| Saudi Arabia | 65.85(31.42-111.19) | 1.27(0.62-2.12) | 468.05(258.38-758.90) | 1.96(1.08-3.19) | 1.40*(1.38-1.43) |
| Senegal | 18.75(7.77-33.67) | 0.93(0.39-1.66) | 55.26(24.03-96.83) | 1.09(0.48-1.90) | 0.56*(0.47-0.65) |
| Serbia | 38.87(16.10-69.74) | 0.93(0.39-1.66) | 39.40(17.33-69.28) | 0.91(0.40-1.62) | -0.01(-0.08-0.05) |
| Seychelles | 0.36(0.17-0.63) | 1.55(0.73-2.64) | 0.91(0.44-1.53) | 1.72(0.82-2.89) | 0.33*(0.31-0.36) |
| Sierra Leone | 10.86(4.40-19.30) | 0.88(0.36-1.55) | 28.87(12.56-50.92) | 1.01(0.44-1.78) | 0.49*(0.46-0.52) |
| Singapore | 15.34(6.59-26.95) | 1.04(0.45-1.82) | 40.10(18.74-68.12) | 1.18(0.53-2.05) | 0.42*(0.39-0.46) |
| Slovakia | 20.65(9.21-36.28) | 0.93(0.41-1.63) | 26.86(11.91-46.49) | 0.93(0.40-1.62) | -0.01(-0.05-0.04) |
| Slovenia | 9.07(4.14-16.14) | 1.02(0.47-1.81) | 10.52(4.47-18.59) | 1.04(0.43-1.85) | 0.06*(0.01-0.1) |
| Solomon Islands | 1.38(0.75-2.25) | 1.58(0.88-2.53) | 4.44(2.54-6.95) | 1.76(1.01-2.75) | 0.35*(0.31-0.4) |
| Somalia | 27.62(12.88-48.46) | 1.19(0.56-2.08) | 76.83(35.45-133.76) | 1.23(0.57-2.14) | 0.11*(0.09-0.12) |
| South Africa | 149.31(77.22-238.05) | 1.21(0.63-1.91) | 326.07(166.91-523.66) | 1.27(0.65-2.03) | 0.16*(0.14-0.18) |
| South Sudan | 17.16(7.49-30.56) | 1.08(0.48-1.91) | 33.26(14.13-57.26) | 1.10(0.47-1.89) | 0.07*(0.06-0.09) |
| Spain | 234.88(111.17-398.74) | 1.47(0.69-2.49) | 446.49(211.43-786.43) | 1.89(0.89-3.36) | 0.83*(0.77-0.89) |
| Sri Lanka | 85.27(38.99-149.09) | 1.28(0.59-2.22) | 144.26(67.45-253.31) | 1.45(0.67-2.55) | 0.42*(0.38-0.45) |
| Sudan | 78.87(41.45-129.56) | 1.41(0.76-2.28) | 247.80(129.96-396.27) | 1.71(0.92-2.70) | 0.62*(0.59-0.66) |
| Suriname | 2.15(1.05-3.57) | 1.65(0.82-2.70) | 4.93(2.60-8.16) | 1.96(1.03-3.25) | 0.56*(0.49-0.62) |
| Sweden | 60.86(31.79-97.15) | 1.49(0.77-2.39) | 72.71(38.12-116.47) | 1.65(0.87-2.65) | 0.33*(0.32-0.34) |
| Switzerland | 53.48(25.50-92.67) | 1.57(0.75-2.73) | 72.95(35.69-125.61) | 1.77(0.86-3.06) | 0.38*(0.36-0.4) |
| Syrian Arab Republic | 34.94(16.10-60.70) | 1.11(0.53-1.89) | 95.78(49.48-160.20) | 1.47(0.74-2.49) | 0.91*(0.88-0.95) |
| Taiwan (Province of China) | 98.06(51.24-156.65) | 1.24(0.67-1.95) | 223.85(137.84-325.59) | 1.78(1.08-2.61) | 1.17*(1.1-1.25) |
| Tajikistan | 13.30(5.48-24.63) | 1.03(0.45-1.84) | 38.34(16.46-68.55) | 1.03(0.45-1.82) | -0.02(-0.04-0) |
| Thailand | 294.73(142.87-497.40) | 1.34(0.66-2.25) | 498.87(237.30-819.90) | 1.45(0.69-2.40) | 0.27*(0.25-0.28) |
| Timor-Leste | 3.00(1.34-5.20) | 1.19(0.55-2.04) | 5.65(2.52-9.95) | 1.28(0.59-2.24) | 0.26*(0.23-0.28) |
| Togo | 8.99(3.68-15.67) | 0.92(0.38-1.59) | 31.90(13.99-54.99) | 1.07(0.47-1.84) | 0.52*(0.46-0.59) |
| Tokelau | 0.01(0.00-0.01) | 1.37(0.70-2.29) | 0.01(0.00-0.01) | 1.44(0.68-2.40) | 0.18*(0.07-0.28) |
| Tonga | 0.33(0.15-0.59) | 1.21(0.57-2.12) | 0.51(0.24-0.87) | 1.38(0.65-2.36) | 0.43*(0.36-0.49) |
| Trinidad and Tobago | 8.28(4.14-13.63) | 1.87(0.95-3.05) | 14.06(7.37-22.61) | 2.10(1.09-3.40) | 0.31*(0.17-0.46) |
| Tunisia | 29.57(14.06-50.51) | 1.16(0.57-1.95) | 93.30(49.45-154.77) | 1.61(0.84-2.68) | 1.04*(1.01-1.07) |
| Türkiye | 248.52(124.65-419.62) | 1.30(0.67-2.17) | 656.94(333.23-1094.78) | 1.60(0.80-2.69) | 0.67*(0.64-0.7) |
| Turkmenistan | 9.96(4.02-18.06) | 1.01(0.43-1.77) | 23.19(10.49-40.61) | 1.07(0.49-1.87) | 0.18*(0.14-0.22) |
| Tuvalu | 0.05(0.03-0.08) | 1.46(0.78-2.34) | 0.07(0.04-0.13) | 1.58(0.82-2.70) | 0.27*(0.24-0.31) |
| Uganda | 48.79(21.02-85.46) | 1.15(0.52-1.98) | 147.79(67.24-257.77) | 1.26(0.59-2.18) | 0.30*(0.28-0.32) |
| Ukraine | 326.42(168.26-522.79) | 1.51(0.78-2.42) | 348.88(187.61-546.18) | 1.57(0.84-2.48) | 0.13*(0.1-0.15) |
| United Arab Emirates | 14.71(7.62-23.96) | 1.69(0.91-2.69) | 226.53(128.02-361.68) | 2.39(1.32-3.86) | 1.12*(1.09-1.14) |
| United Kingdom | 502.76(273.19-784.04) | 1.98(1.07-3.09) | 590.30(316.12-928.16) | 2.01(1.07-3.16) | 0.09*(0.02-0.15) |
| United Republic of Tanzania | 74.59(32.18-131.93) | 1.11(0.49-1.93) | 227.21(105.00-388.69) | 1.23(0.57-2.08) | 0.32*(0.29-0.36) |
| United States of America | 1559.12(795.08-2497.61) | 1.35(0.69-2.17) | 1520.95(1071.53-2051.98) | 1.07(0.75-1.45) | -0.75*(-0.77--0.72) |
| United States Virgin Islands | 1.22(0.68-1.96) | 2.40(1.32-3.91) | 1.03(0.62-1.61) | 2.87(1.69-4.52) | 0.57*(0.56-0.59) |
| Uruguay | 10.85(4.56-19.14) | 0.88(0.37-1.55) | 17.74(7.83-30.74) | 1.17(0.51-2.03) | 0.94*(0.84-1.04) |
| Uzbekistan | 60.03(25.08-108.42) | 1.09(0.49-1.91) | 154.91(69.65-273.98) | 1.04(0.47-1.83) | -0.16*(-0.23--0.09) |
| Vanuatu | 0.65(0.33-1.06) | 1.46(0.76-2.35) | 1.85(0.99-2.98) | 1.64(0.89-2.63) | 0.38*(0.36-0.4) |
| Venezuela | 120.28(58.21-203.14) | 1.93(0.96-3.22) | 312.04(164.08-503.17) | 2.45(1.27-3.99) | 0.78*(0.6-0.96) |
| Viet Nam | 261.12(120.11-452.18) | 1.37(0.66-2.32) | 838.87(412.21-1400.34) | 1.74(0.85-2.91) | 0.76*(0.73-0.78) |
| Yemen | 39.37(19.66-65.51) | 1.19(0.61-1.94) | 170.52(90.90-271.57) | 1.53(0.84-2.42) | 0.83*(0.79-0.87) |
| Zambia | 24.35(11.27-42.22) | 1.22(0.58-2.09) | 80.92(36.22-137.24) | 1.32(0.61-2.22) | 0.24*(0.21-0.27) |
| Zimbabwe | 31.14(13.80-53.96) | 1.17(0.53-2.00) | 66.67(30.20-115.10) | 1.26(0.57-2.16) | 0.23*(0.19-0.28) |

Note: UI: uncertainty interval; CI, confidence interval; AAPC, average annual percent of change; ASIR, age-standardized incidence rate

Table S3. The case number and age-standardized rate of death among 204 countries and territories, with the AAPCs between 1990 and 2021

|  | 1990 | | 2021 | |  |
| --- | --- | --- | --- | --- | --- |
|  | Number (95%UI) | ASMR per 100,000 populations (95%UI) | Number (95%UI) | ASMR per 100,000 populations (95%UI) | AAPCs (95%CI) |
| Afghanistan | 5.76(2.17-9.76) | 0.18(0.07-0.31) | 16.00(7.39-28.13) | 0.16(0.07-0.28) | -0.37*(-0.45--0.28) |
| Albania | 0.43(0.34-0.55) | 0.04(0.03-0.05) | 0.36(0.28-0.46) | 0.03(0.02-0.04) | -0.71(-1.66-0.25) |
| Algeria | 4.21(3.13-5.67) | 0.07(0.05-0.09) | 12.44(8.83-16.87) | 0.06(0.05-0.09) | -0.37*(-0.47--0.27) |
| American Samoa | 0.01(0.01-0.01) | 0.07(0.05-0.09) | 0.01(0.01-0.02) | 0.06(0.04-0.08) | -0.45*(-0.62--0.29) |
| Andorra | 0.01(0.01-0.01) | 0.03(0.02-0.05) | 0.01(0.01-0.02) | 0.02(0.02-0.03) | -1.29*(-1.66--0.92) |
| Angola | 1.80(1.19-2.49) | 0.07(0.04-0.09) | 5.34(3.82-7.29) | 0.06(0.04-0.08) | -0.31(-0.81-0.2) |
| Antigua and Barbuda | 0.01(0.01-0.01) | 0.06(0.05-0.06) | 0.02(0.02-0.02) | 0.05(0.04-0.05) | 0.08(-0.22-0.38) |
| Argentina | 5.35(4.86-5.86) | 0.04(0.04-0.05) | 7.34(6.62-8.11) | 0.03(0.03-0.04) | -0.55*(-0.9--0.2) |
| Armenia | 0.41(0.37-0.46) | 0.04(0.04-0.04) | 0.36(0.30-0.42) | 0.03(0.02-0.03) | -1.27*(-2.14--0.41) |
| Australia | 2.37(2.17-2.59) | 0.03(0.03-0.03) | 4.41(3.98-4.85) | 0.04(0.03-0.04) | 0.53*(0.29-0.77) |
| Austria | 1.49(1.37-1.63) | 0.04(0.04-0.04) | 1.13(1.03-1.24) | 0.03(0.02-0.03) | -1.52*(-1.93--1.12) |
| Azerbaijan | 1.02(0.75-1.31) | 0.05(0.04-0.06) | 1.47(1.11-1.95) | 0.03(0.02-0.04) | -1.62*(-2.02--1.21) |
| Bahamas | 0.08(0.07-0.09) | 0.09(0.07-0.10) | 0.18(0.14-0.24) | 0.09(0.07-0.12) | 0.33(-0.72-1.39) |
| Bahrain | 0.10(0.08-0.13) | 0.07(0.05-0.08) | 0.42(0.31-0.55) | 0.04(0.03-0.06) | -1.44*(-2.03--0.85) |
| Bangladesh | 16.77(12.26-22.24) | 0.06(0.04-0.08) | 29.46(18.94-46.87) | 0.04(0.03-0.07) | -1.07*(-1.23--0.91) |
| Barbados | 0.05(0.04-0.06) | 0.05(0.05-0.06) | 0.07(0.05-0.09) | 0.05(0.04-0.06) | -0.3(-0.9-0.3) |
| Belarus | 1.43(1.26-1.64) | 0.04(0.03-0.04) | 1.75(1.36-2.17) | 0.04(0.03-0.05) | 0.15(-0.49-0.79) |
| Belgium | 1.47(1.35-1.62) | 0.04(0.03-0.04) | 1.69(1.54-1.84) | 0.03(0.03-0.03) | -0.45*(-0.65--0.24) |
| Belize | 0.02(0.02-0.02) | 0.05(0.04-0.05) | 0.13(0.11-0.16) | 0.08(0.06-0.09) | 1.75*(1.01-2.49) |
| Benin | 0.83(0.61-1.11) | 0.08(0.06-0.10) | 2.98(1.92-4.56) | 0.08(0.05-0.12) | 0.12(-0.03-0.27) |
| Bermuda | 0.02(0.02-0.02) | 0.06(0.05-0.08) | 0.01(0.01-0.02) | 0.04(0.02-0.06) | -1.87*(-2.05--1.69) |
| Bhutan | 0.10(0.06-0.14) | 0.07(0.04-0.09) | 0.18(0.12-0.25) | 0.06(0.04-0.08) | -0.56*(-0.65--0.46) |
| Bolivia | 1.60(1.14-2.08) | 0.09(0.06-0.11) | 2.99(2.03-4.28) | 0.06(0.04-0.09) | -0.99*(-1.06--0.92) |
| Bosnia and Herzegovina | 0.86(0.69-1.10) | 0.05(0.04-0.06) | 0.58(0.40-0.76) | 0.04(0.02-0.05) | -1.17*(-1.77--0.57) |
| Botswana | 0.22(0.15-0.33) | 0.07(0.05-0.10) | 0.53(0.35-0.78) | 0.05(0.03-0.07) | -0.93*(-1.74--0.11) |
| Brazil | 25.05(24.18-25.98) | 0.05(0.05-0.05) | 55.61(53.54-57.86) | 0.05(0.05-0.05) | 0.05(-0.2-0.29) |
| Brunei Darussalam | 0.06(0.04-0.09) | 0.08(0.05-0.11) | 0.19(0.11-0.25) | 0.08(0.05-0.11) | 0.26(-0.18-0.71) |
| Bulgaria | 2.10(1.91-2.31) | 0.05(0.05-0.06) | 2.18(1.80-2.60) | 0.06(0.05-0.07) | 0.58*(0.31-0.84) |
| Burkina Faso | 1.67(1.12-2.34) | 0.07(0.05-0.10) | 4.92(2.98-7.49) | 0.08(0.05-0.12) | 0.38*(0.17-0.59) |
| Burundi | 0.93(0.58-1.39) | 0.07(0.05-0.11) | 1.90(1.12-2.85) | 0.06(0.03-0.08) | -0.90*(-1.17--0.63) |
| Cabo Verde | 0.05(0.03-0.06) | 0.08(0.06-0.10) | 0.23(0.16-0.33) | 0.10(0.07-0.14) | 0.83*(0.44-1.21) |
| Cambodia | 2.66(1.89-3.54) | 0.10(0.07-0.13) | 5.71(4.03-8.15) | 0.09(0.06-0.13) | -0.33*(-0.39--0.28) |
| Cameroon | 2.51(1.86-3.32) | 0.10(0.07-0.13) | 10.50(6.12-17.02) | 0.11(0.07-0.18) | 0.53*(0.42-0.63) |
| Canada | 5.23(4.84-5.67) | 0.04(0.04-0.05) | 8.67(7.89-9.54) | 0.05(0.05-0.06) | 0.59*(0.4-0.78) |
| Central African Republic | 0.64(0.36-0.92) | 0.09(0.05-0.13) | 1.39(0.77-2.21) | 0.08(0.05-0.13) | -0.31*(-0.58--0.04) |
| Chad | 0.84(0.61-1.16) | 0.06(0.04-0.08) | 3.57(2.38-5.16) | 0.09(0.06-0.13) | 1.28*(1.05-1.5) |
| Chile | 2.52(2.27-2.80) | 0.05(0.05-0.06) | 3.24(2.92-3.60) | 0.04(0.03-0.04) | -1.01*(-1.65--0.36) |
| China | 493.79(392.90-575.37) | 0.12(0.09-0.14) | 561.91(444.46-705.74) | 0.08(0.06-0.10) | -1.41*(-1.52--1.3) |
| Colombia | 6.44(5.79-7.19) | 0.06(0.06-0.07) | 9.80(7.88-12.11) | 0.05(0.04-0.06) | -1.01*(-1.39--0.64) |
| Comoros | 0.08(0.05-0.11) | 0.07(0.04-0.09) | 0.16(0.11-0.23) | 0.06(0.04-0.08) | -0.46(-1.04-0.11) |
| Congo | 0.56(0.33-0.78) | 0.10(0.06-0.14) | 1.45(0.96-2.12) | 0.07(0.05-0.10) | -1.08*(-1.49--0.66) |
| Cook Islands | 0.00(0.00-0.01) | 0.06(0.04-0.09) | 0.00(0.00-0.00) | 0.04(0.03-0.06) | -1.17*(-1.37--0.97) |
| Costa Rica | 0.40(0.36-0.44) | 0.04(0.04-0.05) | 1.16(0.99-1.33) | 0.05(0.05-0.06) | 0.52*(0.13-0.92) |
| Côte d'Ivoire | 3.01(2.26-3.87) | 0.10(0.07-0.13) | 9.67(6.04-15.10) | 0.11(0.07-0.17) | 0.23(-0.06-0.53) |
| Croatia | 0.95(0.86-1.05) | 0.04(0.04-0.05) | 0.59(0.49-0.69) | 0.03(0.02-0.03) | -1.44*(-1.62--1.25) |
| Cuba | 2.00(1.81-2.20) | 0.04(0.04-0.05) | 2.69(2.23-3.17) | 0.05(0.04-0.06) | 0.26(-0.3-0.82) |
| Cyprus | 0.12(0.09-0.15) | 0.04(0.03-0.04) | 0.14(0.11-0.19) | 0.02(0.02-0.03) | -1.64*(-2.01--1.26) |
| Czechia | 2.28(2.09-2.50) | 0.05(0.04-0.05) | 1.67(1.39-1.97) | 0.03(0.02-0.03) | -1.71*(-2.14--1.27) |
| Democratic People's Republic of Korea | 11.93(7.74-17.47) | 0.14(0.09-0.20) | 19.36(12.31-29.43) | 0.14(0.09-0.22) | 0.16*(0.05-0.26) |
| Democratic Republic of the Congo | 5.33(3.57-7.27) | 0.06(0.04-0.08) | 14.53(9.47-20.81) | 0.06(0.04-0.08) | -0.15(-0.32-0.03) |
| Denmark | 0.83(0.75-0.91) | 0.03(0.03-0.03) | 0.60(0.55-0.65) | 0.02(0.02-0.02) | -1.14*(-1.35--0.94) |
| Djibouti | 0.05(0.04-0.08) | 0.05(0.03-0.07) | 0.26(0.15-0.43) | 0.05(0.03-0.08) | 0.09(-0.06-0.24) |
| Dominica | 0.01(0.01-0.02) | 0.07(0.05-0.09) | 0.02(0.02-0.03) | 0.08(0.05-0.10) | 0.37*(0.17-0.56) |
| Dominican Republic | 1.27(1.00-1.60) | 0.06(0.05-0.08) | 3.36(2.49-4.44) | 0.07(0.05-0.10) | 0.56(-0.2-1.32) |
| Ecuador | 1.61(1.46-1.77) | 0.05(0.05-0.06) | 3.70(2.74-4.82) | 0.05(0.04-0.07) | -0.08(-0.61-0.45) |
| Egypt | 15.67(12.96-18.80) | 0.09(0.07-0.11) | 23.26(17.58-30.53) | 0.06(0.05-0.08) | -1.31*(-1.73--0.88) |
| El Salvador | 1.30(1.07-1.58) | 0.09(0.07-0.11) | 2.36(1.72-3.16) | 0.09(0.07-0.12) | 0.13(-0.79-1.06) |
| Equatorial Guinea | 0.09(0.05-0.13) | 0.08(0.05-0.12) | 0.25(0.16-0.40) | 0.06(0.04-0.10) | -0.76*(-1.02--0.5) |
| Eritrea | 0.76(0.49-1.08) | 0.09(0.05-0.12) | 1.74(1.14-2.61) | 0.08(0.05-0.12) | -0.26*(-0.45--0.07) |
| Estonia | 0.25(0.21-0.33) | 0.04(0.03-0.05) | 0.14(0.12-0.17) | 0.02(0.02-0.03) | -1.76*(-2.66--0.85) |
| Eswatini | 0.16(0.10-0.23) | 0.08(0.05-0.11) | 0.49(0.26-0.76) | 0.13(0.07-0.20) | 1.51*(1.23-1.78) |
| Ethiopia | 9.80(6.98-13.75) | 0.08(0.06-0.11) | 12.67(8.48-20.08) | 0.04(0.03-0.07) | -2.07*(-2.23--1.92) |
| Fiji | 0.17(0.13-0.22) | 0.07(0.05-0.09) | 0.21(0.15-0.28) | 0.05(0.04-0.07) | -0.65*(-0.99--0.32) |
| Finland | 1.16(1.05-1.29) | 0.05(0.04-0.05) | 0.74(0.67-0.81) | 0.03(0.03-0.03) | -1.07*(-1.23--0.91) |
| France | 10.26(9.23-11.36) | 0.04(0.04-0.05) | 10.73(9.72-11.83) | 0.04(0.03-0.04) | -0.59*(-0.86--0.33) |
| Gabon | 0.22(0.14-0.31) | 0.10(0.06-0.13) | 0.47(0.31-0.70) | 0.08(0.05-0.11) | -0.66*(-0.9--0.42) |
| Gambia | 0.17(0.10-0.27) | 0.07(0.04-0.11) | 0.60(0.36-1.02) | 0.09(0.05-0.15) | 0.73(-0.29-1.77) |
| Georgia | 1.01(0.88-1.17) | 0.05(0.04-0.06) | 0.75(0.63-0.86) | 0.05(0.04-0.05) | -0.29(-0.91-0.35) |
| Germany | 14.83(13.49-16.23) | 0.04(0.04-0.04) | 11.68(10.66-12.78) | 0.03(0.03-0.04) | -0.77*(-0.95--0.6) |
| Ghana | 2.60(1.81-3.56) | 0.07(0.05-0.09) | 8.56(5.78-12.35) | 0.07(0.05-0.11) | 0.35*(0.26-0.44) |
| Greece | 1.18(1.07-1.31) | 0.03(0.02-0.03) | 1.88(1.72-2.06) | 0.03(0.03-0.04) | 0.88*(0.73-1.03) |
| Greenland | 0.03(0.02-0.04) | 0.13(0.07-0.17) | 0.02(0.01-0.03) | 0.09(0.05-0.12) | -1.01*(-1.06--0.95) |
| Grenada | 0.01(0.01-0.02) | 0.06(0.05-0.07) | 0.03(0.03-0.04) | 0.07(0.05-0.08) | 0.33(-0.14-0.81) |
| Guam | 0.02(0.02-0.03) | 0.04(0.03-0.06) | 0.03(0.02-0.04) | 0.05(0.03-0.06) | -0.01(-0.85-0.83) |
| Guatemala | 1.69(1.53-1.86) | 0.08(0.07-0.09) | 3.72(3.07-4.49) | 0.07(0.05-0.08) | -0.64(-1.45-0.18) |
| Guinea | 1.08(0.73-1.54) | 0.06(0.04-0.09) | 2.84(1.79-4.51) | 0.08(0.05-0.13) | 0.71*(0.58-0.84) |
| Guinea-Bissau | 0.34(0.20-0.49) | 0.14(0.08-0.21) | 0.83(0.52-1.20) | 0.14(0.09-0.21) | 0.1(0-0.2) |
| Guyana | 0.17(0.14-0.19) | 0.07(0.06-0.08) | 0.31(0.23-0.41) | 0.10(0.07-0.13) | 1.03*(0.63-1.43) |
| Haiti | 1.95(1.06-2.77) | 0.11(0.06-0.15) | 4.35(2.59-6.44) | 0.09(0.05-0.13) | -0.38*(-0.48--0.27) |
| Honduras | 1.58(1.25-1.96) | 0.13(0.11-0.17) | 4.12(2.64-6.01) | 0.11(0.07-0.17) | -0.50*(-0.65--0.35) |
| Hungary | 2.93(2.63-3.24) | 0.06(0.05-0.07) | 1.99(1.66-2.33) | 0.04(0.03-0.04) | -1.56*(-2.35--0.76) |
| Iceland | 0.04(0.04-0.05) | 0.04(0.04-0.05) | 0.07(0.06-0.07) | 0.04(0.04-0.05) | -0.15(-0.86-0.57) |
| India | 164.76(131.60-216.02) | 0.06(0.05-0.08) | 367.36(294.36-446.62) | 0.06(0.05-0.07) | 0.15*(0.05-0.25) |
| Indonesia | 50.97(41.10-61.36) | 0.09(0.07-0.10) | 114.27(87.87-152.84) | 0.08(0.06-0.11) | -0.15*(-0.21--0.09) |
| Iran (Islamic Republic of) | 8.47(5.32-10.23) | 0.07(0.04-0.08) | 21.97(11.62-25.76) | 0.05(0.03-0.06) | -0.89*(-1.06--0.72) |
| Iraq | 5.76(4.22-7.66) | 0.13(0.09-0.17) | 16.89(12.09-23.94) | 0.10(0.07-0.14) | -0.83*(-0.92--0.74) |
| Ireland | 0.44(0.40-0.48) | 0.03(0.03-0.03) | 0.63(0.58-0.70) | 0.02(0.02-0.03) | -0.79(-1.73-0.15) |
| Israel | 0.50(0.45-0.54) | 0.03(0.03-0.03) | 0.98(0.88-1.09) | 0.02(0.02-0.03) | -0.65*(-1.1--0.19) |
| Italy | 10.53(10.18-10.88) | 0.04(0.04-0.04) | 9.76(9.40-10.13) | 0.03(0.03-0.03) | -1.22*(-1.61--0.83) |
| Jamaica | 0.23(0.21-0.25) | 0.03(0.03-0.04) | 0.64(0.46-0.88) | 0.05(0.04-0.07) | 2.24*(0.76-3.74) |
| Japan | 26.26(25.60-26.90) | 0.04(0.04-0.04) | 26.71(26.00-27.37) | 0.04(0.04-0.04) | 0.13(-0.42-0.68) |
| Jordan | 0.80(0.58-1.05) | 0.09(0.07-0.12) | 2.63(1.97-3.43) | 0.05(0.04-0.07) | -1.80*(-2.08--1.53) |
| Kazakhstan | 3.15(2.74-3.63) | 0.06(0.05-0.07) | 4.60(3.80-5.51) | 0.05(0.04-0.07) | -0.26(-0.7-0.19) |
| Kenya | 1.87(1.46-2.48) | 0.04(0.03-0.05) | 7.81(6.02-10.13) | 0.05(0.04-0.06) | 0.91*(0.77-1.05) |
| Kiribati | 0.02(0.01-0.02) | 0.08(0.06-0.10) | 0.03(0.02-0.04) | 0.07(0.05-0.10) | -0.22*(-0.28--0.16) |
| Kuwait | 0.65(0.57-0.73) | 0.11(0.10-0.12) | 1.08(0.86-1.36) | 0.03(0.03-0.04) | -3.60*(-4.7--2.47) |
| Kyrgyzstan | 0.61(0.49-0.77) | 0.05(0.04-0.07) | 1.08(0.87-1.34) | 0.04(0.03-0.05) | -0.97*(-1.41--0.54) |
| Lao People's Democratic Republic | 1.36(0.81-2.00) | 0.12(0.07-0.18) | 2.55(1.78-3.52) | 0.09(0.06-0.12) | -0.94*(-1.04--0.85) |
| Latvia | 0.48(0.42-0.58) | 0.04(0.03-0.05) | 0.30(0.26-0.35) | 0.03(0.03-0.04) | -0.59(-1.95-0.79) |
| Lebanon | 0.98(0.49-1.48) | 0.10(0.05-0.14) | 1.14(0.86-1.44) | 0.05(0.04-0.06) | -2.27*(-2.43--2.11) |
| Lesotho | 0.20(0.14-0.28) | 0.05(0.03-0.06) | 0.56(0.34-0.80) | 0.09(0.06-0.13) | 2.29*(1.93-2.64) |
| Liberia | 0.43(0.30-0.65) | 0.08(0.05-0.11) | 1.80(0.99-3.24) | 0.09(0.05-0.16) | 0.46*(0.15-0.78) |
| Libya | 0.75(0.54-1.09) | 0.07(0.05-0.10) | 4.44(2.78-7.51) | 0.11(0.07-0.19) | 1.50*(1.29-1.72) |
| Lithuania | 0.58(0.50-0.72) | 0.04(0.03-0.05) | 0.42(0.36-0.48) | 0.03(0.03-0.04) | -0.54(-1.5-0.43) |
| Luxembourg | 0.08(0.07-0.09) | 0.05(0.04-0.05) | 0.08(0.07-0.09) | 0.02(0.02-0.03) | -2.21*(-2.63--1.79) |
| Madagascar | 1.40(1.05-1.79) | 0.05(0.04-0.06) | 3.84(2.60-5.47) | 0.04(0.03-0.06) | -0.35*(-0.6--0.09) |
| Malawi | 1.25(0.91-1.61) | 0.05(0.04-0.07) | 3.17(2.07-4.41) | 0.06(0.04-0.08) | 0.44*(0.29-0.59) |
| Malaysia | 3.18(2.62-3.82) | 0.06(0.05-0.07) | 9.69(7.77-11.62) | 0.07(0.06-0.09) | 0.77*(0.34-1.19) |
| Maldives | 0.04(0.02-0.06) | 0.08(0.04-0.13) | 0.11(0.08-0.16) | 0.04(0.03-0.06) | -2.42*(-2.6--2.24) |
| Mali | 1.87(1.35-2.49) | 0.08(0.06-0.11) | 4.66(3.05-7.12) | 0.08(0.05-0.12) | -0.02(-0.16-0.13) |
| Malta | 0.05(0.05-0.06) | 0.03(0.03-0.03) | 0.08(0.07-0.09) | 0.04(0.03-0.04) | 0.76*(0.28-1.23) |
| Marshall Islands | 0.01(0.01-0.01) | 0.09(0.07-0.11) | 0.02(0.01-0.03) | 0.09(0.06-0.13) | 0.07*(0.01-0.14) |
| Mauritania | 0.34(0.23-0.47) | 0.07(0.04-0.09) | 0.68(0.38-1.28) | 0.05(0.03-0.10) | -0.63*(-0.99--0.27) |
| Mauritius | 0.30(0.27-0.33) | 0.08(0.07-0.09) | 0.52(0.45-0.59) | 0.08(0.07-0.09) | 0.18(-0.35-0.71) |
| Mexico | 19.56(19.01-20.13) | 0.08(0.08-0.08) | 45.40(39.73-51.45) | 0.08(0.07-0.09) | -0.06(-0.38-0.27) |
| Micronesia (Federated States of) | 0.03(0.02-0.04) | 0.10(0.06-0.13) | 0.03(0.02-0.05) | 0.08(0.05-0.11) | -0.54*(-0.58--0.5) |
| Monaco | 0.01(0.00-0.01) | 0.04(0.03-0.05) | 0.01(0.00-0.01) | 0.05(0.03-0.07) | 0.35*(0.28-0.41) |
| Mongolia | 0.29(0.23-0.36) | 0.05(0.04-0.06) | 0.74(0.59-0.90) | 0.05(0.04-0.06) | -0.17(-0.96-0.63) |
| Montenegro | 0.12(0.10-0.15) | 0.05(0.04-0.06) | 0.12(0.10-0.16) | 0.04(0.03-0.05) | -0.68*(-1.19--0.16) |
| Morocco | 5.59(4.05-7.68) | 0.08(0.06-0.11) | 14.85(9.48-22.73) | 0.09(0.06-0.14) | 0.46*(0.38-0.53) |
| Mozambique | 1.97(1.37-2.72) | 0.05(0.04-0.07) | 5.93(3.74-8.81) | 0.07(0.05-0.11) | 1.09*(0.95-1.22) |
| Myanmar | 12.25(7.43-17.65) | 0.10(0.06-0.15) | 21.80(15.47-30.40) | 0.09(0.06-0.12) | -0.53*(-0.61--0.45) |
| Namibia | 0.24(0.18-0.32) | 0.07(0.05-0.09) | 0.72(0.45-1.05) | 0.08(0.05-0.12) | 0.72*(0.38-1.06) |
| Nauru | 0.00(0.00-0.01) | 0.13(0.07-0.19) | 0.00(0.00-0.01) | 0.11(0.06-0.17) | -0.57*(-0.62--0.52) |
| Nepal | 3.13(2.03-4.42) | 0.06(0.04-0.08) | 6.42(4.55-8.75) | 0.06(0.04-0.08) | 0.06(-0.07-0.2) |
| Netherlands | 2.30(2.11-2.51) | 0.03(0.03-0.04) | 2.15(1.97-2.34) | 0.03(0.03-0.03) | -0.57*(-0.82--0.31) |
| New Zealand | 0.63(0.58-0.69) | 0.04(0.04-0.05) | 1.05(0.97-1.15) | 0.05(0.04-0.05) | 0.23(-0.5-0.97) |
| Nicaragua | 0.53(0.44-0.64) | 0.06(0.05-0.07) | 1.33(1.05-1.69) | 0.05(0.04-0.06) | -0.75*(-1.18--0.33) |
| Niger | 1.44(1.00-2.00) | 0.07(0.05-0.10) | 3.66(2.22-5.87) | 0.07(0.04-0.11) | -0.09(-0.38-0.2) |
| Nigeria | 20.59(14.39-28.01) | 0.08(0.06-0.11) | 48.97(29.18-86.89) | 0.08(0.05-0.14) | -0.35*(-0.51--0.19) |
| Niue | 0.00(0.00-0.00) | 0.08(0.05-0.11) | 0.00(0.00-0.00) | 0.06(0.04-0.09) | -0.80*(-1.04--0.56) |
| North Macedonia | 0.37(0.30-0.46) | 0.04(0.04-0.05) | 0.36(0.26-0.47) | 0.03(0.02-0.04) | -1.29*(-1.64--0.95) |
| Northern Mariana Islands | 0.01(0.01-0.02) | 0.07(0.04-0.10) | 0.01(0.01-0.02) | 0.05(0.03-0.06) | -0.97*(-1.34--0.59) |
| Norway | 0.63(0.60-0.65) | 0.03(0.03-0.03) | 0.78(0.74-0.82) | 0.03(0.03-0.03) | -0.19(-0.63-0.25) |
| Oman | 0.54(0.38-0.75) | 0.10(0.07-0.13) | 1.29(0.93-1.78) | 0.06(0.04-0.08) | -1.76*(-2.09--1.42) |
| Pakistan | 17.60(13.42-21.32) | 0.06(0.04-0.07) | 56.88(41.71-75.10) | 0.07(0.05-0.09) | 0.67*(0.6-0.73) |
| Palau | 0.00(0.00-0.01) | 0.07(0.05-0.09) | 0.01(0.00-0.01) | 0.06(0.05-0.09) | -0.05(-0.39-0.3) |
| Palestine | 0.28(0.20-0.38) | 0.06(0.05-0.09) | 0.75(0.59-0.94) | 0.04(0.03-0.06) | -1.26*(-1.42--1.1) |
| Panama | 0.40(0.36-0.44) | 0.05(0.05-0.06) | 0.93(0.71-1.16) | 0.05(0.04-0.06) | 0.04(-0.6-0.69) |
| Papua New Guinea | 0.75(0.42-1.12) | 0.06(0.04-0.09) | 2.00(1.34-3.14) | 0.05(0.04-0.08) | -0.53*(-0.61--0.44) |
| Paraguay | 0.37(0.30-0.47) | 0.03(0.03-0.04) | 1.20(0.85-1.62) | 0.04(0.03-0.06) | 0.86*(0.41-1.32) |
| Peru | 4.50(3.55-5.64) | 0.07(0.05-0.09) | 8.93(6.43-12.10) | 0.06(0.04-0.08) | -0.67(-1.87-0.55) |
| Philippines | 15.40(11.60-18.05) | 0.08(0.06-0.10) | 44.15(34.86-53.58) | 0.10(0.08-0.12) | 0.57*(0.39-0.74) |
| Poland | 6.96(6.71-7.24) | 0.05(0.05-0.05) | 7.66(6.95-8.41) | 0.04(0.03-0.04) | -0.6(-1.48-0.29) |
| Portugal | 1.52(1.39-1.67) | 0.04(0.03-0.04) | 1.64(1.51-1.79) | 0.03(0.03-0.03) | -0.76*(-1.13--0.38) |
| Puerto Rico | 0.86(0.76-0.96) | 0.06(0.05-0.06) | 0.91(0.72-1.13) | 0.06(0.05-0.07) | 0.55*(0.1-0.99) |
| Qatar | 0.15(0.11-0.19) | 0.08(0.06-0.10) | 0.85(0.59-1.22) | 0.05(0.03-0.07) | -1.79*(-2.89--0.68) |
| Republic of Korea | 12.19(10.05-14.76) | 0.07(0.06-0.09) | 12.79(10.50-15.43) | 0.04(0.04-0.05) | -1.63*(-1.85--1.42) |
| Republic of Moldova | 0.56(0.51-0.61) | 0.03(0.03-0.04) | 0.53(0.46-0.60) | 0.03(0.02-0.03) | -0.73(-1.66-0.21) |
| Romania | 5.23(4.73-5.78) | 0.06(0.05-0.06) | 5.07(4.30-5.87) | 0.05(0.04-0.06) | -0.41(-1.37-0.55) |
| Russian Federation | 20.28(19.81-20.83) | 0.04(0.04-0.04) | 29.32(26.98-31.74) | 0.04(0.04-0.04) | 0.18(-0.74-1.1) |
| Rwanda | 1.44(0.84-2.39) | 0.10(0.06-0.16) | 2.37(1.44-3.59) | 0.06(0.04-0.09) | -1.60*(-1.83--1.37) |
| Saint Kitts and Nevis | 0.01(0.01-0.01) | 0.12(0.11-0.14) | 0.02(0.02-0.03) | 0.08(0.06-0.10) | -1.49*(-2.04--0.93) |
| Saint Lucia | 0.03(0.03-0.04) | 0.09(0.08-0.10) | 0.07(0.06-0.09) | 0.08(0.06-0.10) | -0.39*(-0.74--0.03) |
| Saint Vincent and the Grenadines | 0.02(0.02-0.02) | 0.07(0.06-0.07) | 0.04(0.04-0.05) | 0.08(0.07-0.10) | 0.55*(0.03-1.08) |
| Samoa | 0.02(0.02-0.04) | 0.06(0.04-0.09) | 0.04(0.03-0.06) | 0.06(0.04-0.08) | -0.15*(-0.21--0.08) |
| San Marino | 0.00(0.00-0.00) | 0.02(0.01-0.02) | 0.00(0.00-0.00) | 0.01(0.01-0.02) | -1.79*(-2.11--1.47) |
| Sao Tome and Principe | 0.02(0.01-0.02) | 0.07(0.05-0.09) | 0.06(0.04-0.10) | 0.08(0.05-0.12) | 0.48*(0.06-0.92) |
| Saudi Arabia | 7.11(4.78-10.10) | 0.15(0.10-0.21) | 39.43(27.91-54.19) | 0.17(0.12-0.23) | 0.40*(0.25-0.55) |
| Senegal | 1.32(0.93-1.91) | 0.07(0.05-0.10) | 4.09(2.56-6.93) | 0.09(0.06-0.15) | 0.62*(0.19-1.06) |
| Serbia | 1.87(1.49-2.34) | 0.04(0.04-0.06) | 1.43(1.12-1.80) | 0.03(0.03-0.04) | -0.90*(-1.22--0.57) |
| Seychelles | 0.04(0.03-0.04) | 0.18(0.14-0.21) | 0.07(0.06-0.09) | 0.13(0.11-0.16) | -0.91*(-1.29--0.53) |
| Sierra Leone | 0.73(0.49-1.03) | 0.07(0.04-0.09) | 1.99(1.20-3.26) | 0.08(0.05-0.12) | 0.49*(0.25-0.73) |
| Singapore | 0.61(0.55-0.68) | 0.04(0.04-0.05) | 0.89(0.81-0.98) | 0.03(0.02-0.03) | -1.65*(-2.34--0.96) |
| Slovakia | 1.21(0.97-1.49) | 0.06(0.04-0.07) | 0.96(0.76-1.22) | 0.03(0.03-0.04) | -1.79*(-2.09--1.48) |
| Slovenia | 0.42(0.38-0.47) | 0.05(0.04-0.05) | 0.24(0.19-0.29) | 0.02(0.02-0.03) | -2.46*(-2.89--2.02) |
| Solomon Islands | 0.07(0.03-0.11) | 0.09(0.04-0.13) | 0.21(0.14-0.29) | 0.08(0.06-0.12) | -0.16*(-0.31--0.01) |
| Somalia | 1.39(0.86-2.19) | 0.07(0.04-0.11) | 3.36(1.97-5.47) | 0.06(0.03-0.10) | -0.41*(-0.48--0.33) |
| South Africa | 7.73(6.54-9.11) | 0.07(0.06-0.08) | 17.78(14.37-21.93) | 0.07(0.06-0.09) | 0.14(-0.32-0.61) |
| South Sudan | 0.83(0.56-1.16) | 0.06(0.04-0.08) | 1.79(1.18-2.66) | 0.06(0.04-0.09) | -0.01(-0.23-0.21) |
| Spain | 6.12(5.55-6.84) | 0.04(0.03-0.04) | 7.48(6.75-8.28) | 0.03(0.03-0.03) | -0.77*(-0.95--0.6) |
| Sri Lanka | 4.65(3.68-5.91) | 0.07(0.06-0.09) | 5.71(3.35-8.23) | 0.06(0.03-0.08) | -0.85*(-1.27--0.42) |
| Sudan | 4.53(2.90-6.83) | 0.09(0.06-0.13) | 9.83(6.07-14.77) | 0.07(0.05-0.11) | -0.56*(-0.61--0.51) |
| Suriname | 0.07(0.05-0.08) | 0.05(0.04-0.06) | 0.17(0.12-0.22) | 0.06(0.05-0.08) | 0.68(-0.77-2.15) |
| Sweden | 1.58(1.49-1.69) | 0.04(0.03-0.04) | 1.04(0.90-1.18) | 0.02(0.02-0.03) | -1.59*(-1.96--1.22) |
| Switzerland | 1.17(1.06-1.29) | 0.03(0.03-0.04) | 0.87(0.79-0.95) | 0.02(0.02-0.02) | -1.78*(-2.19--1.37) |
| Syrian Arab Republic | 2.61(1.79-3.60) | 0.09(0.06-0.12) | 4.86(3.38-6.80) | 0.07(0.05-0.10) | -0.85*(-1.4--0.31) |
| Taiwan (Province of China) | 7.62(6.90-8.45) | 0.11(0.10-0.12) | 11.90(10.64-13.25) | 0.09(0.08-0.10) | -0.48(-1.01-0.06) |
| Tajikistan | 0.85(0.59-1.16) | 0.07(0.05-0.09) | 2.13(1.59-2.80) | 0.06(0.04-0.08) | -0.61*(-0.78--0.44) |
| Thailand | 19.57(15.75-24.31) | 0.10(0.08-0.12) | 39.28(28.94-52.68) | 0.10(0.08-0.14) | 0.36*(0.06-0.66) |
| Timor-Leste | 0.15(0.11-0.21) | 0.07(0.05-0.09) | 0.27(0.19-0.39) | 0.06(0.04-0.09) | -0.1(-0.51-0.3) |
| Togo | 0.51(0.35-0.79) | 0.06(0.04-0.09) | 2.51(1.48-4.14) | 0.09(0.05-0.15) | 1.35*(1.15-1.54) |
| Tokelau | 0.00(0.00-0.00) | 0.07(0.05-0.10) | 0.00(0.00-0.00) | 0.05(0.04-0.08) | -0.68*(-1--0.37) |
| Tonga | 0.01(0.01-0.02) | 0.05(0.03-0.07) | 0.02(0.01-0.03) | 0.05(0.03-0.07) | 0.03(-0.12-0.18) |
| Trinidad and Tobago | 0.24(0.21-0.26) | 0.06(0.05-0.06) | 0.45(0.33-0.60) | 0.07(0.05-0.09) | 0.89*(0.05-1.75) |
| Tunisia | 1.22(0.92-1.73) | 0.05(0.04-0.07) | 3.19(2.10-4.66) | 0.05(0.04-0.08) | 0.07(-0.03-0.16) |
| Türkiye | 16.49(11.98-21.31) | 0.09(0.07-0.12) | 24.40(18.61-31.44) | 0.06(0.04-0.07) | -1.50*(-1.72--1.29) |
| Turkmenistan | 0.41(0.38-0.44) | 0.04(0.04-0.05) | 1.33(0.97-1.76) | 0.06(0.05-0.08) | 1.32*(0.58-2.06) |
| Tuvalu | 0.00(0.00-0.00) | 0.09(0.06-0.12) | 0.00(0.00-0.00) | 0.08(0.05-0.11) | -0.38*(-0.44--0.32) |
| Uganda | 1.90(1.22-3.10) | 0.05(0.03-0.08) | 5.78(3.58-8.52) | 0.06(0.03-0.08) | 0.22*(0.05-0.38) |
| Ukraine | 9.69(8.39-11.50) | 0.05(0.04-0.05) | 12.00(8.34-16.09) | 0.05(0.04-0.07) | 0.65(-0.39-1.7) |
| United Arab Emirates | 0.53(0.33-0.77) | 0.07(0.05-0.11) | 2.24(1.68-2.90) | 0.02(0.02-0.03) | -3.46*(-4.04--2.87) |
| United Kingdom | 9.20(9.04-9.37) | 0.04(0.03-0.04) | 11.92(11.63-12.19) | 0.04(0.04-0.04) | 0.14(-0.64-0.92) |
| United Republic of Tanzania | 3.19(2.33-4.31) | 0.05(0.04-0.07) | 8.64(5.58-12.94) | 0.05(0.03-0.08) | -0.24*(-0.38--0.09) |
| United States of America | 57.36(56.02-58.77) | 0.05(0.05-0.05) | 88.70(85.75-91.79) | 0.06(0.06-0.06) | 0.46*(0.23-0.7) |
| United States Virgin Islands | 0.05(0.04-0.07) | 0.10(0.07-0.13) | 0.03(0.02-0.04) | 0.09(0.06-0.12) | -0.21(-0.68-0.26) |
| Uruguay | 0.52(0.48-0.58) | 0.04(0.04-0.05) | 0.73(0.65-0.81) | 0.05(0.04-0.05) | 0.39(-0.07-0.86) |
| Uzbekistan | 1.96(1.59-2.40) | 0.04(0.03-0.05) | 4.62(3.77-5.53) | 0.03(0.03-0.04) | -0.68(-1.49-0.13) |
| Vanuatu | 0.04(0.02-0.06) | 0.09(0.05-0.14) | 0.09(0.06-0.13) | 0.08(0.06-0.12) | -0.28*(-0.42--0.13) |
| Venezuela (Bolivarian Republic of) | 2.95(2.70-3.25) | 0.05(0.05-0.06) | 8.31(6.09-11.10) | 0.06(0.05-0.09) | 0.79*(0.35-1.24) |
| Viet Nam | 12.94(9.04-17.91) | 0.08(0.06-0.11) | 40.92(28.64-60.63) | 0.08(0.06-0.13) | 0.13*(0.07-0.18) |
| Yemen | 2.39(1.51-3.68) | 0.08(0.05-0.13) | 7.28(4.46-11.18) | 0.07(0.05-0.11) | -0.30*(-0.57--0.04) |
| Zambia | 1.07(0.79-1.38) | 0.06(0.04-0.08) | 3.40(2.27-4.87) | 0.06(0.04-0.09) | 0.11(-0.06-0.28) |
| Zimbabwe | 1.41(1.06-1.86) | 0.06(0.04-0.08) | 5.48(3.43-8.06) | 0.11(0.07-0.16) | 2.00*(1.41-2.6) |

Note: UI: uncertainty interval; CI, confidence interval; AAPC, average annual percent of change; ASMR, age-standardized death rate

Table S4. The case number and age-standardized rate of DALYs among 204 countries and territories, with the AAPCs between 1990 and 2021

|  | 1990 | | 2021 | |  |
| --- | --- | --- | --- | --- | --- |
|  | Number (95%UI) | ASDR per 100,000 populations (95%UI) | Number (95%UI) | ASDR per 100,000 populations (95%UI) | AAPCs (95%CI) |
| Afghanistan | 302.79(139.65-485.93) | 9.85(4.54-15.87) | 896.16(475.82-1452.78) | 9.25(4.93-14.91) | -0.18*(-0.25--0.11) |
| Albania | 33.17(24.59-44.86) | 2.95(2.20-3.97) | 29.49(21.33-40.81) | 2.64(1.91-3.66) | -0.43(-0.99-0.14) |
| Algeria | 282.29(202.55-382.79) | 4.65(3.39-6.18) | 923.67(665.05-1273.83) | 4.68(3.38-6.45) | 0.02(-0.06-0.11) |
| American Samoa | 0.66(0.49-0.87) | 4.31(3.22-5.67) | 0.87(0.64-1.17) | 3.97(2.90-5.34) | -0.26*(-0.37--0.16) |
| Andorra | 0.93(0.62-1.36) | 3.49(2.37-5.09) | 1.70(1.07-2.52) | 3.24(2.00-4.88) | -0.32*(-0.58--0.05) |
| Angola | 121.47(83.99-165.11) | 4.42(3.08-5.96) | 384.82(272.61-517.64) | 4.28(3.05-5.71) | -0.1(-0.5-0.3) |
| Antigua and Barbuda | 1.03(0.75-1.44) | 4.96(3.66-6.86) | 2.08(1.45-2.97) | 4.58(3.18-6.58) | 0.21*(0.05-0.37) |
| Argentine | 368.51(296.76-460.70) | 2.85(2.30-3.56) | 579.37(440.00-771.22) | 2.77(2.10-3.70) | -0.08(-0.29-0.13) |
| Armenia | 31.98(24.96-41.66) | 2.98(2.36-3.82) | 32.33(23.38-44.13) | 2.41(1.75-3.27) | -0.75*(-1.11--0.4) |
| Australia | 162.31(129.44-208.45) | 2.14(1.71-2.74) | 306.37(245.14-390.70) | 2.58(2.05-3.31) | 0.62*(0.37-0.87) |
| Austria | 134.46(100.84-185.16) | 3.71(2.76-5.13) | 135.66(90.76-199.55) | 3.23(2.14-4.78) | -0.49*(-0.71--0.27) |
| Azerbaijan | 73.44(52.78-97.81) | 3.54(2.57-4.67) | 124.84(89.29-172.51) | 2.58(1.86-3.55) | -1.02*(-1.23--0.8) |
| Bahamas | 5.92(4.59-7.78) | 6.49(5.07-8.45) | 13.48(10.00-17.72) | 7.04(5.21-9.29) | 0.28(-0.43-1) |
| Bahrain | 7.35(5.46-9.85) | 4.40(3.37-5.72) | 35.14(24.78-49.96) | 3.62(2.54-5.15) | -0.61*(-1.05--0.16) |
| Bangladesh | 1247.18(893.60-1711.87) | 4.38(3.18-5.96) | 2616.91(1697.06-3805.28) | 3.83(2.49-5.56) | -0.43*(-0.52--0.34) |
| Barbados | 4.52(3.31-6.26) | 4.75(3.52-6.54) | 6.69(4.73-9.40) | 4.70(3.29-6.65) | -0.01(-0.34-0.32) |
| Belarus | 116.57(85.42-161.66) | 2.98(2.21-4.09) | 136.41(100.32-187.52) | 2.94(2.15-4.06) | 0.06(-0.4-0.51) |
| Belgium | 149.21(108.17-206.09) | 3.49(2.54-4.81) | 185.31(127.43-270.11) | 3.61(2.45-5.30) | 0.06(-0.02-0.14) |
| Belize | 2.03(1.44-2.85) | 4.41(3.17-6.15) | 10.57(7.97-14.13) | 6.08(4.59-8.11) | 0.94*(0.33-1.57) |
| Benin | 53.93(38.33-73.18) | 4.87(3.52-6.53) | 194.45(124.66-294.76) | 5.25(3.40-7.90) | 0.20*(0.07-0.32) |
| Bermuda | 1.56(1.16-2.11) | 5.40(4.03-7.29) | 1.34(0.86-2.01) | 4.24(2.69-6.42) | -0.80*(-0.87--0.72) |
| Bhutan | 7.39(4.86-10.49) | 4.63(3.08-6.49) | 14.77(10.10-20.82) | 4.53(3.12-6.35) | -0.08(-0.17-0.01) |
| Bolivia | 162.69(112.63-228.18) | 8.57(5.98-11.95) | 359.16(235.05-533.30) | 7.54(4.95-11.16) | -0.41*(-0.53--0.29) |
| Bosnia and Herzegovina | 59.71(46.11-77.61) | 3.32(2.58-4.29) | 43.29(31.25-57.46) | 2.67(1.91-3.57) | -0.79*(-1.18--0.41) |
| Botswana | 15.38(10.61-21.57) | 4.59(3.19-6.39) | 41.75(29.41-58.86) | 3.94(2.79-5.52) | -0.49(-1.05-0.08) |
| Brazil | 2238.60(1738.46-2872.01) | 4.49(3.53-5.70) | 5394.22(4174.82-6990.59) | 5.05(3.90-6.55) | 0.40*(0.34-0.46) |
| Brunei Darussalam | 3.98(2.66-5.52) | 4.72(3.22-6.46) | 12.08(7.86-16.22) | 5.12(3.33-6.87) | 0.29*(0-0.57) |
| Bulgaria | 132.24(111.56-160.08) | 3.35(2.82-4.07) | 135.47(111.02-166.56) | 3.85(3.12-4.77) | 0.5(-0.1-1.1) |
| Burkina Faso | 107.27(72.30-149.09) | 4.61(3.13-6.37) | 318.04(199.19-476.52) | 5.19(3.28-7.73) | 0.40*(0.24-0.55) |
| Burundi | 63.39(42.54-92.92) | 4.84(3.28-7.04) | 146.73(92.73-209.38) | 4.10(2.62-5.80) | -0.56*(-0.69--0.43) |
| Cabo Verde | 3.07(2.19-4.20) | 4.94(3.63-6.60) | 14.84(10.46-21.43) | 6.39(4.54-9.18) | 0.83*(0.55-1.12) |
| Cambodia | 164.42(120.63-219.42) | 6.12(4.55-8.10) | 368.46(265.83-515.68) | 5.74(4.16-8.00) | -0.19*(-0.23--0.15) |
| Cameroon | 153.24(111.06-207.78) | 5.81(4.25-7.82) | 640.42(394.53-985.72) | 6.84(4.26-10.47) | 0.52*(0.44-0.61) |
| Canada | 438.15(335.64-571.75) | 3.50(2.70-4.55) | 782.87(589.42-1029.35) | 4.66(3.49-6.16) | 0.93*(0.85-1.01) |
| Central African Republic | 40.19(25.33-56.25) | 5.52(3.49-7.68) | 90.85(57.79-133.31) | 5.28(3.37-7.73) | -0.16(-0.36-0.03) |
| Chad | 57.49(40.67-80.02) | 4.00(2.87-5.51) | 224.92(150.35-327.66) | 5.47(3.69-7.92) | 1.05*(0.88-1.22) |
| Chile | 162.26(133.84-198.16) | 3.23(2.68-3.91) | 246.81(190.14-328.41) | 2.84(2.18-3.78) | -0.41*(-0.74--0.07) |
| China | 29722.46(23780.16-35782.28) | 6.98(5.61-8.35) | 51112.70(39213.27-67425.00) | 6.92(5.31-9.14) | -0.04(-0.14-0.06) |
| Colombia | 547.51(409.06-739.20) | 5.12(3.86-6.87) | 1030.22(727.80-1448.01) | 4.82(3.41-6.78) | -0.25*(-0.37--0.13) |
| Comoros | 5.27(3.47-7.27) | 4.48(2.98-6.15) | 11.93(8.19-16.37) | 4.08(2.81-5.58) | -0.25(-0.61-0.12) |
| Congo | 35.00(22.90-48.48) | 5.95(3.91-8.19) | 100.50(69.57-139.74) | 4.75(3.29-6.59) | -0.72*(-1.02--0.42) |
| Cook Islands | 0.28(0.20-0.39) | 4.23(3.06-5.84) | 0.26(0.18-0.37) | 3.48(2.37-5.00) | -0.63*(-0.74--0.52) |
| Costa Rica | 42.38(29.55-61.89) | 4.46(3.15-6.45) | 115.20(80.55-165.53) | 5.43(3.80-7.79) | 0.55*(0.36-0.73) |
| Côte d'Ivoire | 185.86(135.58-247.29) | 6.03(4.45-7.94) | 593.91(384.61-898.69) | 6.49(4.23-9.76) | 0.25*(0.05-0.46) |
| Croatia | 65.84(52.87-83.13) | 3.01(2.42-3.79) | 46.91(35.98-62.09) | 2.35(1.79-3.14) | -0.86*(-1.14--0.58) |
| Cuba | 182.45(134.38-248.91) | 4.04(2.97-5.51) | 244.39(174.93-336.20) | 4.46(3.14-6.22) | 0.35*(0.1-0.59) |
| Cyprus | 12.05(8.42-16.95) | 3.57(2.49-5.01) | 20.89(13.30-31.62) | 2.95(1.88-4.46) | -0.60*(-0.81--0.39) |
| Czech | 152.85(125.21-189.67) | 3.06(2.50-3.81) | 135.66(104.45-179.06) | 2.29(1.74-3.07) | -0.96*(-1.19--0.72) |
| Democratic People's Republic of Korea | 689.06(483.67-964.14) | 7.98(5.60-11.15) | 1210.26(818.48-1711.97) | 8.98(6.06-12.72) | 0.39*(0.3-0.49) |
| Democratic Republic of the Congo | 371.27(257.38-513.25) | 4.03(2.83-5.52) | 1067.81(722.88-1485.82) | 4.08(2.78-5.62) | 0.08*(0.01-0.16) |
| Denmark | 81.88(58.94-112.82) | 3.19(2.28-4.43) | 81.20(50.99-123.19) | 3.14(1.94-4.81) | -0.01(-0.11-0.08) |
| Djibouti | 4.21(2.84-5.98) | 3.59(2.44-5.07) | 19.83(13.07-29.51) | 3.76(2.49-5.58) | 0.12*(0.04-0.21) |
| Dominica | 1.21(0.86-1.67) | 5.37(3.84-7.42) | 1.69(1.20-2.32) | 5.93(4.23-8.18) | 0.31*(0.2-0.42) |
| Dominican Republic | 105.90(78.23-141.17) | 4.95(3.69-6.55) | 271.48(195.12-366.43) | 5.79(4.17-7.81) | 0.47(-0.04-0.99) |
| Ecuador | 177.91(121.16-257.96) | 5.86(4.02-8.45) | 516.65(332.61-778.85) | 6.97(4.49-10.50) | 0.52*(0.11-0.94) |
| Egypt | 980.20(786.21-1216.29) | 5.58(4.50-6.89) | 1870.51(1381.78-2572.05) | 4.73(3.51-6.48) | -0.52*(-0.81--0.23) |
| El Salvador | 97.51(74.55-129.01) | 6.42(4.95-8.46) | 178.06(128.59-239.23) | 7.01(5.06-9.41) | 0.15(-0.44-0.75) |
| Equatorial Guinea | 5.54(3.66-7.88) | 4.95(3.28-7.01) | 18.80(12.61-27.18) | 4.40(2.99-6.29) | -0.36*(-0.52--0.21) |
| Eritrea | 49.49(33.35-67.70) | 5.42(3.67-7.37) | 118.69(81.82-167.46) | 5.31(3.68-7.46) | -0.08(-0.2-0.03) |
| Estonia | 21.74(15.19-31.03) | 3.22(2.25-4.60) | 15.39(10.63-22.48) | 2.45(1.67-3.61) | -0.97*(-1.25--0.69) |
| Eswatini | 10.79(7.32-14.72) | 5.36(3.64-7.30) | 30.34(18.46-43.63) | 7.71(4.68-11.11) | 1.22*(0.99-1.44) |
| Ethiopia | 663.83(482.77-892.70) | 5.23(3.83-7.00) | 1093.86(753.87-1597.67) | 3.45(2.40-5.02) | -1.35*(-1.46--1.24) |
| Fiji | 11.70(8.84-15.45) | 4.43(3.37-5.82) | 15.36(11.33-21.00) | 3.93(2.90-5.38) | -0.35*(-0.55--0.15) |
| Finland | 102.07(76.71-140.33) | 4.02(3.01-5.55) | 84.54(56.73-125.86) | 3.65(2.44-5.46) | -0.26*(-0.5--0.01) |
| French | 927.70(697.52-1273.07) | 3.80(2.87-5.20) | 1062.02(731.00-1525.98) | 3.65(2.48-5.30) | -0.12(-0.28-0.04) |
| Gabonese | 14.35(9.61-20.01) | 5.90(3.98-8.16) | 32.55(22.55-45.65) | 5.11(3.56-7.14) | -0.40*(-0.61--0.19) |
| Gambia | 11.11(7.00-17.08) | 4.49(2.88-6.83) | 38.19(23.31-61.33) | 5.54(3.42-8.82) | 0.67(-0.08-1.43) |
| Georgia | 73.00(56.84-94.89) | 3.56(2.79-4.61) | 54.24(41.79-71.13) | 3.35(2.57-4.41) | -0.23(-0.75-0.29) |
| Germany | 1387.83(1009.55-1904.38) | 3.81(2.76-5.24) | 1336.91(910.55-1970.77) | 3.74(2.54-5.54) | -0.06(-0.16-0.03) |
| Ghana | 173.44(119.85-243.85) | 4.29(3.01-5.96) | 557.21(376.10-817.64) | 4.73(3.22-6.90) | 0.32*(0.25-0.39) |
| Greece | 135.56(94.58-193.06) | 3.02(2.10-4.31) | 185.54(127.67-265.25) | 3.61(2.44-5.23) | 0.57*(0.51-0.62) |
| Greenland | 1.72(1.06-2.33) | 6.80(4.21-9.18) | 1.13(0.75-1.50) | 5.21(3.46-6.91) | -0.79*(-0.83--0.74) |
| Grenada | 1.15(0.85-1.57) | 5.03(3.76-6.81) | 2.53(1.87-3.40) | 5.46(4.00-7.37) | 0.25*(0.03-0.48) |
| Guam | 1.79(1.21-2.54) | 3.51(2.39-4.94) | 2.54(1.76-3.52) | 3.74(2.58-5.21) | 0.16(-0.34-0.67) |
| Guatemala | 128.76(100.58-167.75) | 5.95(4.68-7.71) | 319.53(236.72-441.68) | 5.58(4.15-7.68) | -0.25(-0.73-0.24) |
| Guinea | 70.83(49.23-100.09) | 4.22(2.95-5.92) | 183.37(118.83-285.42) | 5.15(3.38-7.94) | 0.62*(0.52-0.73) |
| Guinea-Bissau | 19.38(11.86-28.20) | 8.03(4.95-11.60) | 48.26(31.32-69.40) | 8.28(5.43-11.82) | 0.14*(0.06-0.22) |
| Guyana | 13.10(9.94-17.54) | 5.56(4.26-7.38) | 22.03(16.16-29.53) | 6.98(5.12-9.37) | 0.75*(0.51-1) |
| Haiti | 133.72(83.52-183.35) | 7.16(4.49-9.77) | 323.39(213.05-451.65) | 6.58(4.34-9.15) | -0.25*(-0.38--0.12) |
| Honduras | 103.65(81.26-131.02) | 8.60(6.79-10.81) | 290.35(191.20-414.20) | 7.96(5.27-11.30) | -0.25*(-0.35--0.14) |
| Hungary | 181.09(150.93-217.44) | 3.72(3.09-4.48) | 142.39(112.42-180.96) | 2.67(2.08-3.45) | -1.03*(-1.55--0.51) |
| Iceland | 4.06(2.99-5.69) | 4.00(2.98-5.56) | 6.63(4.66-9.26) | 4.22(2.96-5.93) | 0.21(-0.08-0.51) |
| India | 13006.24(9949.75-16847.33) | 4.54(3.50-5.86) | 30974.66(23451.88-39979.89) | 5.11(3.88-6.58) | 0.37*(0.26-0.49) |
| Indonesia | 3268.65(2594.28-4064.99) | 5.43(4.34-6.72) | 7338.57(5639.02-9460.65) | 5.31(4.07-6.86) | -0.06*(-0.1--0.02) |
| Iran | 595.15(405.92-761.20) | 4.40(3.03-5.53) | 1838.05(1226.60-2390.90) | 4.15(2.76-5.38) | -0.24*(-0.36--0.13) |
| Iraq | 326.34(245.04-429.81) | 7.14(5.39-9.37) | 1055.82(775.58-1440.31) | 6.29(4.62-8.58) | -0.43*(-0.51--0.36) |
| Ireland | 47.93(34.38-67.76) | 3.38(2.43-4.77) | 82.70(52.96-123.35) | 3.34(2.11-5.03) | -0.04(-0.33-0.26) |
| Israel | 62.66(41.53-93.86) | 3.52(2.36-5.23) | 143.19(89.63-220.56) | 3.53(2.19-5.46) | 0(-0.2-0.2) |
| Italy | 1033.85(794.72-1362.60) | 4.07(3.11-5.38) | 1098.40(817.19-1479.80) | 3.75(2.71-5.17) | -0.32*(-0.64--0.01) |
| Jamaica | 25.57(17.05-37.63) | 3.75(2.54-5.47) | 59.69(41.23-84.79) | 4.87(3.37-6.92) | 1.09*(0.07-2.11) |
| Japan | 2017.40(1635.37-2536.14) | 3.01(2.41-3.82) | 2016.47(1653.27-2505.03) | 3.15(2.52-4.00) | 0.15(-0.14-0.44) |
| Jordan | 48.14(36.05-62.23) | 5.36(4.05-6.87) | 187.30(136.58-256.05) | 3.61(2.64-4.93) | -1.23*(-1.51--0.95) |
| Kazakhstan | 221.39(173.89-286.42) | 4.03(3.20-5.16) | 331.59(254.74-432.64) | 3.91(3.00-5.10) | -0.15(-0.73-0.43) |
| Kenya | 166.22(122.84-221.61) | 3.17(2.38-4.17) | 638.43(479.85-834.83) | 3.92(2.98-5.08) | 0.69*(0.6-0.78) |
| Kiribati | 1.13(0.84-1.51) | 4.96(3.72-6.58) | 2.11(1.52-2.82) | 4.74(3.43-6.31) | -0.13*(-0.21--0.05) |
| Kuwait | 38.80(31.82-48.77) | 6.16(5.14-7.62) | 100.85(70.95-142.10) | 3.18(2.24-4.48) | -2.10*(-3.17--1.01) |
| Kyrgyz | 43.31(32.62-56.65) | 3.68(2.81-4.79) | 80.83(61.36-106.12) | 3.05(2.32-3.99) | -0.62*(-0.94--0.31) |
| Lao People's Democratic Republic | 81.11(51.58-114.96) | 7.11(4.55-10.04) | 162.73(117.32-217.94) | 5.68(4.12-7.58) | -0.73*(-0.79--0.66) |
| Latvia | 37.80(27.77-51.42) | 3.16(2.32-4.32) | 24.74(18.43-33.40) | 2.78(2.05-3.79) | -0.35(-1.19-0.5) |
| Lebanese | 57.88(32.70-84.55) | 5.64(3.18-8.23) | 91.98(65.89-125.52) | 3.69(2.66-5.01) | -1.32*(-1.48--1.17) |
| Lesotho | 15.30(10.84-21.30) | 3.45(2.46-4.78) | 35.72(23.47-49.70) | 5.71(3.74-7.93) | 1.67*(1.45-1.89) |
| Liberia | 28.62(19.31-41.82) | 4.79(3.29-6.91) | 112.81(67.40-191.08) | 5.59(3.35-9.43) | 0.46*(0.28-0.64) |
| Libya | 48.62(35.22-67.68) | 4.51(3.30-6.20) | 271.46(183.15-422.07) | 6.93(4.67-10.80) | 1.39*(1.22-1.56) |
| Lithuania | 47.64(34.68-66.46) | 3.03(2.20-4.22) | 34.83(25.56-48.49) | 2.77(2.00-3.90) | -0.29(-0.99-0.41) |
| Luxembourg | 7.24(5.43-9.87) | 4.03(3.02-5.49) | 10.76(7.00-16.53) | 3.23(2.09-5.00) | -0.76*(-0.97--0.55) |
| Madagascar | 104.59(75.81-138.66) | 3.61(2.66-4.72) | 306.77(210.97-435.99) | 3.50(2.42-4.95) | -0.08(-0.25-0.09) |
| Malawi | 94.14(68.50-129.18) | 3.88(2.87-5.26) | 231.56(156.19-319.94) | 4.27(2.91-5.86) | 0.34*(0.24-0.44) |
| Malaysia | 233.51(177.36-305.27) | 4.07(3.14-5.25) | 681.32(522.37-881.46) | 4.95(3.82-6.37) | 0.66*(0.41-0.91) |
| Maldives | 2.83(1.55-4.07) | 5.38(2.97-7.71) | 9.84(6.66-14.53) | 3.43(2.36-5.02) | -1.51*(-1.6--1.41) |
| Mali | 116.09(83.07-158.80) | 5.06(3.65-6.89) | 296.03(195.12-443.76) | 5.07(3.38-7.54) | 0.04(-0.08-0.16) |
| Malta | 5.74(4.10-8.13) | 3.24(2.31-4.58) | 8.08(5.68-11.61) | 3.87(2.70-5.60) | 0.59*(0.36-0.82) |
| Marshall Islands | 0.61(0.46-0.80) | 5.62(4.32-7.21) | 1.42(0.98-1.92) | 5.93(4.11-8.02) | 0.20*(0.11-0.3) |
| Mauritania | 22.42(15.06-32.14) | 4.25(2.89-6.04) | 48.13(29.15-81.61) | 3.78(2.31-6.37) | -0.38*(-0.68--0.08) |
| Mauritius | 20.82(16.81-26.24) | 5.28(4.33-6.57) | 34.45(27.63-43.02) | 5.48(4.36-6.88) | 0.19(-0.18-0.56) |
| Mexico | 1527.80(1256.79-1884.93) | 6.01(4.98-7.37) | 3827.08(3041.52-4851.59) | 6.42(5.10-8.15) | 0.27*(0.16-0.38) |
| Micronesia | 1.61(1.13-2.17) | 5.83(4.13-7.82) | 2.14(1.51-2.89) | 5.31(3.75-7.15) | -0.32*(-0.37--0.27) |
| Monaco | 0.59(0.42-0.81) | 3.80(2.67-5.28) | 0.69(0.45-1.00) | 4.25(2.76-6.30) | 0.36*(0.33-0.4) |
| Mongolia | 20.30(15.42-26.34) | 3.54(2.72-4.55) | 51.09(39.27-65.02) | 3.39(2.61-4.31) | -0.19(-0.81-0.43) |
| Montenegro | 8.32(6.37-10.91) | 3.41(2.63-4.45) | 8.79(6.73-11.63) | 2.97(2.26-3.96) | -0.44*(-0.79--0.1) |
| Morocco | 357.56(259.92-485.82) | 4.84(3.56-6.49) | 986.52(682.49-1365.07) | 5.91(4.09-8.19) | 0.66*(0.59-0.72) |
| Mozambique | 141.21(98.96-195.07) | 3.78(2.66-5.19) | 399.38(267.11-565.52) | 4.91(3.30-6.92) | 0.85*(0.77-0.93) |
| Myanmar | 748.57(494.19-1030.63) | 6.23(4.15-8.52) | 1378.62(985.05-1848.47) | 5.66(4.04-7.60) | -0.31*(-0.37--0.25) |
| Namibia | 16.93(12.25-22.79) | 4.56(3.33-6.08) | 47.76(32.68-65.98) | 5.36(3.69-7.37) | 0.57*(0.37-0.77) |
| Nauru | 0.26(0.16-0.38) | 7.77(4.70-11.16) | 0.27(0.16-0.39) | 6.88(4.08-9.77) | -0.39*(-0.45--0.32) |
| Nepal | 229.02(154.67-321.46) | 3.96(2.69-5.53) | 507.14(351.77-706.80) | 4.37(3.04-6.07) | 0.33*(0.24-0.42) |
| Netherlands | 242.26(173.94-352.09) | 3.44(2.47-4.99) | 258.15(172.73-382.91) | 3.48(2.30-5.23) | 0.03(-0.15-0.22) |
| New Zealand | 39.15(32.80-47.14) | 2.67(2.25-3.21) | 67.17(56.79-80.57) | 2.98(2.51-3.59) | 0.32(-0.22-0.85) |
| Nicaragua | 47.07(33.56-65.55) | 4.95(3.57-6.84) | 134.12(94.62-188.85) | 5.02(3.56-7.03) | -0.07(-0.29-0.16) |
| Niger | 93.92(65.65-130.24) | 4.56(3.22-6.29) | 245.21(154.81-381.87) | 4.54(2.90-7.01) | -0.02(-0.22-0.17) |
| Nigeria | 1317.15(937.27-1779.80) | 5.31(3.81-7.14) | 3271.55(2097.98-5346.41) | 5.03(3.25-8.20) | -0.18*(-0.27--0.09) |
| Niue | 0.04(0.03-0.06) | 5.10(3.68-7.11) | 0.03(0.02-0.05) | 4.42(3.10-6.22) | -0.49*(-0.66--0.31) |
| North Macedonia | 25.68(19.60-33.69) | 3.07(2.35-4.02) | 28.29(21.15-38.35) | 2.45(1.82-3.34) | -0.80*(-1.01--0.6) |
| Northern Mariana Islands | 0.94(0.63-1.36) | 4.49(3.03-6.51) | 0.93(0.68-1.27) | 3.71(2.67-5.08) | -0.53*(-0.76--0.3) |
| Norway | 51.37(41.05-64.47) | 2.67(2.13-3.35) | 82.76(61.62-111.30) | 3.27(2.40-4.44) | 0.68*(0.41-0.95) |
| Oman | 33.88(24.16-45.75) | 5.68(4.09-7.60) | 104.50(73.39-143.53) | 4.35(3.09-5.89) | -0.89*(-1.14--0.63) |
| Pakistan | 1362.20(1029.13-1774.02) | 4.35(3.31-5.65) | 4365.18(3178.26-5862.91) | 5.25(3.84-7.02) | 0.61*(0.57-0.66) |
| Palau | 0.28(0.20-0.39) | 4.76(3.43-6.55) | 0.51(0.36-0.69) | 4.90(3.45-6.76) | 0.12(-0.05-0.28) |
| Palestine | 18.75(13.60-25.62) | 4.21(3.09-5.70) | 62.85(44.56-87.40) | 3.68(2.63-5.09) | -0.51*(-0.58--0.43) |
| Panama | 38.79(27.75-54.10) | 4.82(3.47-6.69) | 97.17(64.74-143.53) | 5.36(3.57-7.92) | 0.35*(0.11-0.59) |
| Papua New Guinea | 50.45(33.02-72.59) | 4.14(2.73-5.93) | 147.56(100.84-212.79) | 3.87(2.65-5.56) | -0.22*(-0.28--0.17) |
| Paraguay | 43.01(27.99-63.69) | 3.61(2.38-5.28) | 130.96(86.91-192.41) | 4.60(3.08-6.72) | 0.81*(0.63-0.99) |
| Peru | 456.49(312.48-651.75) | 6.89(4.74-9.79) | 1175.87(768.20-1745.06) | 7.45(4.87-11.05) | 0.29(-0.21-0.79) |
| Philippines | 1020.64(800.08-1257.06) | 5.38(4.25-6.58) | 2813.09(2227.89-3462.14) | 6.22(4.93-7.64) | 0.52*(0.43-0.6) |
| Poland | 568.39(454.82-719.03) | 3.69(3.00-4.61) | 534.84(455.18-626.08) | 2.67(2.26-3.14) | -1.11*(-1.45--0.77) |
| Portuguese | 143.57(106.98-197.57) | 3.40(2.53-4.68) | 182.19(125.51-268.59) | 3.36(2.26-5.03) | -0.07(-0.21-0.08) |
| Puerto Rico | 74.35(53.67-100.97) | 4.95(3.58-6.72) | 79.35(57.33-108.17) | 5.39(3.87-7.39) | 0.3(-0.04-0.65) |
| Qatar | 10.07(7.49-13.45) | 5.08(3.84-6.65) | 74.46(49.25-108.24) | 3.89(2.59-5.60) | -0.93*(-1.69--0.18) |
| Republic of Korea | 748.99(594.06-952.51) | 4.27(3.42-5.38) | 942.93(718.08-1240.59) | 3.28(2.46-4.38) | -0.86*(-1--0.73) |
| Republic of Moldova | 49.51(36.33-69.41) | 2.94(2.19-4.08) | 48.63(34.56-68.91) | 2.52(1.78-3.59) | -0.52(-1.05-0.02) |
| Romania | 339.14(283.78-409.38) | 3.62(3.03-4.36) | 334.66(272.28-409.04) | 3.38(2.72-4.19) | -0.24(-0.92-0.44) |
| Russian | 1735.02(1344.48-2252.73) | 3.09(2.44-3.95) | 2328.85(1864.72-2964.53) | 3.18(2.54-4.06) | 0.12(-0.51-0.75) |
| Rwanda | 93.58(60.27-141.88) | 5.87(3.82-8.87) | 175.13(113.52-253.93) | 4.10(2.67-5.91) | -1.15*(-1.31--0.98) |
| Saint Kitts and Nevis | 0.84(0.68-1.05) | 8.00(6.57-9.91) | 1.90(1.41-2.62) | 6.12(4.50-8.50) | -0.90*(-1.23--0.57) |
| Saint Lucia | 2.41(1.85-3.11) | 6.27(4.86-8.03) | 5.62(4.17-7.53) | 6.15(4.54-8.28) | -0.11(-0.31-0.09) |
| Saint Vincent and the Grenadines | 1.50(1.11-2.02) | 5.22(3.93-6.96) | 3.24(2.50-4.23) | 6.16(4.72-8.08) | 0.47*(0.1-0.84) |
| Samoa | 1.74(1.21-2.56) | 4.05(2.84-5.91) | 2.93(2.05-4.18) | 4.09(2.86-5.81) | 0.02(-0.05-0.1) |
| San Marino | 0.29(0.18-0.44) | 2.73(1.73-4.20) | 0.40(0.22-0.64) | 2.53(1.37-4.12) | -0.29*(-0.37--0.21) |
| Sao Tome and Principe | 1.17(0.83-1.59) | 4.53(3.25-6.07) | 4.27(2.82-6.51) | 5.39(3.58-8.16) | 0.53*(0.2-0.86) |
| Saudi Arabia | 396.14(279.64-541.94) | 8.26(5.85-11.25) | 2270.53(1667.45-3049.17) | 9.75(7.18-13.06) | 0.55*(0.41-0.68) |
| Senegal | 85.04(57.64-123.94) | 4.57(3.13-6.60) | 260.27(166.27-417.91) | 5.53(3.56-8.84) | 0.60*(0.29-0.91) |
| Serbia | 127.63(98.85-166.68) | 3.04(2.35-3.98) | 106.92(81.37-140.40) | 2.44(1.84-3.22) | -0.64*(-0.84--0.44) |
| Seychelles | 2.13(1.70-2.58) | 9.84(7.93-11.87) | 4.37(3.49-5.50) | 7.97(6.33-10.06) | -0.65*(-0.96--0.34) |
| Sierra Leone | 47.59(31.77-67.22) | 4.23(2.87-5.90) | 129.15(80.97-203.86) | 4.87(3.08-7.64) | 0.48*(0.31-0.65) |
| Singapore | 45.46(35.37-58.75) | 3.21(2.53-4.10) | 86.38(61.55-122.69) | 2.53(1.80-3.61) | -0.78*(-1.11--0.46) |
| Slovak | 77.50(60.27-97.79) | 3.52(2.75-4.43) | 72.66(55.54-95.74) | 2.46(1.87-3.28) | -1.19*(-1.39--0.99) |
| Slovenia | 29.21(23.71-36.13) | 3.29(2.67-4.06) | 21.91(16.00-30.43) | 2.13(1.54-2.98) | -1.41*(-1.8--1.02) |
| Solomon Islands | 4.68(2.66-6.67) | 5.47(3.12-7.78) | 13.77(9.79-19.01) | 5.50(3.92-7.58) | -0.02(-0.18-0.13) |
| Somalia | 96.54(63.03-141.45) | 4.52(2.97-6.59) | 246.26(159.38-367.21) | 4.21(2.74-6.28) | -0.21*(-0.3--0.13) |
| South Africa | 551.59(442.13-678.72) | 4.65(3.75-5.69) | 1241.86(977.79-1556.71) | 4.96(3.92-6.20) | 0.13(-0.17-0.44) |
| South Sudan | 58.78(40.25-81.80) | 4.10(2.84-5.65) | 123.18(86.46-173.30) | 4.13(2.90-5.80) | 0.05(-0.09-0.19) |
| Spain | 567.61(419.02-774.69) | 3.54(2.60-4.83) | 866.84(586.59-1293.03) | 3.50(2.32-5.30) | -0.01(-0.08-0.06) |
| Sri Lanka | 312.28(239.88-411.38) | 4.87(3.78-6.37) | 429.98(285.47-603.84) | 4.22(2.79-5.95) | -0.43*(-0.71--0.14) |
| Sudan | 282.29(185.87-404.72) | 5.34(3.54-7.59) | 695.35(459.10-992.61) | 5.07(3.38-7.17) | -0.16*(-0.21--0.11) |
| Suriname | 5.76(4.14-7.72) | 4.46(3.23-5.96) | 13.36(9.55-18.16) | 5.22(3.72-7.12) | 0.55(-0.32-1.42) |
| Sweden | 151.79(115.74-200.94) | 3.55(2.67-4.74) | 137.63(93.82-197.37) | 3.05(2.07-4.40) | -0.51*(-0.62--0.4) |
| Swiss | 119.17(85.04-168.40) | 3.47(2.47-4.92) | 127.10(79.50-199.13) | 3.01(1.87-4.74) | -0.52*(-0.7--0.33) |
| Syrian Arab Republic | 156.71(114.10-210.07) | 5.30(3.89-7.06) | 320.98(229.98-444.01) | 4.65(3.31-6.48) | -0.44*(-0.6--0.29) |
| Taiwan (Province of China) | 452.44(384.67-532.27) | 6.09(5.25-7.09) | 765.75(637.10-928.38) | 5.87(4.85-7.15) | 0(-0.12-0.11) |
| Tajikistan | 55.96(38.71-75.32) | 4.46(3.17-5.91) | 145.70(109.20-190.84) | 3.95(2.99-5.16) | -0.41*(-0.59--0.22) |
| Thailand | 1234.45(963.60-1568.85) | 5.92(4.66-7.48) | 2394.72(1798.59-3126.42) | 6.55(4.90-8.59) | 0.38*(0.14-0.62) |
| Timor-Leste | 10.36(7.35-14.37) | 4.38(3.14-6.03) | 18.79(13.11-25.63) | 4.37(3.06-5.94) | -0.08(-0.28-0.13) |
| Togo | 35.34(23.40-52.73) | 4.02(2.71-5.93) | 158.21(98.36-247.96) | 5.53(3.45-8.63) | 1.03*(0.95-1.12) |
| Tokelau | 0.02(0.01-0.03) | 4.49(3.12-6.44) | 0.02(0.02-0.03) | 4.00(2.78-5.72) | -0.37*(-0.56--0.18) |
| Tonga | 0.91(0.64-1.29) | 3.40(2.42-4.82) | 1.32(0.92-1.88) | 3.62(2.51-5.15) | 0.20*(0.1-0.29) |
| Trinidad and Tobago | 20.37(15.13-27.77) | 4.78(3.58-6.47) | 36.58(26.35-50.37) | 5.49(3.95-7.56) | 0.47*(0.12-0.82) |
| Tunisia | 86.68(63.36-119.11) | 3.66(2.71-4.96) | 242.45(168.08-342.59) | 4.14(2.86-5.87) | 0.38*(0.34-0.43) |
| Turkey | 1016.88(749.26-1345.85) | 5.58(4.13-7.35) | 1804.66(1325.68-2456.31) | 4.32(3.16-5.90) | -0.84*(-0.99--0.68) |
| Turkmenistan | 30.65(24.49-39.03) | 3.21(2.60-4.03) | 88.53(65.01-115.77) | 4.12(3.03-5.38) | 0.91*(0.48-1.34) |
| Tuvalu | 0.18(0.13-0.24) | 5.41(3.95-7.15) | 0.24(0.17-0.33) | 5.17(3.74-7.09) | -0.15*(-0.2--0.11) |
| Uganda | 144.51(95.58-218.11) | 3.78(2.54-5.67) | 441.56(291.96-630.95) | 4.11(2.75-5.83) | 0.25*(0.13-0.37) |
| Ukraine | 772.00(592.10-1020.96) | 3.62(2.78-4.77) | 896.95(642.82-1182.73) | 4.06(2.91-5.36) | 0.49(-0.15-1.12) |
| United Arab Emirates | 38.98(26.22-55.92) | 5.06(3.46-7.17) | 330.80(213.67-498.66) | 3.49(2.25-5.27) | -1.18*(-1.54--0.81) |
| United Kingdom | 1004.18(762.71-1334.75) | 3.93(2.97-5.24) | 1241.40(942.02-1629.35) | 4.11(3.09-5.43) | 0.11(-0.13-0.35) |
| United Republic of Tanzania | 239.03(171.11-329.15) | 3.91(2.84-5.33) | 675.73(458.16-972.95) | 3.84(2.62-5.50) | -0.03(-0.13-0.06) |
| United States of America | 4436.82(3604.81-5524.33) | 3.99(3.27-4.93) | 5410.79(4858.05-6043.42) | 3.76(3.37-4.21) | -0.18*(-0.3--0.05) |
| United States Virgin Islands | 3.71(2.72-4.97) | 7.18(5.23-9.68) | 2.57(1.82-3.61) | 7.09(4.99-10.01) | 0.05(-0.24-0.34) |
| Uruguay | 35.83(29.43-45.10) | 2.89(2.37-3.64) | 52.26(41.06-67.53) | 3.41(2.66-4.42) | 0.58*(0.31-0.85) |
| Uzbekistan | 159.03(113.39-217.05) | 3.03(2.18-4.10) | 396.60(296.72-528.45) | 2.68(2.01-3.55) | -0.37(-0.87-0.13) |
| Vanuatu | 2.47(1.58-3.50) | 5.68(3.67-8.01) | 6.15(4.32-8.25) | 5.50(3.88-7.37) | -0.11(-0.24-0.02) |
| Venezuela (Bolivarian Republic of) | 272.98(197.65-378.13) | 4.66(3.42-6.39) | 738.25(522.11-1033.51) | 5.75(4.06-8.06) | 0.67*(0.36-0.98) |
| Viet Nam | 885.04(635.60-1228.82) | 5.30(3.88-7.24) | 2797.78(1990.87-3951.21) | 5.78(4.11-8.17) | 0.29*(0.21-0.36) |
| Yemen | 148.15(98.41-214.88) | 4.91(3.30-7.06) | 501.57(327.45-724.66) | 4.88(3.20-7.02) | 0(-0.2-0.21) |
| Zambia | 77.78(56.59-104.51) | 4.21(3.10-5.59) | 251.51(176.01-349.73) | 4.41(3.12-6.08) | 0.14*(0.05-0.23) |
| Zimbabwe | 103.91(77.37-138.60) | 4.22(3.18-5.57) | 339.16(228.16-476.30) | 6.69(4.51-9.39) | 1.52*(1.15-1.89) |

Note: UI: uncertainty interval; CI, confidence interval; AAPC, average annual percent of change; DALY, disability-adjusted life years; ASDR, age-standardized DALYs rate
